# A Genomic Map of the Effects of Linked Selection in Drosophila

**DOI:** 10.1371/journal.pgen.1006130

**Published:** 2016-08-18

**Authors:** Eyal Elyashiv, Shmuel Sattath, Tina T. Hu, Alon Strutsovsky, Graham McVicker, Peter Andolfatto, Graham Coop, Guy Sella

**Affiliations:** 1 Department of Ecology, Evolution, and Behavior, Hebrew University of Jerusalem, Jerusalem, Israel; 2 Department of Biological Sciences, Columbia University, New York, New York, United States of America; 3 Department of Ecology and Evolutionary Biology and the Lewis-Sigler Institute for Integrative Genomics, Princeton University, Princeton, New Jersey, United States of America; 4 The Laboratory of Genetics and The Integrative Biology Laboratory, Salk Institute for Biological Studies, La Jolla, California, United States of America; 5 Department of Evolution and Ecology, University of California, Davis, Davis, California, United States of America; Institute of Science and Technology Austria (IST Austria), AUSTRIA

## Abstract

Natural selection at one site shapes patterns of genetic variation at linked sites. Quantifying the effects of “linked selection” on levels of genetic diversity is key to making reliable inference about demography, building a null model in scans for targets of adaptation, and learning about the dynamics of natural selection. Here, we introduce the first method that jointly infers parameters of distinct modes of linked selection, notably background selection and selective sweeps, from genome-wide diversity data, functional annotations and genetic maps. The central idea is to calculate the probability that a neutral site is polymorphic given local annotations, substitution patterns, and recombination rates. Information is then combined across sites and samples using composite likelihood in order to estimate genome-wide parameters of distinct modes of selection. In addition to parameter estimation, this approach yields a map of the expected neutral diversity levels along the genome. To illustrate the utility of our approach, we apply it to genome-wide resequencing data from 125 lines in *Drosophila melanogaster* and reliably predict diversity levels at the 1Mb scale. Our results corroborate estimates of a high fraction of beneficial substitutions in proteins and untranslated regions (UTR). They allow us to distinguish between the contribution of sweeps and other modes of selection around amino acid substitutions and to uncover evidence for pervasive sweeps in untranslated regions (UTRs). Our inference further suggests a substantial effect of other modes of linked selection and of adaptation in particular. More generally, we demonstrate that linked selection has had a larger effect in reducing diversity levels and increasing their variance in *D*. *melanogaster* than previously appreciated.

## Author Summary

One of the major discoveries in modern population genetics is the profound effect that natural selection on one locus can have on genetic variation patterns at linked loci. Since the first evidence for linked selection was uncovered in *Drosophila melanogaster* over two decades ago, substantial effort has focused on quantifying the effects and on distinguishing the relative contributions of purifying and positive selection. We introduce an approach to jointly model the effects of positive and negative selection along the genome and infer selection parameters. To this end, we consider how closely linked each neutral site is to different types of annotations and substitutions. When we apply the inference method to genome-wide data from 125 *D*. *melanogaster* lines, our model explains most of the variance in diversity levels at the megabase scale and allows us to distinguish among the contribution of different modes of selection on proteins and UTRs. More generally, we provide a map of the effects of natural selection along the genome, and show that selection at linked sites has had an even more drastic effect on diversity patterns than previously appreciated. We also make a tool available to apply this approach in other species.

## Introduction

Selection at one site distorts patterns of polymorphism at linked neutral sites, acting as a local source of genetic drift. While the qualitative effects of “linked selection” are undisputed, quantifying them and understanding their source has been one of the central challenges in evolutionary genetics over the past two decades [1–17].

Indeed, characterizing the effects of linked selection is of central importance in many contexts. If linked selection introduces substantial heterogeneity in rates of coalescence along the genome, then obtaining accurate estimates of demographic parameters requires a genomic map of these effects [18,19]. Such maps would also serve as improved null models for other population genetic inferences, such as scans for recent targets of adaptation that rely on outlier approaches [20–22]. Moreover, an accurate characterization of the effects of linked selection carries extensive information about the selective pressures that shape genome evolution. Understanding how the effects vary among taxa would also inform long-standing questions about the determinants of levels of genetic diversity and genetic load within species [23,24,25, 26].

Patterns of genetic variation are informative about natural selection at linked sites because the effects of linked selection vary with the mode and parameters of selection. For instance, “classic” selective sweeps, in which a newly-arisen beneficial mutation is quickly driven to fixation, reduce genetic variation at nearby sites over a scale that depends on the strength of selection and rate of recombination [2,3]. Other modes of adaptation, including partial and soft sweeps, cause similar, although more subtle effects [27–31]. Background (purifying) selection against deleterious mutations also reduces diversity levels at linked sites over a scale that depends on the strength of selection and rate of recombination but to an extent that depends on the density of selected sites [5,8,9,32–34].

Until recently, evidence for the effects of linked selection was sought in the relationships between diversity patterns and factors that are expected to influence the strength and frequency of selection [13–15,17]. For example, both positive and negative linked selection should have a greater effect in regions with lower recombination rates, because, on average, a neutral site would be linked to more selected sites. Consistent with this expectation, diversity levels are positively correlated with rates of recombination in *Drosophila melanogaster* and several other species [4,35,36]. By a similar argument, linked purifying selection should be stronger in regions with a greater density of functional sites (e.g., coding regions) and the effects of sweeps should be greater in regions with more functional substitutions (e.g., non-synonymous substitutions). In accordance with these expectations, diversity levels decrease with the density of amino acid substitutions in Drosophila species [11,12] and in humans [37], and decrease with the density of coding and putatively functional non-coding regions in Drosophila [38], humans [18,35,37] and other species (e.g., [39,40] and cf. [17]).

Beyond providing compelling evidence for the importance of linked selection, these relationships can be used to estimate selection parameters [6,10–12]. These inferences, however, suffer from severe limitations. First, it is difficult to distinguish between the effects of different modes of linked selection, with two decades of effort focused on distinguishing the effects of classic selective sweeps from those of background selection [5,7,10,14,17,31]. Second, even when a specific mode of selection is assumed, some parameters remain poorly identifiable (e.g., the rate and strength of beneficial substitutions in sweep models [10,14]). These inferences also appear to be strongly affected by the genomic scale over which they are evaluated [14].

An alternative approach is to take advantage of spatial diversity patterns along the genome. Pioneering efforts in *D*. *melanogaster* used estimates of the genome-wide rate of deleterious mutations, genetic maps, and the spatial distribution of constrained genomic regions, to demonstrate that background selection could account for changes in diversity levels along chromosomes as well as for differences in diversity levels between X and autosomes ([41–43]). More recently, McVicker et al. [18] used ancestral diversity levels along the genome in order to build a map of the effects of background selection along the human genome. The central idea was to calculate the probability that a neutral site is polymorphic, given its genetic distance from conserved coding regions and the rate of deleterious mutation and distribution of selection effects at these regions; selection parameters were then estimated by maximizing the composite-likelihood for neutral polymorphisms along the genome. Although based on limited data, the map inferred by this approach provides an impressive fit to diversity patterns on the mega-base scale. However, the associated estimate of the deleterious mutation rate is unreasonably high, more than four-fold greater than estimates of the total spontaneous mutation rate [44–47], possibly reflecting the absorption of the effects of background selection from other, poorly annotated functional regions or the effects of positive selection [18].

Another recent approach to learn about selective sweeps relies on plots of the average levels of diversity as a function of distance from amino acid substitutions throughout the genome [48–50]. Assuming that some of the substitutions resulted from classic sweeps, we would expect a trough in diversity levels around substitutions, with the depth related to the fraction that were beneficial and the width (in units of genetic distance) reflecting the strength of selection. The rate and strength of classic sweeps can thus be inferred from the shape of the trough. Applying this methodology to data from *D*. *simulans*, Sattath et al. [48] found a trough in neutral diversity levels around amino acid substitutions that extended over ~15 kb, but not around synonymous substitutions (which served as a control). The collated plot approach has several limitations, however. First, application of the same approach to human data [49] suggests that background selection, which is concentrated in or near coding regions, may contribute to the troughs in diversity, and thus could bias estimates of positive selection parameters. Second, inferences based on collated diversity patterns account only for the average clustering of amino acid substitutions and not for their spatial distribution around every neutral site.

Here, we combine the advantages of these two recent approaches [18,48] in order to infer selection parameters and build a genomic map of the effects of linked selection, considering background selection and classic selective sweeps jointly. We model the effects of background selection using the annotations for linked sites, and those of classic sweeps by considering linked, putatively functional sites that experienced a substitution. The method is applicable to genome-wide polymorphism data, allowing for information to be combined across samples. As an illustration, we apply our method to genome-wide resequencing data from 125 lines of *Drosophila melanogaster* (from the DGRP [51]). We also make software available for the approach to be applied more broadly.

## Materials and Methods

### The model and inference method

We model the effects of background selection and classic sweeps on neutral heterozygosity (i.e., the probability of observing different alleles in a sample size of two), *π*, at an autosomal position *x*. In a coalescent framework, the model takes the form

$$\pi (x)=\frac{2u(x)}{2u(x)+1/(2N_{e}B(x))+S(x)},$$ (1)

where *u*(*x*) is the local mutation rate, *N*_*e*_ is the effective population size without linked selection, *B*(*x*) is the local (multiplicative) reduction in the effective population size due to background selection and *S*(*x*) is the local coalescence rate caused by classic sweeps. This approximation can be arrived at by considering the probability that a mutation occurs (at a rate 2*u*(*x*) per generation) before our pair of lineages are forced to coalesce by either genetic drift (1/2*N*_*e*_*B*(*x*)), which includes the effect of background selection, or by a selective sweep (*S*(*x*)). While we consider autosomes, the model can be extended to sex chromosomes with minor modifications.

**Fig 1:**
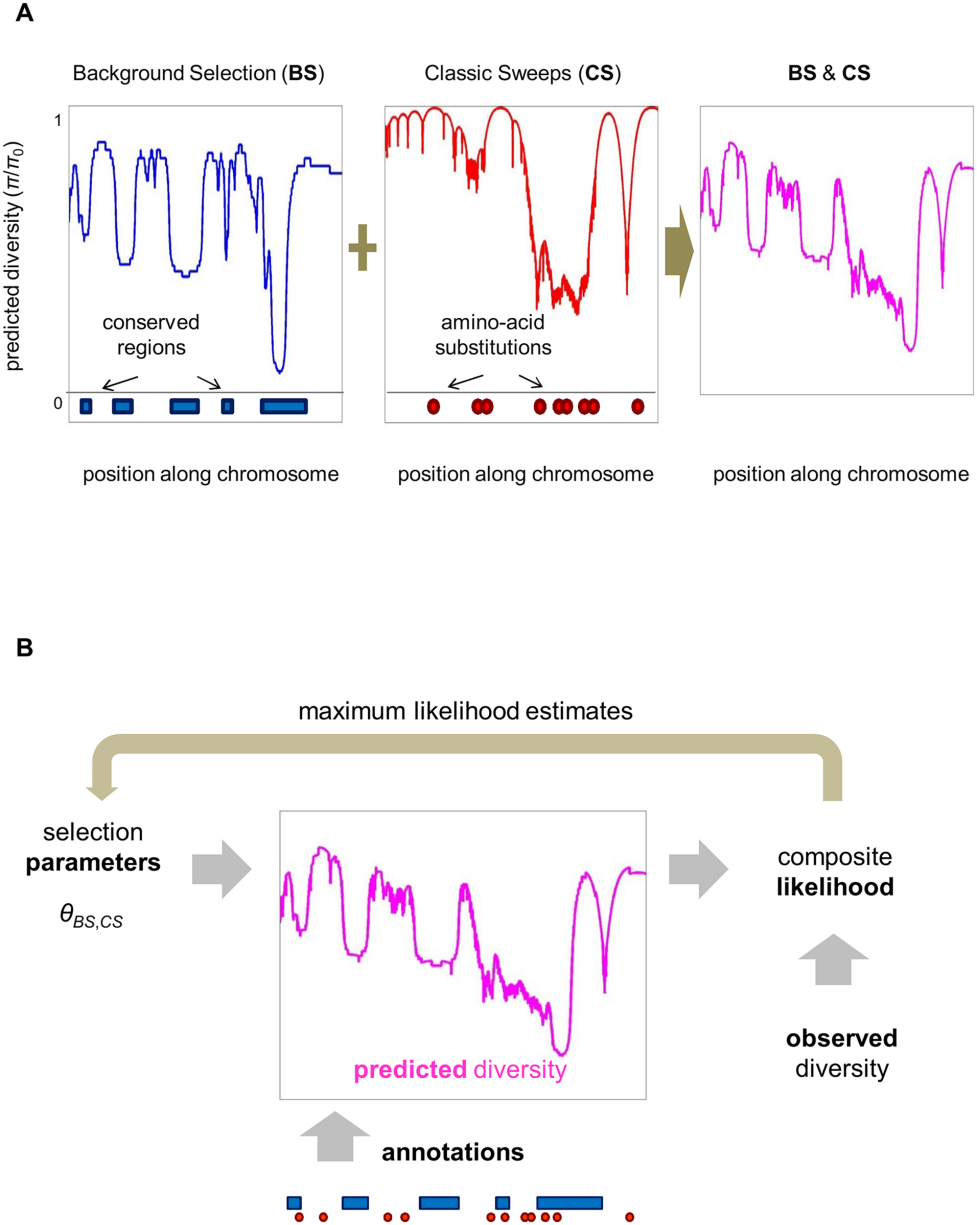
Constructing a map of the effects of linked selection and inferring the underlying selection parameters. (**A**) The expected neutral heterozygosity is estimated for each position in the genome, given the positions and selection parameters of different annotations. (**B**) To estimate selection parameters, their composite likelihood is maximized given the set of annotations and neutral polymorphism data throughout the genome.

The model for the effects of background selection, *B*(*x*), follows Hudson & Kaplan [8] and Nordborg et al. [9] (Fig 1A). We assume a set of distinct annotations *i*_*B*_ = 1,…,*I*_*B*_ under purifying selection (e.g., exons, UTRs, introns and intergenic regions) and positions in the genome *A*_*B*_ = {*a*_*B*_(*i*_*B*_)|*i*_*B*_ = 1,…,*I*_*B*_}, where *a*_*B*_(*i*_*B*_) denotes the set of genomic positions with annotation *i*_*B*_. The selection parameters at these annotations are given by Θ_*B*_ = {(*u*_*d*_(*i*_*B*_),*f*(*t*|*i*_*B*_))|*i*_*B*_ = 1,…,*I*_*B*_}, where *u*_*d*_ is the rate of deleterious mutations and *f*(*t*) is the distribution of selection coefficients in heterozygotes. The reduction in the effective population size is then

$$B(x|A_{B},\Theta_{B},R)=Exp\left(-\sum_{i_{B}}\sum_{y\in a_{B}(i_{B})}\int \frac{u_{d}(i_{B})}{t{\left(1+r(x,y)(1-t)/t\right)}^{2}}f(t|i_{B})dt\right),$$ (2)

where *R* is the genetic map, *r*(*x*, *y*) is the genetic distance between the focal position *x* and positions *y* (only positions on the same chromosome are considered). The integral reflects the effect that a site under purifying selection at position *y* exerts on a neutral site at position *x*. This expression and its combination across sites should provide a good approximation to the effect of background selection so long as selection is sufficiently strong (i.e., when 2*N*_*e*_*t*>>1).

In turn, the model for the effect of selective sweeps follows from an approximation used by Barton [52] and Gillespie [53], among others (Fig 1A). Similarly to the model for background selection, we assume a set of distinct annotations *i*_*S*_ = 1,…,*I*_*S*_ subject to sweeps, but here we know the specific positions at which substitutions have occurred, *A*_*S*_ = {*a*_*S*_(*i*_*S*_)|*i*_*S*_ = 1,…,I_*s*_}, with *a*_*S*_(*i*_*S*_) denoting the set of substitution positions with annotation *i*_*S*_. The selection parameters at these annotations are Θ_*S*_ = {(*α*(*i*_*S*_),g(*s*|*i*_*S*_))|*i*_*S*_ = 1,…,*I*_*S*_}, where *α* is the fraction of substitutions that are beneficial and *g*(*s*) is the distribution of their additive selection coefficients. For autosomes, the expected rate of coalescent per generations at position *x* due to sweeps is then approximated by

$$S(x|A_{S},\Theta_{S},R,{\bar{N}}_{e},T)=\frac{1}{T}\sum_{i_{S}}\alpha (i_{S})\sum_{y\in a(i_{S})}\int Exp\left(-r(x,y)\tau (s,{\bar{N}}_{e})\right)g(s|i_{S})ds,$$ (3)

where *T* is the length of the lineage (in generations) over which substitutions occurred, the positions of substitutions *y* are summed over the chromosome with the focal site, ${\bar{N}}_{e}$ is the average effective population size and *τ*(*s*,*N*_*e*_) is the expected time to fixation of a beneficial substitution with selection coefficient *s* and given an effective population size *N*_*e*_. We use the diffusion approximation for the fixation time

$$\tau (s,N_{e})=\frac{2(\text{ln}(4N_{e}s)+\gamma -{\left(4N_{e}s\right)}^{-1})}{s},$$ (4)

where *γ* is the Euler constant (cf. [28]). This model relies on several simplifying assumptions and approximations. In particular, the term 1/*T* relies on an assumption of one substitution per site per lineage and neglects variation in the length of lineages across loci. In combining the effects over substitutions, we further assume that the timings of beneficial substitutions are independent and uniformly distributed along the lineage, and that they are infrequent enough such that we can ignore interference among them [54]. The exponent approximates the probability of coalescence of two samples due to a classic sweep with additive selection coefficient *s* (where 2*N*_*e*_*s*>>1) in a panmictic population of constant effective size ${\bar{N}}_{e}$. (We consider the effects under more general sweep models later.) In principle, we should use the local *N*_*e*_ incorporating the effects of background selection but given the logarithmic dependence of Eq (3) on *N*_*e*_, we simply use the average.

To infer the selection parameters Θ_*B*_ and Θ_*S*_, we use a composite likelihood approach across sites and samples [55] (Fig 1B). We denote the positions of neutral sites by *X* and the set of samples by *I*. We then summarize the observations by a set of indicator variables across sites and all pairs of samples *O* = {*O*_*i*,*j*_(*x*) | *x* ∈ *X*, *i* ≠ j ∈ *I*}, where *O*_*i*,*j*_(*x*) = 1 indicates that samples *i* and *j* (*i*≠*j*) differ at position *x* and *O*_*i*,*j*_(*x*) = 0 indicates that they are the same. In these terms the composite log-likelihood takes the form

$$LogL=\sum_{x\in X}\sum_{i\neq j\in I}\text{log}\left(\text{Pr}\left\{O_{i,j}(x)|\Theta_{B},\Theta_{S}\right\}\right),$$

where

$$\text{Pr}\left\{O_{i,j}(x)|\Theta_{B},\Theta_{S}\right\}=\{\begin{array}{cc} \pi (x|\Theta_{B},\Theta_{S}) & O_{i,j}(x)=1\\ 1-\pi (x|\Theta_{B},\Theta_{S}) & O_{i,j}(x)=0 \end{array}.$$ (5)

Using composite likelihood circumvents the complications of considering linkage disequilibrium (LD) and the more complicated forms of coalescent models with larger sample sizes. Importantly, maximizing this composite likelihood should yield unbiased point estimates [56,57]. Beyond losing the information in LD patterns and in the site frequency spectrum, the main cost of this approach is the difficulty in assessing uncertainty in parameter estimates (as standard asymptotics do not apply). We therefore use other ways to assess the reliability of our inferences.

To make the composite likelihood calculations (i.e., the calculation of *π*(*x*|Θ_*B*_,Θ_*S*_)) feasible genome-wide, we discretize the distribution of selection coefficients on a fixed grid. Given a grid of negative and positive selection coefficients, *t*_*g*_ and *s*_*k*_, *g* = 1,…,*G* and *k* = 1,…,*K*, the distribution of selection coefficients for each annotation becomes a set of weights on this grid, *w*(*t*_*g*_| *i*_*B*_) and *w*(*s*_*k*_| *i*_*S*_). (In principle, the grid could also be annotation-specific.) For background selection, these weights reflect the rate of deleterious mutations with a given selection coefficient and their sum should therefore be bound by the maximal deleterious mutation rate per site. For sweeps, the weights reflect the fraction of beneficial substitutions with a given selection coefficient and their sum should be bound by 1. In these terms, the effect of background selection takes the form

$$B(x|\Theta_{B})=Exp\left(-\sum_{i_{B}}\sum_{g=1}^{G}w(t_{g}|i_{B})b(x|t_{g},i_{B})\right),$$ (6)

where *Exp*(−*b*(*x*|*t*_*g*_, *i*_*B*_)) is the proportional reduction in the effective population size induced by having one deleterious mutation per generation per site with selection coefficient *t*_*g*_ at all the positions in annotation *i*_*B*_. By the same token, the effects of sweeps take the form

$$S(x|\Theta_{S})=\frac{1}{T}\sum_{i_{S}}\sum_{k=1}^{K}w(s_{k}|i_{S})s(x|s_{k},i_{S}),$$ (7)

where $\frac{1}{T}s(x|s_{k},i_{S})$ is the probability of coalescence per generation induced by sweeps in annotation *i*_*S*_, if all the substitutions in this annotation are beneficial with selection coefficient *s*_*k*_. Thus, by using a grid, we can calculate a lookup table of *b*(*x*|*t*_*g*_, *i*_*B*_) and *s*(*x*|*s*_*k*_, *i*_*S*_) once and then use it to calculate the likelihood for a given set of weights. Moreover, the interpretation of estimated distributions on a grid is arguably simpler than that of the continuous parametric distributions commonly used (e.g., gamma and exponential), for which densities associated with different selection coefficients are highly interdependent. In the Supplementary Material (S1B Text), we describe additional simplifications in the calculation of *b*(*x*|*t*_*g*_, *i*_*B*_) and *s*(*x*|*s*_*k*_, *i*_*S*_).

Other parameters are estimated as follows. Consider Eq (1) rewritten as

$$\pi (x)=\frac{\pi_{0}\cdot (u(x)/\bar{u})}{\pi_{0}\cdot (u(x)/\bar{u})+1/B(x)+S(x;{\bar{N}}_{e},T)},$$ (8)

to clearly specify all the additional parameters required for inference. $\pi_{0}\equiv 4N_{e}\bar{u}$ is (approximately) the average neutral heterozygosity, given the effective population size in the absence of linked selection and the average mutation rate per site ($\bar{u}$); *π*_0_ is estimated through the likelihood maximization. The local variation in mutation rate $u(x)/\bar{u}$ is estimated by averaging substitution patterns at putatively neutral sites among closely related species in sliding windows, with a window size chosen to balance true variation in mutation rates and measurement error (see S1B Text). Finally, ${\bar{N}}_{e}$ is estimated based on the average genome-wide heterozygosity at putatively neutral sites, after dividing out by a direct estimate of the spontaneous mutation rate per site, and $T/2{\bar{N}}_{e}$ is estimated by $(\bar{K}/2)/\pi_{0}$, where $\bar{K}$ is the average number of substitutions per neutral site on the lineage.

The software package implementing the inference and construction of the map of the effects of linked selection is available online (http://github.com/sellalab/LinkedSelectionMaps). In the Supplementary Material (S1B Text), we describe the steps that were taken to check the proper convergence of the likelihood maximization.

### Application to data from Drosophila

We apply our method to population resequencing data from *Drosophila melanogaster*. The data analyses are briefly described here, with further details provided in S1A Text. As a proxy for neutral variation, we use synonymous polymorphism within *D*. *melanogaster*, based on resequencing data from the Drosophila Genetic Reference Panel (DGRP) [51] consisting of 162 inbred lines derived from the Raleigh, North Carolina population. The rate of synonymous divergence used to control for local variation in mutation rates is estimated using the aligned reference genomes of *D*. *simulans* and *D*. *yakuba* [58]. As potential targets of selection (annotations), we use coding regions, untranslated, transcribed regions (UTRs), long introns (>80bp) and intergenic regions, downloaded from FlyBase [59] (http://flybase.org, release 5.33), all of which have been inferred to be under extensive purifying selection in *D*. *melanogaster* [60–63], and which together cover ~98.5% of the euchromatic genome. Substitutions that occurred in these annotations on the *D*. *melanogaster* lineage since the common ancestor with *D*. *simulans* are inferred from a three-species alignment of reference genomes from *D*. *melanogaster*, *D*. *simulans* and *D*. *yakuba* [58]. We do not include substitutions in intergenic regions, which are not included in the three-species alignment, and our treatment of missing data, e.g., due to gaps in the alignment, is detailed in S1B Text.

For the genetic map, we rely on estimates of the cM/Mb rates recently published by Comeron et al. [64]. Because our inferences are sensitive to errors in the genetic map in regions of low recombination, we exclude the distal 5% of chromosome arms (in which rates are known to be low in *D*. *melanogaster*) and regions with a sex-averaged recombination rate below 0.75cM/Mb.

We perform the inference under a variety of selection models. In the Results, we primarily compare the models incorporating classic sweeps, background selection or both, including all of the annotations listed above using a grid of selection coefficients which consists of five point masses on a log-linear scale, with *t* and *s* = 10^−5.5^, 10^−4.5^, 10^−3.5^, 10^−2.5^ and 10^−1.5^. Our maps of the effects of linked selection corresponding to the model incorporating both classic sweeps and background selection are available online (http://github.com/sellalab/LinkedSelectionMaps/melanogaster_maps). In the Supplementary Material we study the sensitivity of our results to: selection on synonymous mutations—using a subsets of synonymous differences (S1H Text), the recombination thresholds (S1H Text), the grid of selection coefficients (S1I Text), and to using subsets of annotations (S1I Text) and an upper bound on the deleterious mutation rate (S1E Text).

## Results

### Maps of the effects of linked selection along the genome

**Fig 2:**
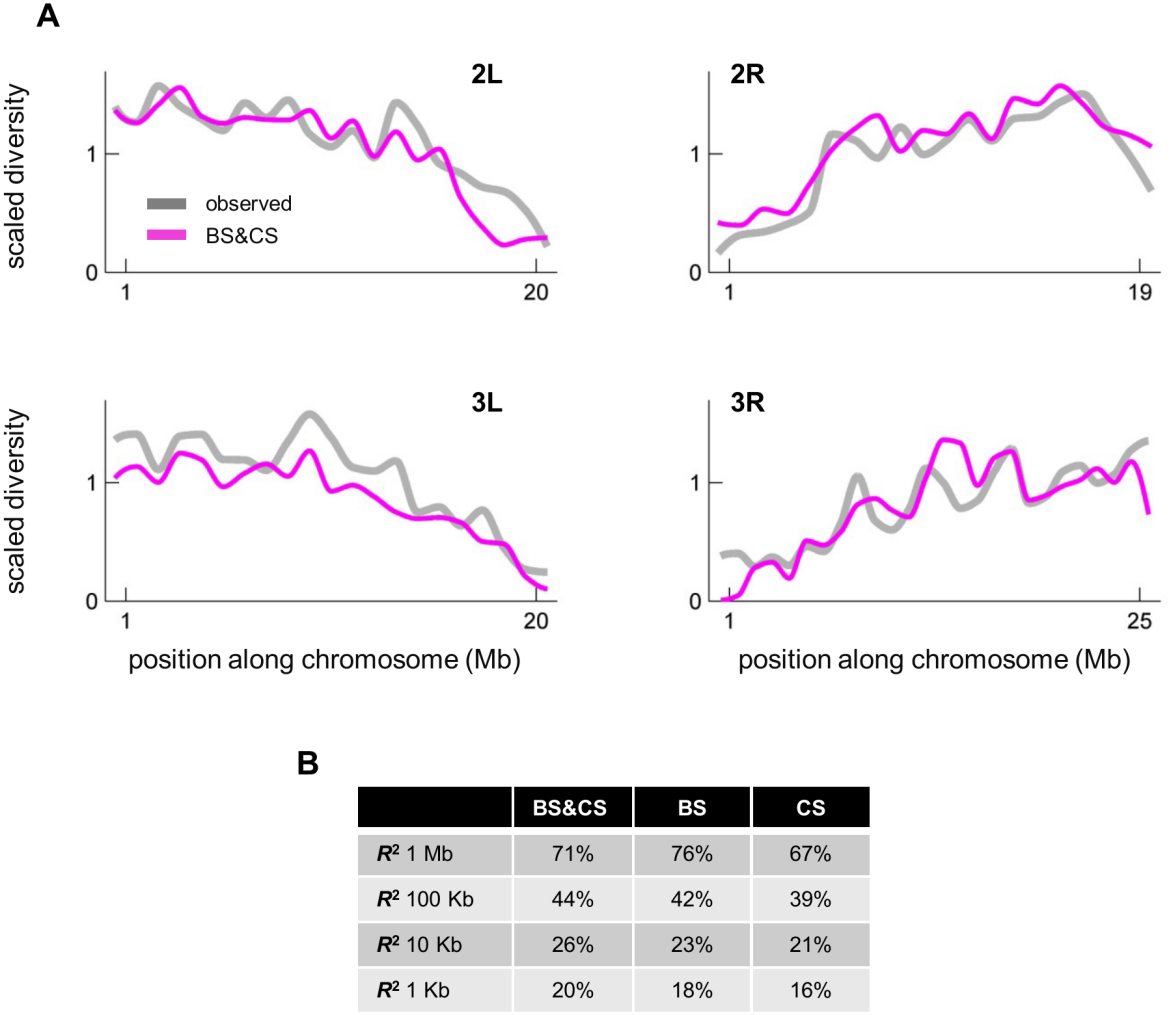
A comparison of observed and predicted scaled diversity levels along the major autosomes of *Drosophila melangaster*. Throughout, we refer to “scaled diversity” as synonymous heterozygosity divided by synonymous divergence, to control for variation in the mutation rate (as detailed in S1C Text); scaled diversity is shown relative to the genome average. (**A**) Observed and predicted scaled diversity over non-overlapping 1 Mb windows across chromosomal arms. (**B**) Summaries of the goodness of fit for models including background selection (BS), classic sweeps (CS) and both (BS & CS). *R*^2^ is calculated for autosomes using non-overlapping windows of different sizes. Selection parameters are inferred using synonymous sites with recombination rate >0.75cM/Mb, while the predictions and corresponding summaries are calculated for sites with recombination rate >0.1cM/Mb.

Our inference yields a map of the expected neutral diversity levels at every position along the genome. One way to evaluate these predictions is to compare them with observed diversity levels (Fig 2). A quantitative comparison at the 1Mb scale suggests that our map accounts for 71% of the variance (*R*^2^) in diversity levels of non-overlapping autosomal windows. To address the concern that the high R^2^ is the result of over-fitting, we perform a *leave-one-out cross-validation* (LOOCV) analyses [65] in which we divide the genome into non-overlapping 1Mb windows, using only data outside a window to make our predictions about diversity levels in it (S1C Text; Table S2 in S1 Text). This analysis shows that over-fitting has a negligible effect on our prediction, which is to be expected: while our model has many parameters (36), the data set is much larger (consisting of 1.7×10^6^ codons, and levels of linkage disequilibrium are low).

In interpreting the fit, both model misspecification and the stochasticity inherent to the evolutionary process need to be considered. Importantly, even if our model provided an accurate description of the processes generating genetic diversity, we would not expect a perfect fit to the data because of the randomness of the processes being modeled. Notably, our model assumes that a substitution at a given annotation could have occurred with uniform probability at any time along the *D*. *melanogaster* lineage and that it had a certain probability of being beneficial with a given selection coefficient. Any evolutionary realization of the model would have that substitution occur at a particular time—more often than not, too far in the past to affect extant diversity patterns—and with a given selection coefficient, thus generating considerable variance in predicted diversity levels at linked sites. In addition, both genealogical and mutational processes are stochastic. Averaging over 1Mb windows partially reduces this stochasticity and in that regard, it is not surprising that our predictions become less precise when we use smaller windows (Fig 2B). However, even with 1Mb windows, we would still expect considerable variance in diversity levels around the expectation.

In addition, although we assume that the genetic maps and annotations are known, there is error in both. Imprecision of the genetic map and imperfect annotations (e.g., our clumping together of all coding, UTR, intronic and intergenic substitutions and regions) decrease our predictive ability. As genetic maps and annotations become better, we should therefore expect our predictions to improve. Another class of assumptions relates to processes that we did not model, including changes in population size [61,66,67]. In spite of many potential factors contributing to noise in our predictions, the fit to data is very good.

In the Supplementary Materials (S1F Text) we compare our predictions to those based on a map of the effects of background selection generated using the methodology developed by Charlesworth [41] and recently extended by Charlesworth [42] and Comeron [43]. This approach differs from ours in several ways, most notably in being based on estimates of selection parameters from the literature, which themselves do not rely on the effects of linked selection on diversity patterns. While it performs impressively well at the 1Mb scale (though not as well as ours) the quality of the predictions becomes much worse than ours as the scale becomes smaller (Table S5 in S1 Text). (Note that Comeron [43] uses rank correlations to evaluate his predictions; the explained variance using rank correlations are much higher than the quantitative predictions we use here, which is why his result might appear comparable at first sight.)

Using *R*^2^ values for window sizes varying from 1kb to 1Mb, we can ask which model(s) are best supported. We find that the one combining both background selection and classic sweeps almost always does better than the models with a single mode of selection (Fig 2). Our leave-one-out cross-validation analysis confirms that this finding is not the result of over-fitting in the combined model (Table S2 in S1 Text; see S1C Text for details). Thus, our combined model of the effects of linked selection captures much of the variation in diversity levels at the mega-base scale, and provides an improved null model in scans for targets of positive selection or for the purposes of demographic inference. Because using *R*^2^ has its limitations, we use a variety of other statistical approaches to evaluate our inferences in the sections that follow.

### The effects of linked selection around different annotations

We can also use our analysis to learn about the effects of linked selection for different annotations. If a feature is enriched for targets of purifying or positive selection, then we expect to see a reduction in diversity levels around it due to linked selection. Collating diversity levels around all instances of a feature averages over confounding effects at specific genomic positions as well as over the inherent stochasticity in diversity levels, allowing us to isolate the selection effects [18,48–50].

**Fig 3:**
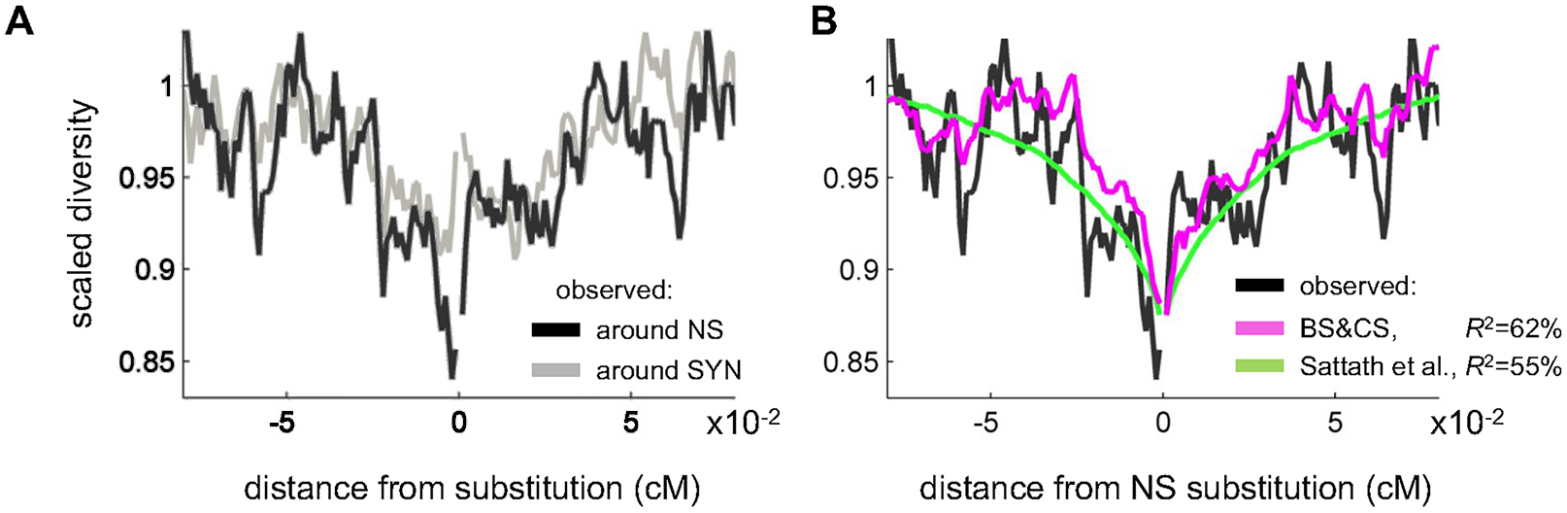
Observed and predicted scaled diversity levels around amino acid substitutions. (**A**) Comparison of scaled diversity levels around non-synonymous (NS) and synonymous (SYN) substitutions. (**B**) Comparison of predicted, scaled diversity levels based on our method and that of Sattath et al. (2011) [48].

We first consider how diversity levels vary with genetic distance from amino acid and synonymous substitutions (Fig 3). There is a trough in diversity around both, but the one around amino acid substitutions is substantially deeper (Fig 3A). Fig 3B compares the predicted diversity levels around amino acid substitutions based on Sattath et al. [48] and our inference. A rough quantitative comparison suggests that our method fits the data better than that of Sattath et al. (*R*^2^ = 62% for our method compared to *R*^2^ = 56% for Sattath et al.; see S1G Text for more details). Moreover, the new method also predicts more of the detailed variation in diversity levels, presumably because it accounts for the statistical properties of genome architecture, e.g., the density of coding regions at given genetic distances up or downstream of substitutions.

**Fig 4:**
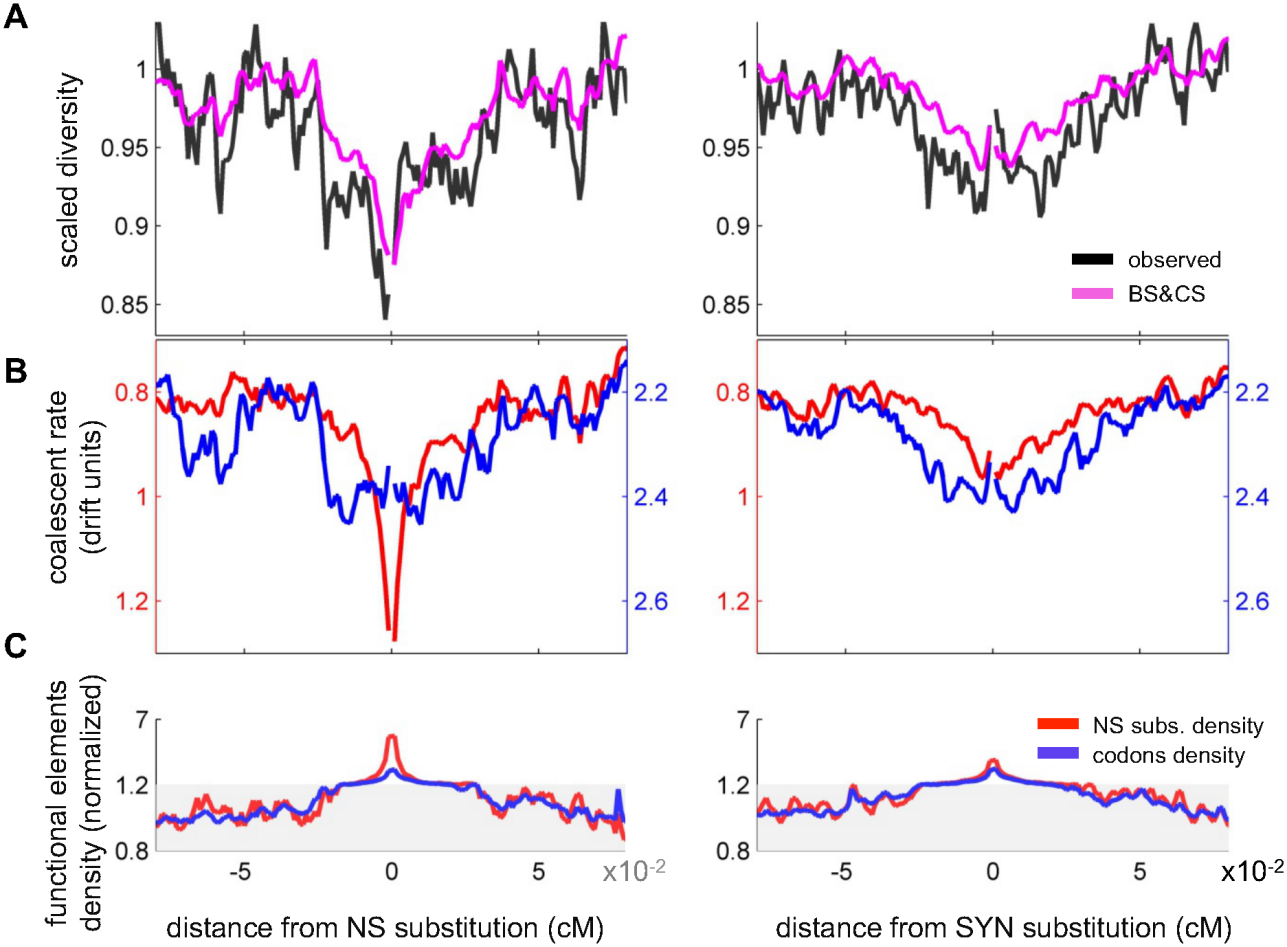
The contribution of background selection and classic sweeps to scaled diversity levels around non-synonymous and synonymous substitutions. (**A**) Observed and predicted scaled diversity levels around non-synonymous (left) and synonymous (right) substitutions. The predictions are based on the joint model for background selection and classic sweeps. (**B**) The contribution of background selection (blue) and classic sweeps (red) measured in terms of the coalescent rates that they induce. The rates are measured in units of 1/2*N*_*e*_, where *N*_*e*_ is our estimate of the effective population size in the absence of linked selection. To make these graphs comparable to the scaled diversity levels in (A), with lower rates corresponding to higher scaled diversity levels, the direction of the y-axis is reversed. (**C**) The density of exonic sites (blue) and non-synonymous substitutions (red) as a function of distance from non-synonymous and synonymous substitutions. Densities are normalized by the average densities at distance >0.06cM; the shaded areas correspond to the use of a different linear scale.

In principle, our approach should allow us to tease apart the contributions of classic sweeps and background selection to these diversity patterns (Fig 4). Comparing the predictions of each model alone is less informative for this purpose, because when only one is considered, it likely absorbs some of the effects of the other (see next section). In contrast, with the inference based on the combined model, the contribution of each mode should be identifiable from its specific functional forms and annotations. When we focus on the contribution of background selection (blue lines in Fig 4B), we see a reduction in diversity around both synonymous and non-synonymous substitutions because both types of substitutions occur in coding regions, in which background selection effects are strongest (e.g., [18,68]). Moreover, because the density of coding regions and other annotations (blue lines in Fig 4C and Fig S6 in S1 Text) is similar around the two kinds of substitutions, the shape and magnitude of the reductions in diversity are also similar (blue lines in Fig 4B). In contrast to background selection, the reduction around non-synonymous substitutions due to classic sweeps is much greater than for synonymous substitutions (red lines in Fig 4B). This results not only from the focal non-synonymous substitution but also (and primarily) from the greater density of non-synonymous substitutions near a focal non-synonymous substitution than around a synonymous one (red lines in Fig 4C). Whereas the clustering of non-synonymous substitutions around synonymous substitutions primarily reflects the greater density of coding sites, the clustering around non-synonymous substitutions (beyond the focal amino acid substitution) presumably reflects correlated evolution of nearby residues and other adaptive processes (e.g., [69]).

These findings illustrate that, at least as modeled, background selection and classic sweeps are identifiable. Intuitively, the information about classic sweeps at non-synonymous substitutions comes from the comparison of neutral diversity levels between sites near many non-synonymous substitutions versus near few, given a similar density of other annotations. After properly accounting for the effects of classic sweeps, information about the background selection pressure exerted by exons comes from contrasting the diversity levels among regions that vary in the density of codons but are otherwise similar. In practice, we do not learn about these processes in a stepwise fashion, as presented here, but instead maximize the probability of the data considering all of the annotations simultaneously.

We can therefore use these findings to revisit the enduring question of the relative contribution of background selection and classic sweeps to shaping diversity patterns (Fig 4). In particular, the negative correlation between diversity levels and the density of non-synonymous substitutions previously reported in Drosophila [11,12] likely reflects a substantial contribution of background selection in addition to positive selection. In contrast, the greater reduction in diversity levels at non-synonymous compared to synonymous substitutions in Drosophila is almost entirely the outcome of classic sweeps [48]. A caveat is that the parameter estimates obtained from the approach based on collated plots likely absorb some of the effects of background selection and thus overestimate the effects of linked selection due to sweeps (see next section and Tables S6 and S7 in S1 Text). More generally, in interpreting the results, an important consideration is the presence of other modes of selection that are not modeled explicitly, e.g., soft and partial sweeps. As we discuss at greater length below, our inferences about classic sweeps may reflect a mixture of different kinds of sweeps that result in substitutions while our inferences about background selection may reflect a contribution from other modes of linked selection, including sweeps that do not result in substitutions.

**Fig 5:**
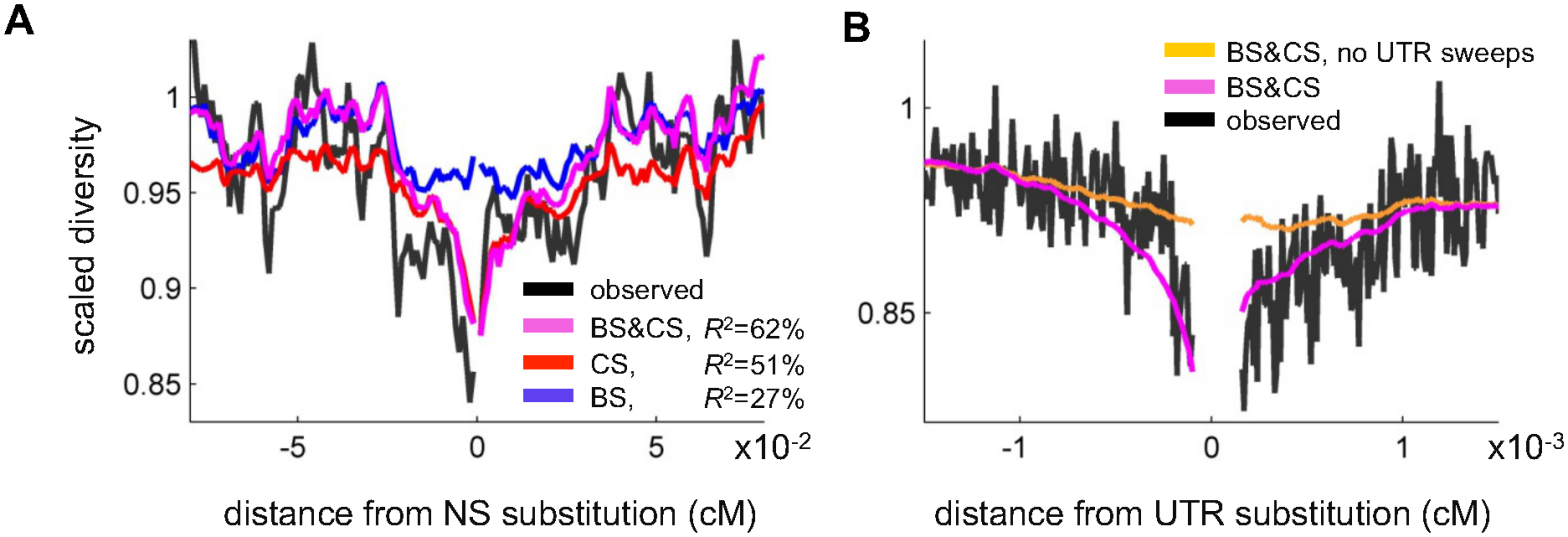
Comparing alternative models around substitutions in proteins and UTRs. (**A**) Comparison of predicted scaled diversity levels around non-synonymous substitutions based on models including background selection (BS), classic sweeps (CS) and both (BS & CS). (**B**) Comparison of predicted scaled diversity levels around substitutions in UTRs based on models with and without sweeps in UTRs.

We can also consider how well the relationships between diversity levels and various genomic features are explained by models with a single mode of selection. As an illustration, Fig 5A shows that the background selection model does better than the model with classic sweeps at predicting diversity levels far from non-synonymous substitutions. Also visually apparent is that, in contrast to the background selection model, the classic sweeps model explains the narrow, deep trough close to non-synonymous substitutions. The combined model does well at predicting diversity levels both close to and far from non-synonymous and synonymous substitutions, again illustrating the need to consider both modes of linked selection in making inferences.

A similar approach can be used to examine the effects of selection acting on non-coding annotations. Notably, our inference suggests that a substantial fraction of substitutions at UTRs lead to classic sweeps (Table S11A in S1 Text and next section). To examine whether this feature of the model is required to explain the data, we look at average diversity levels as a function of genetic distance from substitutions in UTRs (Fig 5B). Our full model does much better at explaining these observations than a model without sweeps at UTRs. This provides the first evidence, to our knowledge, for sweeps at UTRs (or in any non-coding annotation) in Drosophila, and lends strong support to findings of pervasive adaptation in UTRs based on McDonald-Kreitman type approaches and genetic differentiation (*F*_*ST*_) along clines [60,70].

### Estimates of sweep parameters

Our approach also provides estimates of selection parameters. We first consider those obtained for classic sweeps, for which the positions of potential targets of selection (i.e. substitutions) are known. For substitutions at non-synonymous sites and to a lesser extent in UTRs, the ability to localize substitutions and to measure diversity levels using nearby synonymous sites provides us with high spatial resolution about selection effects on diversity patterns.

If we exclude background selection from the model, the only notable difference is the addition of a probability mass of strong selection coefficients at amino acid substitutions (~0.3% of substitution with *s* = 10^−1.5^), which affects diversity levels on a broad scale, in effect retracing large-scale variation in recombination rate and, to a lesser extent, coding density. When background selection is included in the model, this spatial effect becomes entirely associated with background selection (Fig S3 in S1 Text). This suggests that under a model of sweeps alone, the extra mass is absorbing some of the effects of other modes of selection that are not driven by substitutions.

In turn, under our combined models, the distribution of selection coefficient exhibits two dominant masses: ~4% of substitutions appear to have been strongly selected (*s*≈10^−3.5^) and 35–45% of substitutions weakly so (*s* between 10^−5.5^–10^−6^; the ranges reported here and below correspond to grids of selection coefficients with 5 and 11 point masses; see S1I Text). The effects of both masses on diversity levels can be clearly seen in collated plots around substitutions (cf. Fig S8 in S1 Text) and accord with previous studies [48,71]. At UTRs, we find that 25–45% of substitutions are associated with weak to intermediate strength of selection (*s*≈10^−4.5^–10^−5.5^). While the effects of sweeps at UTRs are apparent in Fig 5B, our quantitative estimates are associated with greater uncertainty than those for non-synonymous substitutions because we have lower spatial resolution near substitutions at UTRs (see S1H Text). At long introns, we infer that none of the substitutions were driven by sweeps; this estimate, however, might also reflect low power in these regions, because we measure diversity levels at synonymous sites that are, on average, far from intronic substitutions (see S1I Text).

Intriguingly, our estimates of the fraction of beneficial substitutions in proteins and UTRs accord with those based on extensions of the McDonald-Kreitman test (i.e., between ~40–85% for amino acids and 30–60% from UTRs [12,38,60,72–74]), when previous estimates based on the effects of sweeps on polymorphism data were substantially lower [11,48]. A caveat is that this conclusion only holds when we include the contribution of weakly selected substitutions. Our inference about weakly selected substitutions is based on diversity patterns very close to substitutions (roughly equivalent to 50 bp on average) and at these distances, considerable uncertainty about the genetic map and limited polymorphism data preclude us from distinguishing between selection coefficients ranging between 10^-5.5^ and 10^−6^. Because selection coefficients at the lower end of this range could be nearly neutral, the substitutions could partially reflect the fixation of slightly deleterious mutations rather than beneficial ones and more generally compensatory evolution [75]. We note further that our approach is not necessarily expected to agree with McDonald-Kreitman based estimates, which reflect adaptive rates over different time scales (i.e., on the order of *N*_*e*_ in our case [76], as opposed to the time scale of divergence). These reservations notwithstanding, our approach suggests that properly accounting for weakly selected substitutions leads to a convergence of estimates based on linked selection and McDonald-Kreitman based approaches, and provides, to our knowledge, the first corroboration of these elevated estimates.

With recent research highlighting the potential role of modes of adaptation other than classic sweeps, e.g., partial and soft sweeps [27–31,77–80], which we do not model explicitly, it is natural to ask how they might affect our inferences. To a first approximation, the effects of other kinds of sweeps on diversity levels around the selected site can be viewed as a superposition of the effects of classic sweeps with varying selection coefficients at different distances from the selected site (see [31,81] and S1D Text). This property implies that our parameter estimates for classic sweeps can be translated into rates and strengths of other types of sweeps.

As an example, consider our estimates that ~4% of amino acid substitutions were driven by selection coefficients of *s* = 10^−3.5^ and ~35% by a selection coefficient of 10^-5.5^. An approximately similar effect on diversity levels along the genome could be explained by assuming that 39% of substitutions are caused by partial sweeps that are driven to a frequency of *x* = 0.34 with a selection coefficient of *s* = 10^−3.9^, then to fixation with a selection coefficient of *s* = 10^−5.8^ (see S1D Text). Similar parameter estimates could also be generated by mixtures of partial and full sweeps, described by the fraction of full and partial sweeps and associated selection coefficients and distributions of frequencies (*x*) for each kind of partial sweep. In S1D Text, we detail how other kinds of sweeps (soft, from multiple mutations or standing variation, or on recessive alleles) would be recorded by our approach and thus how the effects of mixtures of sweeps would translate into our parameter estimates.

In other words, in the presence of different kinds of sweeps, our parameter estimates reflect the effects of the mixture on diversity levels around substitutions. A given set of estimates designates a continuous class of mixtures and, in principle, one can write down equations for the parametric family of mixtures that would yield the same estimates. Further narrowing down the underlying mixtures, however, will require developing inferences that use other aspects of the data.

### Estimates of background selection parameters

Parameter estimates for purifying selection are fairly insensitive to the exclusion of classic sweeps from our model (e.g., Table S5 in S1 Text). When we do not impose an upper bound on the rate of deleterious mutations, we observe two main selection strengths, both of which are localized in exons and UTRs. The dominant one is extremely strong selection (*s* = 10^-1.5^), which affects diversity over a spatial scale of ~4Mb (or ~7cM, the distance at which the diversity levels reach 90% of baseline levels). As noted previously, such selection coefficients lead diversity levels to follow large-scale variation in recombination rate and to a lesser extent coding density. In this regard, it is important to note that we have to rely on relatively crude annotations, rather than accounting for the fine-scale location of sites under purifying selection within each annotation. As a result, our inference is likely to capture an average effect over considerably larger spatial scales than is actually the case, thereby leading to somewhat inflated selection coefficients (akin to what is seen for classic sweeps when background selection is not considered).

The strong selection coefficient is also associated with unreasonably high estimates of the deleterious mutation rate, which far exceed direct estimates of the total mutation rate (by 4-9-fold in exons and UTRs; Table S12 in S1 Text) [82]. A plausible interpretation is that these high rates reflect the absorption of linked selection effects that evade direct capture by our inference. For example, they might absorb the effects of sweeps at introns (or intergenic regions) that evade our inference because of the crude annotation of substitutions in these regions. They might also absorb the effects of other modes of linked selection, which are not modeled explicitly. Notably, population genetic models of quantitative traits suggest that the response to changing selection pressures could involve many soft and partial sweeps that do not result in fixation [83,84] and therefore would not be included in our estimates for classic sweeps. The effects of such soft and partial sweeps on diversity levels can be similar to those of background selection [31,81,85,86]. Moreover, because we lack localized annotations for such sweeps (when they do not result in fixation), we would tend to associate them with stronger selection coefficients of background selection, whose effects on diversity are less localized. If this interpretation is correct, then our inference suggests that modes of linked selection other than classic sweeps and background selection have a substantial effect on diversity levels around coding regions.

We also find evidence for somewhat weaker purifying selection (centered around *s* = 10^−3.5^) associated with a more realistic deleterious mutation rate (e.g. ~50–60% of the overall mutation rate in exons), but which may still reflect a contribution from other forms of linked selection. These values are in agreement with those obtained for exons by approaches that do not rely on the signatures of linked selection (cf. [42,43], and S1F Text). Purifying selection of this strength should affect diversity levels on spatial scale of ~40 kb (or 0.07cM, defined as above), a footprint that is visible in our analyses of diversity levels around synonymous and non-synonymous substitutions (blue lines in Fig 4B).

In the Supplementary Material (S1E and S1F Text), we present additional analyses that support this interpretation of background selection parameters, based on models in which we impose a biologically plausible upper bound on the deleterious mutation rate and use the modeling approach of Charlesworth [41,42].

### The impact of linked selection on diversity levels

We next examine the extent to which linked selection decreases the mean and increases the variance in diversity levels throughout the genome. The average reduction quantifies the effects of linked selection on the effective population size, a key parameter for many aspects of genome evolution [24,25]. The heterogeneity in diversity levels is of interest because it quantifies the deviation from the uniform neutral null model that is implicitly assumed in most, if not all, demographic inferences and scans for targets of adaptation.

**Fig 6:**
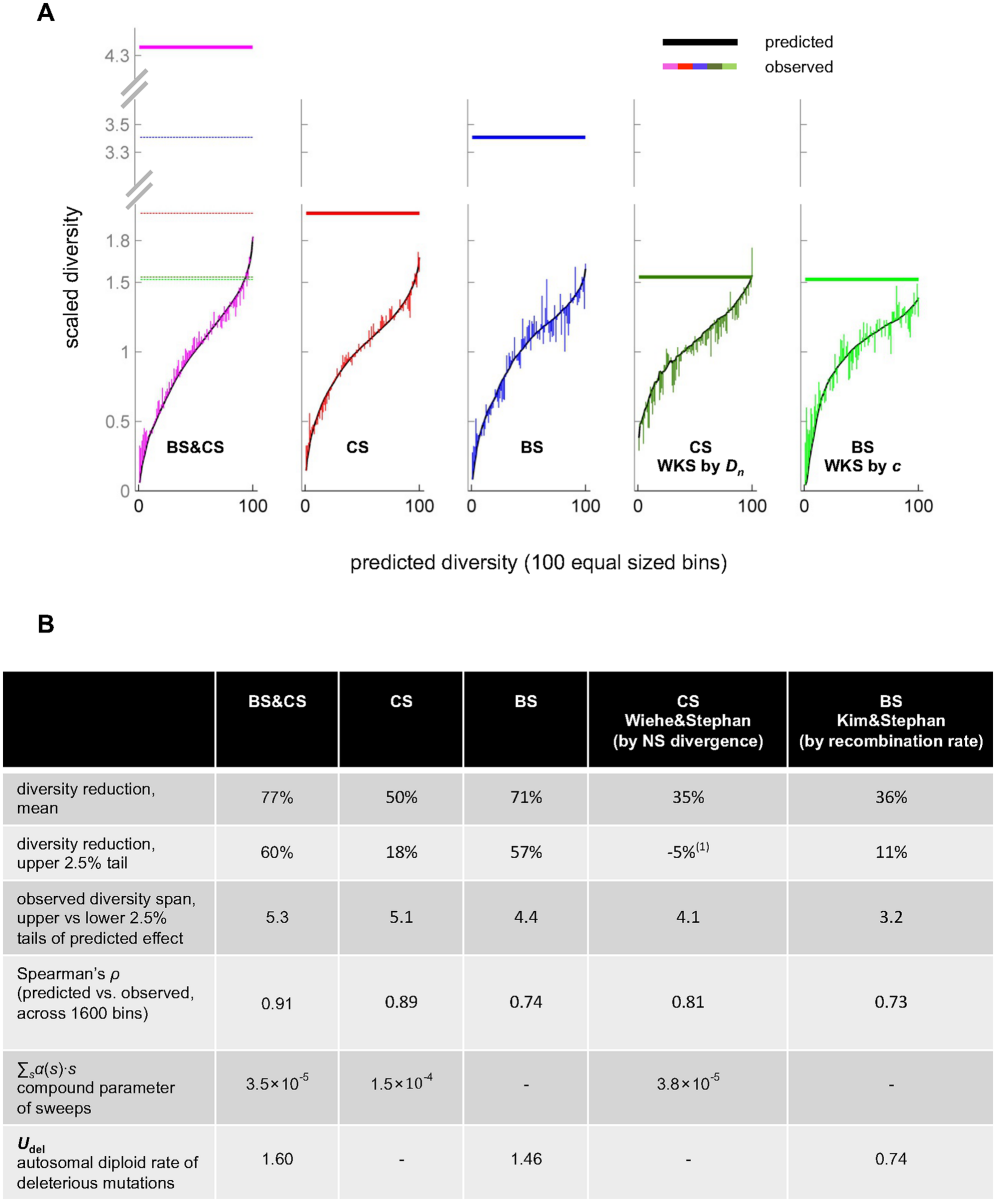
The impact of linked selection on scaled diversity levels. (**A**) Observed scaled diversity levels stratified by model predictions. Shown here are the results based on our method with both background selection and classic sweeps (pink), background selection alone (blue) and classic sweeps alone (red), as well as for the Wiehe and Stephan (1993) [6] method for classic sweeps based on the density of non-synonymous substitutions (dark green) and the Kim and Stephan (2000) [10] method for background selection based on recombination rates (light green). The stratification is described in the text. Predicted levels are shown in black, the observed deviations from the predictions are shown as vertical lines, with the colors corresponding to different models, and the estimated scaled diversity levels in the absence of linked selection are shown as horizontal bars. (**B**) Summaries of the mean reduction and heterogeneity in scaled diversity levels based on the different methods and models. Also shown are estimates of compound selection parameters and the Spearman correlation between predicted and observed levels. (1) The negative value reflects the fact that the observed scaled diversity level is higher than the level predicted in the absence of linked selection.

We focus on the impact of linked selection in coding regions with recombination rates above 0.1cM/Mb, because our predictions become less reliable in regions with lower recombination rates (see S1H Text). To this end, we sort genomic positions according to their predicted levels of diversity (Fig 6A). For 1600 bins with equal amounts of data, the concordance between observed and predicted levels is extremely high (Spearman *ρ* = 0.91), indicating that the variation predicted by our model is real (and not due to over-fitting; Table S2 in S1 Text). Sorting based on our predictions, we find substantial variation in the observed diversity levels across bins (approximately five-fold difference between the upper and lower 2.5%; Fig 6B). Moreover, we see that the effects of linked selection are visible across all bins, rather than being restricted to bins with lower expected diversity. In other words, almost no region in the genome is free from the effects of linked selection (with the exception of the correlation coefficient, none of these results are sensitive to the number of bins).

We quantify the average reduction due to linked selection as the ratio of the average observed diversity level, $\bar{\pi }$, to the predicted level without linked selection, *π*_0_. Doing so indicates an average reduction of 77%-89% in neutral diversity levels genome-wide (excluding low-recombination regions for which the reduction should be greater). Strikingly, even in the upper 1%-tile, linked selection is predicted to have reduced diversity levels by ~60–80%. Given the uncertainty about the parameter estimates associated with strong purifying selection (S1E Text and Table S4 in S1 Text), our inferences about *π*_0_ may not be robust, however. Indeed, imposing a plausible bound on the rate of deleterious mutations results in fits that are only marginally worse but dramatically affects our estimates of *π*_0_ (reducing it from 4.4 fold times the observed mean to 2.8-fold, with 5 point masses; S1E Text and Table S4 in S1 Text). In brief, this follows from the fact that strong selection affects diversity levels on broad spatial scales, leaving little signal of localization, and thus similar observed diversity levels can result from different combinations of deleterious mutation rates and *π*_0_ values. Unfortunately, we cannot observe *π*_0_ directly. What we can say, based on our stratification, is that linked selection reduces average diversity levels by at least two-fold (Fig 6A).

Our estimates suggest much stronger effects of linked selection than do previous methods. Notably, when we apply previous methods based on the relationship between diversity levels and rates of recombination or functional divergence [6,10–12,26] (see S1G Text for details), we infer an average reduction in diversity levels that lies between 34–36%, with no reduction in the upper 1%-tile of predicted diversity levels (Fig 6B and Table S12 in S1 Text). Comparing the stratification of diversity levels by the various methods (Fig 6A and 6B) indicates these previous methods do worse at predicting diversity levels, span a smaller range of diversity levels and under-estimate the effects of linked selection; specifically, their predictions of *π*_0_ are lower than the upper 1%-tile of observed diversity levels based on our stratification (Fig 6A and 6B). The reason is that by relying on a single genomic feature (e.g., recombination rate) and averaging over others (e.g., non-synonymous divergence), these methods overlook much of the variation in diversity levels caused by linked selection, causing their estimates to suffer from the equivalent of regression toward the mean (the same problem applies to their estimated selection parameters; see S1G Text). A similar “averaging out” effect takes place when we consider a model with background selection or classic sweeps alone (Fig 6A and 6B).

This line of argument implies that even with the combined model, we still underestimate the heterogeneity in diversity levels because of imperfect annotations. Notably, this would be the case if our inferences about background selection are likely absorbing substantial effects of other modes of linked selection but are unable to capture them in full, let alone to do so with high spatial resolution. Thus, the heterogeneity in diversity levels due to linked selection in the *Drosophila melanogaster* genome is likely to be even greater than we have inferred. Similar speculation about the average reduction in diversity levels is more difficult, given the uncertainty associated with our parameter estimates for background selection (Tables S4 and S10 in S1 Text). What we can say is that our lower bound based on stratification is likely to increase as annotations improve.

## Discussion

### The relative contribution of different modes of linked selection

Over two decades of research have aimed to quantify the relative contributions of classic sweeps and background selection in shaping diversity patterns. If these were the only modes of linked selection, then we would now have an answer. We have shown that the contributions of background selection and classic sweeps are identifiable using our inference and, with the stated caveats about the effects of partial annotations, we can quantify their relative contributions. Based on the combined model and using the genome-wide average rates of coalescence induced by each mode of selection as a measure of their relative contribution, our findings would suggest that background selection has a ~1.6–2.5-fold greater effect than classic selective sweeps (Table S3 in S1 Text; see S1C Text for details and other metrics).

The question is complicated, however, by the contribution of other modes of linked selection. Our results strongly suggest that inferences about background selection include a major contribution of other modes of linked selection, plausibly the result of sweeps that do not result in substitutions. In turn, our inferences for classic sweeps may reflect a combination of different kinds of sweeps. These results echo other theoretical and empirical results highlighting the importance of other modes of positive selection, notably of partial and soft sweeps [27–31,77–81].

The question about the relative contribution of different modes of linked selection can therefore be rephrased in terms of the contributions of background selection, classic sweeps and other modes of linked selection. If we assume that our combined model fully accounts for the reduction in diversity levels due to linked selection and that the effects of background selection are captured by our inferences excluding the strong selected mass, then 12% of the increase in coalescence rate due to linked selection is the result of background selection (estimates in this paragraph correspond to the model with 5 point masses). Further assuming that our inferences about classic sweeps can reflect any combination of classic and other kinds of sweeps resulting in fixation, and that the remaining effects are the outcome of other modes of linked selection, then we would conclude that roughly 0 to 29% of coalescent events are due to classic sweeps and the remaining 88 to 59%, respectively, are due to other modes of linked selection.

### Implications for Drosophila and other taxa

Despite unresolved questions about linked selection, the maps do well at predicting diversity levels at the 1Mb scale (Fig 2), the substantial stratification of diversity levels throughout the genome (Fig 6) and the diversity patterns around different annotations (Figs 3, 4 and 5). This predictive ability is explained in part by the effects of linked selection already well captured by our current approach, e.g., the effects of sweeps that result in substitutions. Also important, however, is the robustness of the inferred map of linked selection to model misspecification. For instance, our map performs well even though the effects of background selection may reflect a substantial contribution of other modes of linked selection and despite an averaging effect owing to the imprecise annotations. Moreover, at this scale, the performance is fairly insensitive to variations of the model (e.g., imposing a bound on the deleterious mutation rate), suggesting that these features play a relatively minor role. Thus, while the spatial resolution of maps of linked selection in Drosophila (and other taxa) is expected to improve with better genetic maps, annotations and models, we can already do quite well. One implication is that our approach already generates substantially improved null models for population genetic inferences about demography and scans of selection.

The reliability of our inferences about selection critically depends on well-localized annotations and on the distance between these annotations and the putatively neutral sites used to measure diversity levels. For these reasons, we obtain reliable estimates for sweeps resulting in substitutions at exons and UTRs and distinguish their contribution from other forms of linked selection, but cannot achieve similarly reliable estimates for other modes and annotations. It follows that in applications to other species, we would expect the reliability of estimates to depend both on the quality of annotations and on genome architecture. Human data may be particularly well suited, as there are higher-resolution annotations as well as phylogeny-based information about conservation in both coding and non-coding regions. In addition, properties of the genome architecture, notably the lower density of selected regions [87], may help to distinguish effects of different annotations and modes of linked selection.

In both Drosophila and humans, one area that will need further work is the inclusion of other modes of selection. In that regard, it is interesting to note that our results mirror similar finding in humans: inferences about background selection in McVicker et al. [18] also led to too large a rate of deleterious mutation and work done since suggests that classic sweeps contribute little to the effects of linked selection on genetic variation [49,77,78]. Taken together with other empirical evidence and modeling [27–30,77,79,80,83], these results strongly suggest that other modes of linked selection and of adaptation in particular play a central role in both Drosophila and humans.

It might be difficult to distinguish between different kinds of sweeps based on their footprints around substitutions, especially given the many additional parameters for each if they act in concert (S1D Text). Additional footprints of selection are likely to be needed. Notably, there is likely to be important information about alternative modes of sweeps in diversity levels and patterns of linkage disequilibrium around amino acid polymorphisms [22,80,88].

Another pertinent extension will be to incorporate more realistic demographic assumptions. Like many other methods aimed at quantifying the genome-wide effects of linked selection to date [10,12,18], our model implicitly assumes a panmictic population of constant size. While we focus on a single population, and hence our assumption of random-mating is appropriate, our assumption of a constant size is likely invalid [66,67,89,90]. However, our inference method should be fairly insensitive to changes in the population size, because demographic history should affect different genomic regions similarly, regardless of annotations or other aspects of genomic architecture. Since our method learns about modes of selection and their parameters by contrasting diversity patterns among regions with different properties, it should implicitly control for much of the effects of demography. Having said that, drastic changes in population size could change the efficacy of selection and thus influence our estimates of the distribution of selection coefficients. In addition, regions with different effective population sizes due to linked selection could differ in their transient responses to demographic changes, potentially affecting our inferences. Accounting for these effects is difficult, however. Moreover, existing demographic inferences for North American *D*. *melanogaster* are confounded by the pervasive effects of linked selection. The methods developed here offer a way forward in inferring demography in the presence of linked selection as our map of linked selection could be factored into such analyses.

While these extensions will be important, our current application to Drosophila already reveals that the effects of linked selection are greater than previously assumed, by taking into account spatial features of genome architecture that were previously averaged out. Even excluding low recombination regions, our results suggest high heterogeneity in expected diversity levels due to linked selection (Fig 6B) and an overall reduction in diversity levels of at least two-fold. Applying our approach to other taxa will reveal whether linked selection is having a similarly large effect in other species, and is an important contributor to the apparent disconnect between census and effective population sizes [2,23–26].

## Supporting Information

S1 Text Supporting Online Materials. (DOCX)
