# Supplementary material for: A Genomic Map of the Effects of Linked Selection in Drosophila
Source: PLoS Genet. 2016 Aug 18;12(8):e1006130. doi: 10.1371/journal.pgen.1006130 (PMC4990265; doi:10.1371/journal.pgen.1006130)
Supplement: S1 Text — (DOCX) [file pgen.1006130.s001.docx]

**Supplementary Online Material for**

**A genomic map of the effects of linked selection in *Drosophila***

Eyal Elyashiv^1,2,*^, Shmuel Sattath^1^, Tina T. Hu^3^, Alon Strustovsky^1^, Graham McVicker^4^, Peter Andolfatto^3^, Graham Coop^5^ and Guy Sella^2,*^

1 Department of Ecology, Evolution and Behavior, Hebrew University of Jerusalem, Jerusalem, Israel

2 Department of Biological Sciences, Columbia University, New York, New York, USA

3 Department of Ecology and Evolutionary Biology and the Lewis-Sigler Institute for Integrative Genomics, Princeton University, Princeton, New Jersey, USA

4 Department of Genetics, Stanford University, California, USA

5 Department of Evolution and Ecology, University of California, Davis, California, USA

^*^ Corresponding authors: [eyalshiv@yahoo.com](mailto:eyalshiv@yahoo.com) and [gs2747@columbia.edu](mailto:gs2747@columbia.edu).

Table of Contents

[A. *Drosophila* data set 3](#_Toc405388850)

[Overview 3](#_Toc405388851)

[Genetic maps 3](#_Toc405388852)

[Annotations 4](#_Toc405388853)

[Substitutions and divergence rates 5](#_Toc405388854)

[Polymorphism data 5](#_Toc405388855)

[B. The inference procedure 7](#_Toc405388856)

[Estimating the local mutation rate 7](#_Toc405388857)

[Handling missing data 10](#_Toc405388858)

[Calculating the effects of linked selection 10](#_Toc405388859)

[Likelihood maximization 12](#_Toc405388860)

[C. Statistical analyses 15](#_Toc405388861)

[Details about summaries and figures 15](#_Toc405388862)

[Leave-one-out cross-validation analysis 15](#_Toc405388863)

[Quantifying the relative contribution of background selection and sweeps 16](#_Toc405388864)

[D. Interpreting inferences about classic sweeps 19](#_Toc405388865)

[Partial sweeps 19](#_Toc405388866)

[Soft sweeps (multiple mutations) 20](#_Toc405388867)

[Sweeps from standing variation 21](#_Toc405388868)

[Recessive and other classic sweeps 22](#_Toc405388869)

[A mixture including different kinds of sweeps 23](#_Toc405388870)

[E. Interpreting the inferences about background selection 24](#_Toc405388871)

[Imposing an upper bound on the mutation rate 24](#_Toc405388872)

[Our uncertainty about π_0_ 25](#_Toc405388873)

[F. Comparison with maps based on the Charlesworth approach 27](#_Toc405388874)

[G. Comparison to other inference methods 31](#_Toc405388875)

[Inferring sweep parameters based on the Sattath et al. method. 31](#_Toc405388876)

[An error in Sattath et al. (2011) 33](#_Toc405388877)

[Inferring selection parameters using the Wiehe, Kim and Stephan method 33](#_Toc405388878)

[H. Sensitivity to the recombination rate threshold and to codon usage bias 35](#_Toc405388879)

[The recombination rate threshold 35](#_Toc405388880)

[Codon usage bias 39](#_Toc405388881)

[I. Inference based on additional models 42](#_Toc405388882)

[J. Additional figures and tables 44](#_Toc405388883)

[References 50](#_Toc405388884)

# A. Drosophila data set

Overview*.* Our approach relies on data from neutral sites to estimate the effects of linked selection on diversity levels. As a proxy for neutral sites, we use synonymous variation. Synonymous diversity is measured using re-sequencing data from the *Drosophila* Genetic Reference Panel (DGRP)[1]. The number of synonymous substitutions per codon, used to control for local variation in mutation rates, is estimated from the aligned reference genomes of *D. melanogaster*, *D. simulans* and *D. yakuba* [2]. Among possible choices, synonymous variation reflects a good compromise between the amount of data—since coding regions composing ~20% of the *D. melanogaster* euchromatic genome [3]—and the attempt to minimize the effects of direct selection on the sites [4-8]. In Section H we consider the robustness of our results when instead considering subsets of synonymous polymorphisms that should be even less affected by selection (such as synonymous codon bias).

Our inference further relies on knowledge of the locations and annotations of sites under negative (purifying) and positive selection—the sources of diversity reduction at linked neutral sites. As potential targets of selection, we consider coding regions; untranslated, transcribed regions (UTRs); and long introns and intergenic regions, all of which have been inferred to be under widespread purifying and positive selection in *D. melanogaster* [9-11]. Together, these annotations, which we downloaded from FlyBase [12], cover 98.5% of the euchromatic genome. We consider substitutions along the *D. melanogaster* lineage from the common ancestor with *D. simulans* in any of these annotations (with the exception of synonymous sites, and intergenic regions where we do not have data) to be putative targets of sweeps. We infer the substitutions from the three species alignment of reference genomes from *D. melanogaster*, *D. simulans* and *D. yakuba* [2]. More detail for each of these steps is provided below.

Genetic maps*.* We use the genetic map for *D. melanogaster* estimated by Comeron et al. [13], which is based on ~6000 female meioses, providing estimates at 100 kb resolution (<http://bioweb.biology.uiowa.edu/labs/comeron/recombination/>).

We note that while the genetic map inferred by Chan et al. [14] from patterns of linkage disequilibrium provides higher resolution, we cannot use it for our purposes. Chan et al. infer the population scaled recombination rate *ρ*=4*N_e_c*, where *N_e_* is the local effective population size. Because linked selection causes the effective population size to vary along the genome (and our inference suggests that this variation is considerable; cf. Fig 6 in the main text), the genetic map that they estimate confounds the effects of linked selection with variation in recombination rates, and thus hard to interpret in our inference framework.

Because linked selection affects diversity levels over a scale proportional to 1/*c*, where *c* is the recombination rate per bp, our estimates are sensitive to errors in the recombination rate in regions of low recombination. Moreover, our modeling assumption that interference among selective sweeps is negligible is more likely to be violated in these regions. We therefore exclude neutral polymorphism from regions with a sex-averaged recombination rate below 0.75 cM/Mb as well as centromeric and telomeric regions (i.e., 5% at either end of each arm in physical distance), which have low recombination rates in *D. melanogaster*. We do, however, use the information within these regions (i.e., the positions of annotations and substitutions within them and the number of synonymous substitutions per codon used to control for variation in mutation rates) to generate our predictions of diversity levels. In Section H, we show that our inference is largely robust to the choice of recombination rate threshold.

Annotations**.** The genomic positions for exonic, intronic and untranslated, transcribed regions (UTRs) are from FlyBase [12], for release 5.33 of the *D. melanogaster* genome (<ftp://ftp.flybase.net/genomes/Drosophila_melanogaster/dmel_r5.33_FB2011_01/>). The positions for UTRs are used as is. From the set of intronic regions, we remove short introns (<80 bp), because they appear to be under weak or no selection in *D. melanogaster* [10,15,16]; the remaining positions comprise our “long intron” annotation. In the exonic class, we include only the longest transcript for each gene and, in the rare cases in which genes overlap, we include only the one for which the maximal transcript is longer. All regions between neighboring transcripts are classified as intergenic. Together, the four labels cover 98.5% of the euchromatic autosomal genome, with 18.3% in the exonic class (consisting of 11,447 transcripts), 5.6% in UTRs, 39.2% in long introns, and 35.4% in intergenic regions.

Because we use synonymous polymorphism and divergence to estimate the effects of linked selection on neutral diversity levels, it is especially important to minimize the erroneous identification of codons. To do so, we restrict ourselves to the experimentally validated transcripts in the Gold set of the *Drosophila* Gene Collection (DGC) [17], consisting of 9,358 autosomal longest transcripts and amounting to 3,831,195 codons. Also, we exclude the first (start) and last (stop) codons from each transcript and codons that are split between exons.

Substitutions and divergence rates**.** To identify substitutions in annotations that are possible targets of selection, we rely on the Hu et al. [2] ([http://genomics.princeton.edu/
AndolfattoLab/w501_genome.html](http://genomics.princeton.edu/AndolfattoLab/w501_genome.html)) multiple sequence genic alignments of *D. yakuba*, *D. melanogaster* and *D. simulans* and on their reconstruction of the most recent common ancestor of *D. melanogaster* and *D. simulans*. Specifically, substitutions along the *D. melanogaster* lineage in transcribed regions (i.e., exons, UTRs and long introns) are inferred from sequence differences between the *D. melanogaster* reference genome and its reconstructed ancestor with *D. simulans* (thus neglecting multiple hits). The three species alignment covers only 70% of exons (with 64,205 non-synonymous substitutions, corresponding to 0.0092 per bp), 45% of UTRs (with 153,765 substitutions, corresponding to 0.025 per bp) and 43% of long introns (with 456,401 corresponding to 0.032 per bp). We describe how we handle missing data in Section B.

Because the inferred substitutions are based on a single reference genome from each species, some of them are in fact polymorphic in *D. melanogaster* (~18%), likely resulting in moderate underestimates of the proportion of beneficial substitutions. We use this definition rather than also considering polymorphism data because, under neutrality, conditioning on a site not being polymorphic distorts diversity levels nearby, introducing potential artifacts into our inferences.

We rely on the same multiple species alignment in order to estimate local number of synonymous substitutions per codon and correct for variation in mutation rates along the genome. For this purpose, we use only codons in the Gold set with the aforementioned filters. We estimate the number of synonymous substitutions in non-overlapping window using CODEML (for details see Section B). Specifically, we use the estimate of the number of synonymous substitutions between the common ancestor of *D. simulans* and *D. yakuba* (for which there is an average synonymous divergence of 0.146 synonymous substitutions per codon, or ~0.2 per synonymous site) to minimize statistical dependencies with our polymorphism measurements.

Polymorphism data**.** We measure synonymous polymorphism using data from the *Drosophila* *Genetic Reference Panel* (DGRP) [1] (<http://dgrp.gnets.ncsu.edu/>). In brief, flies were collected from a farmers market in Raleigh, North Carolina, and inbred for 20 generations to generate 162 lines that are mostly isogenic (the average fraction of heterozygous exonic sites per line is 0.4%, with a maximum of 3.3%). The lines were sequenced using Illumina at an average coverage of ~20X and polymorphisms were called using the Joint Genotyper for Inbred Lines (JGIL)[18].

Some of the lines in the DRGP are closely related [14,19]. Given that we assume a random-mating population, we removed a subset of the closely related lines, as follows. First, we calculate the average pairwise differences per site among all pairs of lines. Based on the median of the distribution, we estimate that a threshold of <0.28% per bp corresponding to first cousins (i.e., 7/8 of the median distance across all pairs). We then apply a sequential algorithm in which, at each step, we remove one of the lines from the most closely related pair and recalculate the distribution, until none of the remaining pairs are below the threshold. After this process, 125 lines remain.

The set of codons used to measure pairwise synonymous differences are further filtered as follows. For codons that are heterozygous in a given line, we randomly sample one of the alleles and use it throughout our pairwise comparisons. We further exclude codons that have more than two alleles in our sample or for which the two alleles differ at more than one position (only 6% of polymorphic codons violated these conditions). Lastly, we retain only codons for which we have divergence data (reducing the number of codons by 52%).

After applying the filters, we have polymorphism data at 1,775,362 codons, ~45% of those that met our recombination threshold. At these codons, we have an average sample size of 124 lines, and fewer than 0.5% with sample size <100 lines. Average synonymous heterozygosity per codon is ~0.6% (which is ~0.8% per synonymous site).

# B. The inference procedure

Estimating the local mutation rate**.** We use synonymous divergence data to estimate local mutation rates. First, we divide each chromosome into non-overlapping windows of 1780 bp for estimation (see below for the justification of window size). For each window that contains divergence data for at least 50 codons, we estimate the relative mutation rate at its mid-point by

,

where $\hat{u}$ is our estimate, $\bar{u}$ is the average mutation rate across windows, $\hat{K}_{S}$ is an estimate of the number of synonymous substitutions per codon in the window and $\bar{K}_{S}$ is the number of substitutions per codon, averaged across windows. We estimate $\hat{K}_{S}$ using the three species alignment from Hu et al. [2] for the codons in the window and apply CODEML [20] to obtain the number of substitutions between *D. yakuba* and *D. simulans* (with CODEML parameters: runmode = 0; seqtype = 1; CodonFreq = 2; clock = 0; model = 1; NSsites = 0; icode = 0; fix_kappa = 0; kappa = 1.6; fix_omega = 0; ncatG = 1; getSE = 0; RateAncestor = 2; Small_Diff = 3⨯10^-7^; cleandata = 0; method = 0). Requiring 50 codons or more per window amounts to a relative sampling error of less than ~4%. To obtain point estimates at every genomic position, we use linear interpolation between the two closest flanking estimates.

The choice of window size should minimize sampling error while not masking true variation in mutation rates, as systematic changes in mutation rate near selected annotations (e.g., due to differences in base composition) could potentially bias our estimates of selection parameters. We therefore use a number of approaches to assess the effects of window size. First, we examine how the maximum composite likelihood and *R*^2^ between predicted and observed diversity levels vary with window size (Fig S1A and S1B). These estimates can be viewed as measuring our ability to predict diversity levels along the genome and so we reason that a window size that best captures the true variation in mutation rates would lead to better predictions. The likelihood estimates indicate that window sizes between 1000-3000 bp provide a good balance between true variation and sampling error, and estimates of *R*^2^ are also maximized within this range.

Second, we examine how the choice of window size affects estimates of positive selection parameters, i.e., the fractions of substitutions with a given selection coefficient. As can be seen (Fig S1C), using large window sizes (above our 1780 bp grid point) leads to lower estimates of the fraction of beneficial substitutions with intermediate selection coefficients (e.g., *s*=10^-3.5^). This can be understood as follows. If functional substitutions tend to occur in regions with higher mutation rates, then we would also expect synonymous diversity and the number of substitutions per codon to be elevated in their vicinity. If we use too large a window size, we will fail to capture heterogeneity in mutation rates on spatial scales smaller than the chosen size, such that after dividing out the number of synonymous substitutions per codon, scaled diversity levels near substitutions at these smaller scales would appear to be greater than they should be. The result would be an underestimation of the effects of sweeps on these spatial scales, or more precisely, an underestimation of the fraction of sweeps with weak selection coefficients. Our window size of 1780 bp is within the range suggested by the goodness-of-fit statistics (Fig S1A) but is also sufficiently small such that our estimates for the fraction of beneficial substitutions corresponding to different selection coefficients appears to be stable. We note that there may well be mutation rate heterogeneity below this scale, which may lead us to slightly underestimate the proportion of substitutions associated with weak sweeps.


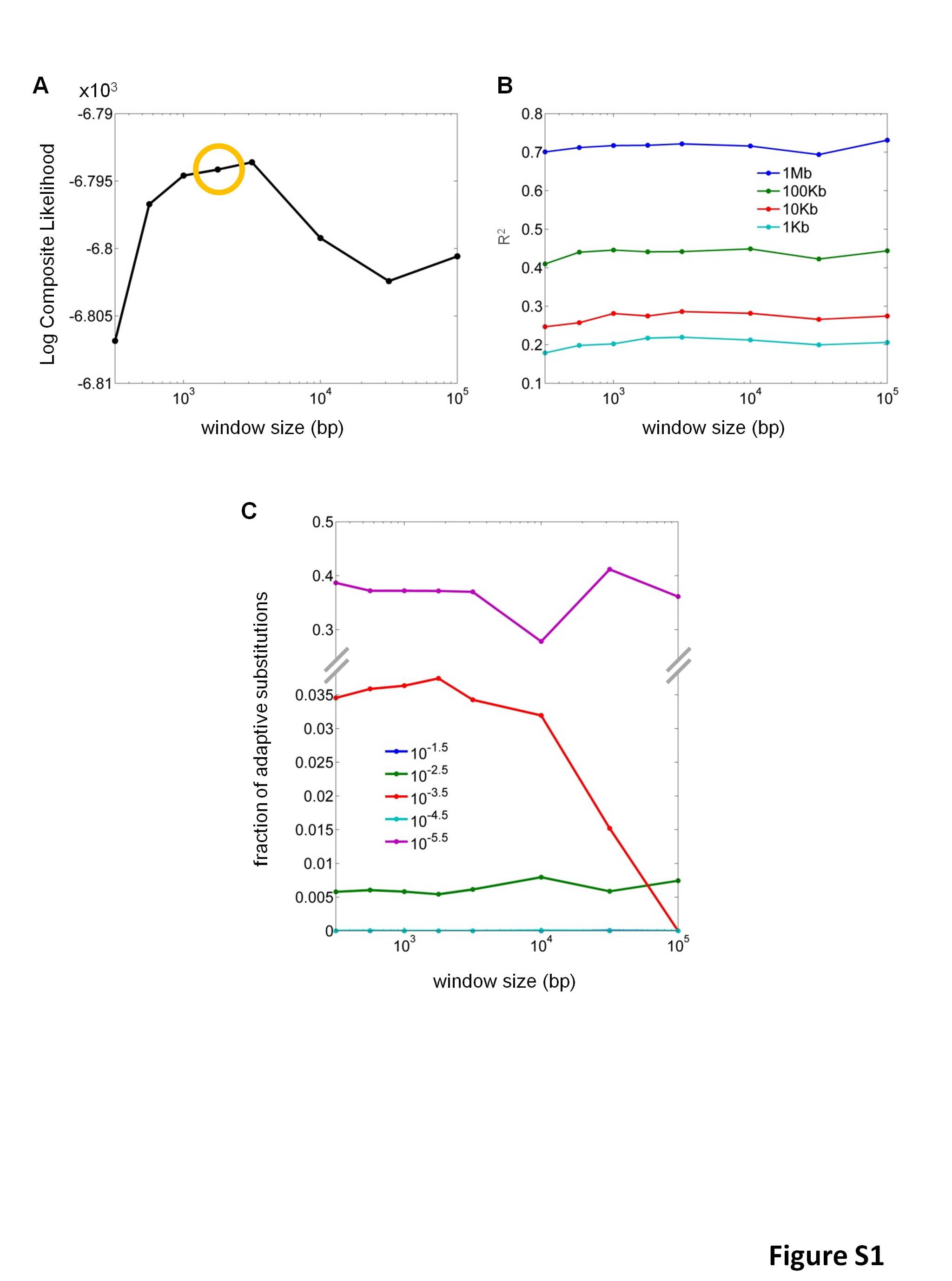


**Fig S1. Choosing a window size in which to estimate the mutation rate.** (**A**) The maximum likelihood as a function of window size. (**B**) *R*^2^ as a function of the choice of window size, over several scales. (**C**) Estimated fractions of beneficial amino-acid substitutions with a given selection coefficient as a function of window size, for different selection coefficients. In all cases, we use the model combining background selection and classic sweeps. The circles denote the window size.

Handling missing data. Due to incomplete alignments and the application of quality filters, the positions of putatively selected substitutions cover only a subset of the genome. Yet substitutions in regions of missing data are likely to impact neutral diversity levels and their absence could affect parameter estimates and predictions. One possibility is to ignore this problem. Another is to add substitutions in regions with missing data [21]. To choose among these strategies, we compare the maximum likelihood values and *R*^2^ statistics of diversity along the genome under the two approaches (relying on the same reasoning that we use in choosing the window size to estimate mutation rates). To incorporate missing substitutions, we randomly pick their positions in regions with missing data, with a number of substitutions per codon chosen based on the average over regions with the same annotation within 200 kb. The results suggest that inferences incorporating missing substitutions fit the data slightly better (Table S1).

Calculating the effects of linked selection**.** As detailed in the Results, we calculate the expected diversity levels along the genome given a set of selection parameters in two steps. First, we evaluate the functions *b*(*x*| *t_g_*, *i_B_*) and *s*(*x*| *s_k_*, *i_S_*) at each position *x* in the genome, for each selection coefficient on the predefined grid (*g*=1, …, *G* and *k*=1,…, *K*) and selected annotations (*i_B_*=1,...,*I_B_* and *i_S_*=1,...,*I_S_*). These functions are pre-calculated and then used to obtain the expected diversity levels given a set of selection parameters (as specified in Equations (6) and (7)). Here, we specify how we calculate the functions *b*(*x*| *t_g_*, *i_B_*) and *s*(*x*| *s_k_*, *i_S_*).

**A. Goodness-of-fit measures.**

| Model | Background selection  and classic sweeps  with added "missing substitutions" | Background selection  and classic sweeps  without "missing substitutions" |
| --- | --- | --- |
| Δ*CL* | 3.9⨯10^-4^ | 3.8⨯10^-4^ |
| *R*^2^ 1 Mb | 0.71 | 0.70 |
| 100 kb | 0.44 | 0.44 |
| 10 kb | 0.26 | 0.25 |
| 1 kb | 0.20 | 0.20 |

**B. Parameter estimates.**

| Model | Background selection  and classic sweeps  with added "missing substitutions" | | | | Background selection  and classic sweeps  without "missing substitutions" | | | |
| --- | --- | --- | --- | --- | --- | --- | --- | --- |
|  | 73% | | | | 74% | | | |
| Annotation | Exons | UTRs | Introns | Intergenic | Exons | UTRs | Introns | Intergenic |
| Background selection parameters |  |  |  |  |  |  |  |  |
| *u*(*t*=10^-1.5^) / *μ* | 377% | 577% | 19% | - | 273% | 695% | 55% | 2% |
| *u*(*t*=10^-2.5^) / *μ* | 2% | 2% | - | - | 1% | 4% | - | - |
| *u*(*t*=10^-3.5^) / *μ* | 56% | - | - | - | 75% | - | - | - |
| *u*(*t*=10^-4.5^) / *μ* | 2% | 23% | - | - | - | 6% | 1% | - |
| *u*(*t*=10^-5.5^) / *μ* | - | 2% | - | - | - | 1% | - | - |
| Classic sweeps parameters |  |  |  |  |  |  |  |  |
| *α*(*s*=10^-1.5^) | - | - | - | - | - | - | - | - |
| *α*(*s*=10^-2.5^) | 0.6% | - | - | - | 1.1% | - | - | - |
| *α*(*s*=10^-3.5^) | 3.5% | - | - | - | 2.0% | - | - | - |
| *α*(*s*=10^-4.5^) | - | 5.1% | - | - | 3.8% | 6.8% | - | - |
| *α*(*s*=10^-5.5^) | 36.3% | 42.1% | - | - | 36.5% | 34.5% | - | - |

**Table S1. Results with and without adding missing substitutions.** Goodness-of-fit measures (**A**) and parameter estimates (**B**) obtained using the joint model with background selection and classic sweeps. In (**A**), *ΔCL* is the difference between the model's likelihood and the likelihood of a neutral model. *R*^2^ is measured in non-overlapping windows of 1Mb, 100kb, 10kb and 1kb. In (**B**): is the average estimated reduction in diversity; the fraction of deleterious mutations with a given selection coefficient is measured relative to our estimate for the overall mutation rate *μ*=6.8⨯10^-9^ per bp (see Section E); *α* denotes the fraction of substitutions driven by sweeps with a given selection coefficient.

Our calculation of *b*(*x*| *t_g_*, *i_B_*) follows McVicker et al. (2009) [22] and relies on their code. In brief, they consider a grid of selected segment lengths and a grid of genetic distances from the segment, and calculate *b* values over these grids using Equation (2) [23]. For a grid of positions along the genome, they then calculate the *b* value by summing over the effects of conserved segments, where the effect of a given segment is calculated based on bi-linear interpolation, using the grids over segment lengths and distances from the previous step. The spacing of grid positions along the genome is determined based on the first two derivatives of previous points, in order to ensure that changes in *b* values between consecutive points are not too large. Finally, *b* values between grid points are calculated based on linear interpolation. See McVicker et al. (2009) for further details.

The calculation of *s*(*x*| *s_k_*, *i_S_*) is similar in spirit but simpler, because it depends on the discrete positions of substitutions and we have a closed form description of the effects of a sweep as a function of the selection coefficient and genetic distance (Equation 3). For a given selection coefficient *s_k_*, we calculate *s*(*x*| *s_k_*, *i_S_*) for a grid of positions along the genome, where grid points are spaced according to the maximum of 3.3⨯10^-6^ and 1·*s_k_* cM. This spacing is chosen because 3.3⨯10^‑6^ cM corresponds to the average genetic length of a single codon and the effect of a classic sweep with selection coefficient *s_k_* extends over a distance of 10·*s_k_* cM. At each grid point, we sum over the effects of substitutions within a genetic distance of 100·*s_k_* cM given by Equation 3. Classic sweeps beyond this distance have a negligible effect on diversity levels [24]. The value of *s*(*x*| *s_k_*, *i_S_*) between grid points is calculated by linear extrapolation between values at flanking grid points.

Likelihood maximization*.* We maximize the likelihood function using a combination of optimization algorithms. We begin with each of the weights corresponding to the grid of selection coefficients set to a small positive value, amounting to a negligible deviation from neutrality, and with *π_0_* set to the observed genome-wide average. We then run three consecutive programs from the Matlab Optimization Toolbox: Active-Set, Interior-Point and Sequential-Quadratic-Programming [25]. We use the estimates obtained from the previous program as initial conditions for the next one, unless the run fails (either because the likelihood does not improve or because the exit flags reported by the output indicated an unsuccessful run). We consider the maximization successful if at least one of the programs completed successfully and, if this is the case, we use the parameter estimates obtained by the last successful one.

This algorithm was developed to assure that our parameter estimates are close to the maximum likelihood estimate (MLE) despite of the high dimensionality of the parameter space (with up to 78 parameters for the most complex model). We check the reliability of the algorithm in three sets of analyses:

**1. Choosing the sequence of optimization algorithms.** While using a single-algorithm or a different sequence of algorithms does not always lead to a successful maximization, the chosen sequence is successful for all the models described in the text. Moreover, in almost all cases all three algorithms complete successfully. Specifically, we confirm that this is the best of the six possible orderings of algorithms for the three main models: (i) background selection with four annotations, classic sweeps with three annotations, and a grid of five selection coefficients for each mode and annotation, (ii) the same for background selection alone, and (iii) the same for classic sweeps alone.

**2. More complex models lead to greater likelihood.** One indication that the algorithm converges to the MLE is that, for nested models, the maximum likelihood is always greater (or equal) for the more complex model. We confirm that this is the case using the following set of nested models: (i) the full model of background selection with four annotations and classic sweeps with three annotations, with 11 point masses; (ii) the same model with 5 point masses; (iii) the models for background selection and sweeps alone with 5 point masses; and (iv) the models with background selection and sweeps alone with 5 point masses and a single (exonic) annotation.

**3. Convergence to the same MLE from different initial conditions.** Another indication that the algorithm converges to the MLE is that it arrives at very similar parameter estimates based on a variety of initial conditions. While an exhaustive examination of initial conditions is infeasible, we run the inference using a series of initial conditions that should sample very different parts of the parameter space. We do so for the most complex model, including background selection with four annotations, classic sweeps with three annotations and a grid of 11 point masses for each annotation. The initial conditions that we examine include (i) no selection; (ii) strong selection (leading to a substantial reduction in diversity due to linked selection) with equal weights on selection coefficients for all masses and all annotations; and (iii) estimates inferred for simpler models, including a similar model but with 5 point masses and a similar model with background selection and sweeps alone and 5 point masses. From almost all initial conditions, the algorithm converges to very similar parameter estimates, as well as likelihoods and goodness of fit measures (i.e., *R*^2^ in windows of 1 kb to 1 Mb; see Section C). The sole exception is when we begin with no selection, i.e. with null values for all weights, in which the maximization leads to a sub-optimal solution, presumably because the algorithms encounter difficulty starting the search from initial conditions on the boundary of the parameter space. When we modify the weights slightly to positive but tiny values that do not cause any appreciable effect of linked selection, the maximization converges to the same estimates obtained using the other initial conditions. Because this form of 'no selection' condition is the most conservative with respect to the effects of linked selection, we choose it as the standard initial condition for the maximization procedure.

# C. Statistical analyses

Details about summaries and figures.

**Comparing predicted and observed diversity levels in windows (Fig 2 in the main text)***.* Each of the major autosomes (2 and 3) is divided into non-overlapping windows of 1 Mb, 100 kb, 10 kb or 1 kb. Observed and predicted average, scaled heterozygosity are then calculated for each window with data at more than 50 codons. Specifically, we calculate the observed levels by dividing the average synonymous heterozygosity per codon by the average number of synonymous substitutions per codon (see Section B). Predicted average levels are calculated using our predictions at the same codons. We calculate the *R*^2^ for the model in which observed scaled diversity levels equal their predicted values (y=x) across windows, where the weight for each window equals the number of codons in it.

**Average diversity levels around substitutions (Fig 3, 4 and 5 in the main text)**. We divide the genetic distance between the focal substitution and a maximal distance of 0.11 cM into 10^-6^ cM bins. For each bin, we calculate the observed scaled diversity level (as detailed above) using all the codons that are found at the specified distance from a substitution. Similarly, we obtain the levels predicted based on our method by averaging the predictions over the same set of codons. Predicted levels at each distance based on the Sattath et al. method are calculated directly from their coalescent model.

**Observed diversity levels as a function of predicted levels (Fig 6 in the main text)***.* For each model, we order codons according to the predicted diversity level and then divide them into bins with equal amounts of data. For the Wiehe-Kim-Stephan models, predicted levels are determined either by the local recombination rate or by the local density of non-synonymous substitutions; these are calculated in 0.03 cM non-overlapping windows along the genome and linearly interpolated between window midpoints. Observed and predicted diversity levels are then calculated for each bin, as described above. While we conduct this analysis using 25, 100, 400 and 1600 bins, we only show the graphs for 100 bins and the correlations for 1600 bins.

Leave-one-out cross-validation analysis. Because we use the same data to infer parameters and evaluate our predictions, over-fitting might inflate our goodness-of-fit estimates. We use a leave-one-out cross-validation (LOOCV) analysis [26] to assess the extent of this problem. Specifically: i) we divide the genome into non-overlapping windows of 1 Mb; ii) dropping each window in turn, we infer selection parameters using the rest of the genome, excluding codons in the window and at distance < 0.5 Mb from it (to avoid correlations between diversity levels at the edges); iii) we use the inferred selection parameters to predict diversity levels within that window; iv) by combining predictions over all windows, we derive a genome-wide map of predicted diversity levels, where the prediction at any site does not rely on polymorphism data at that site or anywhere in its vicinity. In Table S2, we compare our predictions on different spatial scales based on the entire dataset and on LOOCV. We do so for both the recombination threshold of 0.75 cM/Mb used in the inference and for the recombination threshold of 0.1 cM/Mb used in the main text (where polymorphism data for regions with recombination rate < 0.75 cM/Mb are not used in the inference). The results with LOOCV and the entire dataset are very similar, showing that over-fitting has little effect on our results.

|  | **BS & CS**  **( > 0.10 cM/Mb )** | | **BS & CS**  **( > 0.75 cM/Mb )** | |
| --- | --- | --- | --- | --- |
|  | **LOOCV** | **Full** | **LOOCV** | **Full** |
| **1 Mb** | 70% | 71% | 59% | 60% |
| **100 Mb** | 43% | 44% | 28% | 29% |
| **10 Kb** | 27% | 26% | 17% | 18% |
| **1 Kb** | 21% | 20% | 13% | 14% |

**Table S2. Leave-one-out cross-validation (LOOCV) analysis.**

Quantifying the relative contribution of background selection and sweeps**.** In the Discussion, we consider the relative effects of different modes of linked selection and specifically those of background selection and classic sweeps. One way to quantify these contributions relies on our model for expected heterozygosity. Notably, based on Equation 1 and measuring time in units of 2*N_e_* generations, the coalescence rates due to genetic drift equals 1 throughout the autosomes, the increase in rate at autosomal position *x* due to background selection is $B^{-1}(x)-1$ and the increase due to classic sweeps is $2N_{e}S(x)$. The genome-wide average of these rates $r_{B}=\overline{B^{-1}}-1$ and $r_{S}=2N_{e}\bar{S}$ therefore provide a natural additive measure for the effect of each mode, with the ratios $1:r_{B}:r_{S}$ quantifying the relative contribution of drift, background selection and classic sweeps and *r_B_*+*r_S_* quantifying the total contribution of linked selection.

Although these measures are natural in some respects, they also have some limitations. For instance, in the Discussion, we break up the contribution of background selection due to strong and moderate selection. We cannot do the same with the above measures because the effects of background selection due to different selection coefficients combine multiplicatively (we could, however, do so for sweeps). Also, quantifying the relative contributions of background selection and classic sweeps in terms of average coalescence rates could be misleading because the averages might be dominated by regions with high coalescence rates (e.g., with low recombination) in which diversity levels would be low even if only one mode of linked selection were present.

We therefore consider a second measure that quantifies the effects on diversity levels more directly. For that purpose, we rely on our parameter estimates to build maps predicting diversity levels due to classic sweeps or background selection alone, considering the parameter estimates derived for the joint model; by the same token, we can also build maps corresponding to background selection due to strong or moderate selection coefficients. Based on these maps, we calculate the average relative reduction in heterozygosity if only background selection were present, , or only classic sweeps, , or both, . Then we quantify the relative reduction due to background selection by $\frac{k_{B}}{k_{B}+k_{S}}$ and due to sweeps by $\frac{k_{S}}{k_{B}+k_{S}}$. Similar definitions are used for subsets of selection coefficients associated with background selection. These measures are used in the Results section, and in Table S3 we provide both kinds of measures for several models.

|  | Model | BS & CS | BS | CS | BS & CS | BS & CS | BS | BS & CS | BS |
| --- | --- | --- | --- | --- | --- | --- | --- | --- | --- |
|  |  |  |  |  | 11 masses | u_del_ constrained | u_del_ constrained | excluding UTR sweeps | Charlesworth |
| Diversity reduction measures |  | 73% | 65% | 43% | 86% | 59% | 49% | 74% | 37% |
|  | *k_B_* | 67% | 66% | - | 81% | 49% | 49% | 69% | 38% |
|  | *k_S_* | 41% | - | 43% | 69% | 32% | - | 37% | - |
|  | *k_B_*/(*k_B_*+*k_S_*) | 62% | 100% | - | 54% | 61% | 100% | 65% | 100% |
|  | *k_S_*/(*k_B_*+*k_S_*) | 38% | - | 100% | 46% | 39% | - | 35% | - |
| Coalescent rate measures | *r_B_*+*r_S_* | 3.18 | 2.13 | 0.87 | 7.59 | 1.66 | 1.07 | 3.28 | 5.23 |
|  | *r_B_* | 2.26 | 2.13 | - | 4.72 | 1.08 | 1.07 | 2.51 | 5.23 |
|  | *r_S_* | 0.92 | - | 0.87 | 2.87 | 0.58 | - | 0.77 | - |
|  | *r_B_*/(*r_B_*+*r_S_*) | 71% | 100% | - | 62% | 65% | 100% | 77% | 100% |
|  | *r_S_*/(*r_B_*+*r_S_*) | 29% | - | 100% | 38% | 35% | - | 23% | - |

**Table S3. The relative contribution of background selection and classic sweeps.** All genome-wide average coalescence rates are shown in units corresponding to genetic drift in the absence of linked selection.

# D. Interpreting inferences about classic sweeps

Our inference is predicated on a model of classic sweeps on semi-dominant alleles, which takes the form of an exponential decay of coalescence rates around beneficial substitutions, where the rate of decay depends on the selection coefficient. In the likelihood calculation, we model coalescence rates around observed substitutions as a superposition of such exponentials, with weights that follow from the distribution of selection coefficients. Other modes of linked positive selection have similar effects on the expected coalescence rates around substitutions. Below, we discuss how this similarity allows us to interpret our estimates.

Partial sweeps**.** We rely on the work of Coop and Ralph [27] to illustrate the equivalence between the expected effect of partial sweeps, in which a new mutation rises quickly to intermediate frequency but fixes less rapidly [28,29], and of a mixture of classic sweeps (Fig S2). Consider a model with two types of classic sweeps, one driven by stronger selection than the other, and assume that strong selection drives a fraction *α*_1_ of substitutions to fixation over *t*_1_ generations and weaker selection a fraction *α*_2_ to fixation over *t*_2_ generations (*t*_2_>> *t*_1_). Next consider a model in which a fraction *α* of substitutions are driven to fixation by a single kind of partial sweeps, where a new mutation is driven to frequency *x* over *t_P_* generations and then to fixation over *t_F_* generations, and *t_F_*>>*t_P_*, reflecting much stronger selection in the initial stage. At neutral sites close to a substitution (*r*<<1/*t_F_*), the expected coalescent rate is *α*∙*Exp*(-*r*(*t_P_*+*t_F_*)), and at sites farther away (*r*>>1/*t_F_*), the expected rate is *α*∙*x*^2^∙*Exp*(-*r*∙*t_P_*) [27]. It follows that the two models would generate similar expected diversity levels around substitutions if their parameters satisfy the requirements that *α*_1_=*α*∙*x*^2^ and *α*_2_=*α*∙(1-*x*^2^), as well as *t*_1_=*t_P_* and *t*_2_=*t_F_*+*t_P_*. More generally, there is a continuous spectrum of mixtures of partial and classic sweeps that would generate similar diversity patterns. Moreover, this equivalence can be generalized to partial sweeps with more than two different selected phases.


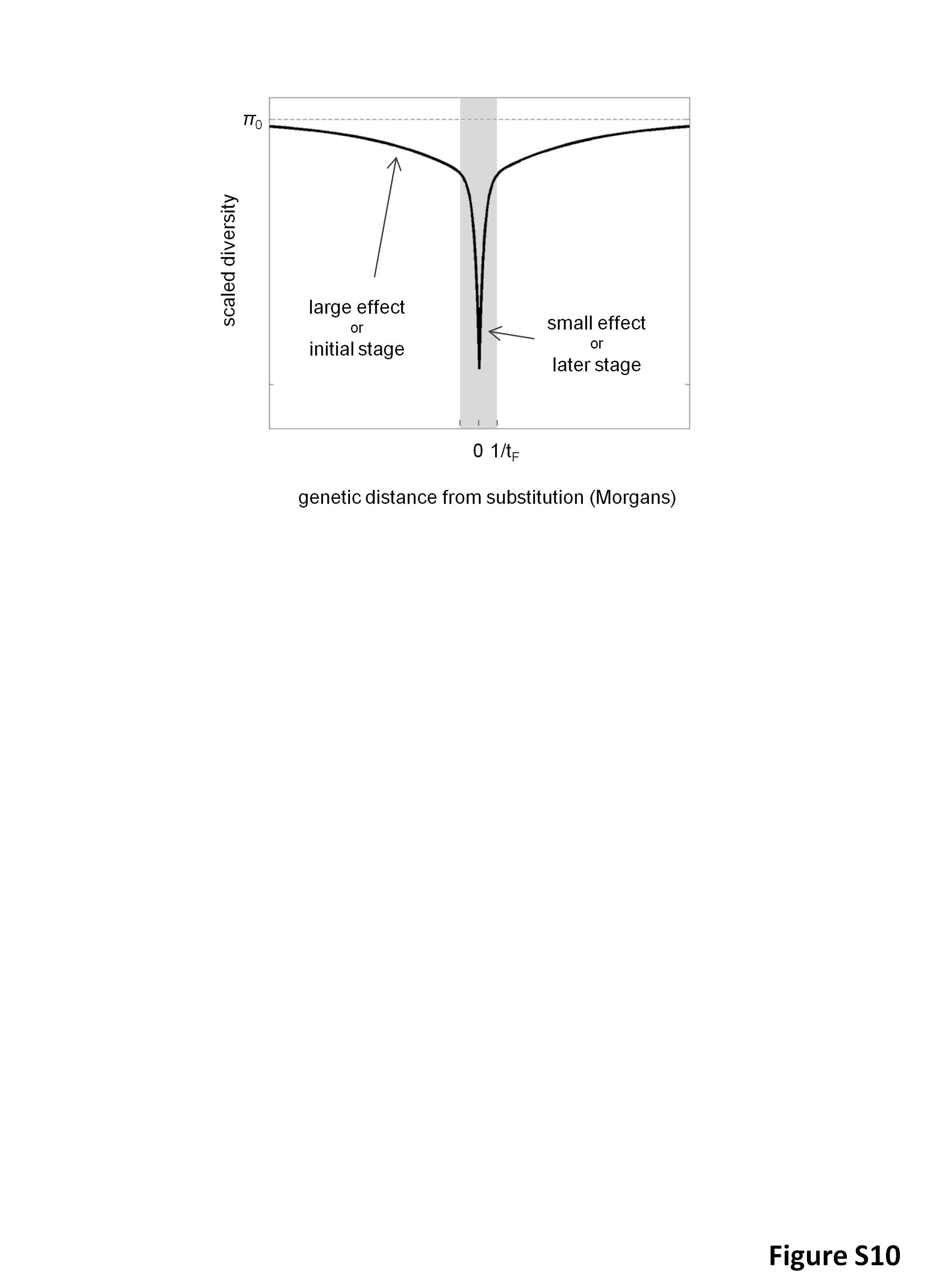


**Fig S2.** **Similarities between signatures of classic and partial sweeps**. The figure illustrates the similarity between expected diversity levels under a mixture of classic sweeps driven by either weak or strong selection and a single kind of partial sweeps initially driven by strong selection to intermediate frequency and then to fixation by weak selection. The strong classic sweeps or the initial stage of the partial sweeps govern diversity levels far from substitutions while the weak classic sweeps or the final stage of the partial sweeps govern them close to the substitution.

This equivalence suggests how to interpret our parameter estimates in the presence of a mixture of classic and partial sweeps. Namely, the total estimate of the fraction of substitutions driven by positive selection remains the same, but in this case, the fraction associated with a specific selection coefficient reflects both the fraction of beneficial substitutions driven by such selection sometime during their trajectory and the part of the trajectory in which it acts. More precisely, this estimate corresponds to either the fraction of full trajectories with the same selection coefficient, i.e. classic sweeps, or to a greater fraction of partial trajectories with equivalent effects on diversity.

Soft sweeps (multiple mutations). We can make a similar argument for soft sweeps, where multiple beneficial alleles at the same locus sweep to intermediate frequencies in response to the same selection pressure [30]. During such a soft sweep, none of the beneficial alleles fix and thus no substitution occurs. Over a longer period, however, one of these alleles will fix due to drift or selection. If it is due to drift then there will be sufficient time for recombination to reduce linkage disequilibrium around the selected site to background levels, such that this fixation period will not leave a signature of linked selection close to the selected site. If, instead, the beneficial alleles have similar, but not identical, selection coefficients then the most favorable allele will likely fix, but will do so under much weaker selection (depending on its selective advantage over the other beneficial alleles), resulting in a process akin to the slower phase of the partial sweep considered above.

The resulting effect on diversity levels can be approximated as follows. Assume that the initial soft sweep takes *t_S_* generations and that the subsequent fixation of the neutral or favored allele takes *t_F_* generations, where *t_F_*>>*t_S_*. Pennings and Hermisson [31] showed that, with a population-scaled beneficial mutation rate at the selected locus *θ_B_*, the probability that two lineages, sampled immediately after the initial soft sweep, coalesce during the sweep is (1/(1+*θ_B_*))*Exp*(-*r*∙*t_S_*). Following the same logic as above (i.e., that of Coop and Ralph [27]), after fixation, farther from the selected site (*r*>>1/*t_F_*), the expected rate of coalescence would be (1/(1+*θ_B_*))*Exp*(-*r*∙*t_S_*). In turn, the expected rate of coalescence closer to the selected site (*r*<<1/*t_F_*) depends on whether fixation occurs by drift or selection. If it is due to drift then the expected rate of coalescence would be the same as at a farther distance, i.e., (1/(1+*θ_B_*))*Exp*(-*r*∙*t_S_*), but if it is due to selection then lineages would be affected by the entirety of the sweep and the expected rate of coalescence would be *Exp*(‑*r*(*t_S_*+*t_F_*)) (where we assume that 1/(1+*θ_B_*)>>*Exp*(-*r*∙*t_F_*)).

These approximations suggest how our estimates of sweep parameters could be interpreted in the presence of soft sweeps. A fraction *α* of substitutions due to soft sweeps with a neutral fixation phase would appear in our estimates as *α*/(1+*θ_B_*) sweeps driven by the selection coefficient characterizing the initial soft sweep phase. Thus, having multiple beneficial mutations would cause the fraction that we infer to be an underestimate of the fraction of beneficial substitutions, while our estimate of the selection coefficient would be unbiased. If, instead, the fixation phase is also driven by selection, then we would estimate that a fraction of (approximately) *α* substitutions were driven by weak selection corresponding to a beneficial sojourn time of *t_S_*+*t_F_* and (approximately) a fraction *α*/(1+*θ_B_*) were driven by strong selection corresponding to a beneficial sojourn time of *t_S_*. This case is similar to the case of partial sweeps outlined above, with *α*/(1+*θ_B_*) replacing *α*∙*x*^2^.

Sweeps from standing variation**.** Another kind of sweep occurs when the selected allele that fixes was common when selection began [32-35]. Specifically, consider an allele that was present in the population at frequency *f* (1/2*N_e_*<<*f*<<1) at the onset of selection, either having drifted to that frequency or having been balanced close to that frequency for some (but not a very long) time. Selection then begins to favor the allele (in a semi-dominant manner) and it sweeps to fixation over *t_S_* generations. For a pair of lineages at genetic distance *r* from the selected allele, the probability of coalescence due to the sweep is then well approximated by
1/(1+4*N_e_r*∙*f*(1-*f*))*Exp*(-*r*∙*t_S_*) [36]. Thus, far from the selected site, where the distance is defined by the initial frequency, i.e., 4*N_e_r*∙*f*>>1, the sweep will have little effect on diversity levels. Close to the selected site, i.e., when 4*N_e_r*∙*f*<<1, the probability of coalescence can be further approximated by *Exp*(-*r*(*t_S_*+4*N_e_f*(1-*f*))).

The interpretation of our estimates in the presence of sweeps from standing variation follows. If the initial frequencies of beneficial alleles are large then the sweeps will not be captured by our inference because they have negligible effect on diversity levels. In other words, substitutions driven by such sweeps will be estimated as neutral. For substitutions driven by sweeps with sufficiently small initial frequencies, we will estimate their fraction correctly but the selection coefficients will be downwardly biased, corresponding to a beneficial sojourn time of approximately *t_S_*+4*N_e_f*(1-*f*). This sojourn time is in fact roughly the coalescent time of two selected alleles in the population but it is greater than the classic sweep equivalent with the same selection coefficient. Overall, for substitutions caused by sweeps from standing variation, we will underestimate the selection coefficients and their fraction.

Recessive and other classic sweeps**.** We could also have classic sweeps caused by beneficial alleles that are recessive, or more generally, not semi-dominant [37,38]. In that case, a recessive sweep would leave a signature that is similar to a sweep from standing variation. Given that we are considering sweeps that lead to substitutions, we know that the selected allele will start from a single mutation and proceed all the way to fixation. Until the beneficial allele reaches a sufficiently high frequency, i.e., while 2*N_e_s*∙*f*^2^<<1, its dynamics would be governed by genetic drift. The frequency at which selection will kick in can be approximated by $f\approx1/\sqrt{2N_{e}s}$. The duration of the selected phase, *t_S_*, can then be calculated using the diffusion approximation [39]. The interpretation of our estimates is then the same as for the case of sweeps from standing variation with these parameters. In turn, for beneficial alleles with dominance coefficients other than 0 or ½, the mapping to the inferred semi-dominant selection coefficient is provided by the sojourn time for the beneficial allele, again given by the diffusion approximation.

A mixture including different kinds of sweeps***.*** In reality, we would expect a mixture of a variety of sweeps to occur, raising the question about the best way to interpret our estimates. While we discussed how various kinds of sweeps could “bias” our estimates, and in principle, these “biases” could be considered together, we believe this would not be a very productive interpretation. For example, it is not obvious whether, with soft sweeps in the mix, our estimate for the fraction of substitutions driven by a given selection coefficient, *α*(*s*), is best interpreted as an underestimate of the fraction of substitutions driven by such selection during some part of their trajectory. Instead, since we are interested in the effects on diversity level, we consider our estimate to be a summary of the effect of a continuous variety of partial sweeps, in which different parts of the adaptive trajectory are driven by this selection coefficient—a classic sweep equivalent that summarizes over a continuous set of possibilities. More generally, because the various kinds of sweeps result in similar functional forms, we may interpret our estimates as designating a class of mixtures that would result in similar expected diversity levels around substitutions.

In principle, the kind of derivations that we outlined could be used to write down equations for the possible mixtures. However, if we were to use all the kinds of sweeps mentioned above then we would end up with equations including numerous parameters, without, as of now, a way to solve for them. Even when methods are developed to use other aspects of the data to learn about some of these parameters, we will no doubt need some kind of coarse-graining scheme in order to think about these mixtures. Both because there are more parameters than we are likely to be able to infer with any certainty, and because, to begin with, we would have to have some coarse graining for the continuous ranges of many of these parameters (e.g., selection and dominance coefficients and initial frequencies for different sweep phases). Moreover, while we outlined how the main kinds of sweeps considered to date would be recorded in our estimates, there is actually a continuous range of possibilities. For example, there are hybrids of the kinds that we have considered, such as a sweep that begins from standing variation but then proceeds through a selected phase divided into several phases with different selection coefficients (in fact, such sweeps are expected under models of polygenic adaptation [28,40]). Given these considerations, we do not try to dissect the different mixtures of sweeps any further.

# E. Interpreting the inferences about background selection

Imposing an upper bound on the mutation rate*.* The unreasonably high estimates obtained for the deleterious mutation rate lead us to consider models in which we impose a biologically plausible upper bound. As an upper bound, we use an estimate for the total mutation rate in *D. melanogaster*, which we arrive at as follows. For point mutations, we use a rate of 3.5×10^-9^ per bp per generation, which was estimated by Keightley et al. based on mutation accumulation experiments [41]. For indels, we use a rate of 1.6×10^-9^ per bp per generation, which derives from estimates of the ratio of indel to point mutation rates (8.4×10^-9^/5.8×10^-9^) taken from Haag-Liautard et al. [42] and the aforementioned estimate for point mutations (while Haag-Liautard et al. also estimated the point mutation rate, they did so based on considerably less data). Additionally, we use a rate of transposable element (TE) insertions of 1.7×10^-9^ per bp per generation, based on Nuzhdin & Mackay’s [43] estimate of 0.2 TE insertions per genome per generation and assuming a uniform rate along the genome. Taken together these rates sum up to *μ*=6.8×10^-9^ mutations per bp per generation, which we use as an upper bound on the deleterious mutation rate. The rate of TE insertions might be somewhat higher [44] and the estimates based on mutation accumulation experiments might be noisy due to variation among lines [41,45]. However, moderate changes in the bound would not change the conclusions of our analysis.

We find that imposing an upper bound on the deleterious mutation rate introduces artifacts into the inference rather than making our estimates more plausible. The new estimates have a reduced genomic rate of deleterious mutations associated with strong selection (*t*=10^-1.5^), and the bulk of these mutations are shifted from exons and UTRs to introns and intergenic regions (Table S4). Taken at face value, these new estimates do not make much sense because they imply a similar proportion of sites under strong purifying selection in exons and long introns, which clearly contradicts findings based on sequence conservation and diversity patterns [9-11]. Moreover, even if we assume that these estimates absorb the effects of other forms of linked selection, it would still make little sense for such effects to be similar (let alone greater) in long introns compared to exons and UTRs [9]. Instead, the new estimates appear to be an artifact (Fig S3). Because we cap the deleterious mutation rate, the model requires additional sites under strong purifying selection; and because strong selection (*t*=10^-1.5^) affects diversity level in a non-localized fashion, assigning the strongly deleterious mutations to annotations other than exons and UTRs has a minor effect on the fit. These considerations suggest that the inference based on the unconstrained model better reflects the effects of linked selection. If so, the unrealistically high estimates of the rate of deleterious mutations around coding regions are likely absorbing other modes of linked selection, which are also most likely to be acting on these regions.

Our uncertainty about π_0_**.** We find that the constrained and unconstrained models yield very similar fits, as measured by the log composite likelihood (where *ΔCL* is its increase compared to a neutral model) and a variety of summaries (Table S4), while their estimates for the diversity levels in the absence of linked selection, *π*_0_, are substantially different. As noted, this difference corresponds to different estimates for the deleterious mutation rate associated with strong selection (Fig S3) and is possible because the data carries little direct information about *π*_0_. As we note in the manuscript, this introduces considerable uncertainty regarding the overall reduction in diversity levels caused by linked selection (Table S4).


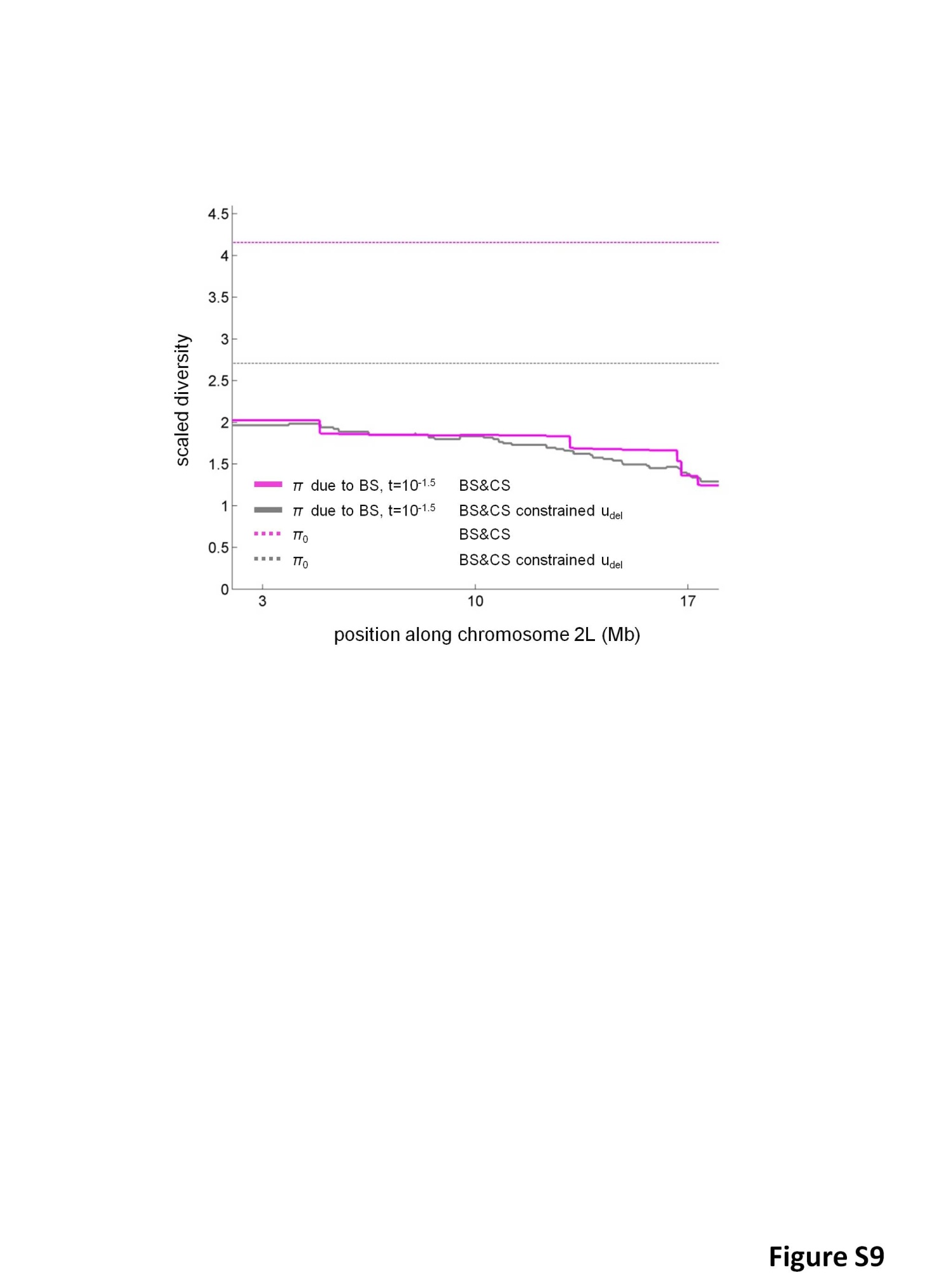


**Fig S3. The predicted effects of background selection due to strongly deleterious mutations (*t*=10^-1.5^) in the constrained and unconstrained models**. Shown are the predicted scaled diversity levels based on the two models, when considering only the effects of linked selection caused by strongly deleterious mutations. See Table S4 for the parameter estimates for these models.

|  | Model | Background selection and classic sweeps | | | | Background selection and classic sweeps with constrained *u*_del_ | | | |
| --- | --- | --- | --- | --- | --- | --- | --- | --- | --- |
| Parameters | $\pi_{0}/\bar{\pi}$ | 4.4 | | | | 2.8 | | | |
|  | *U*_del_ (per diploid) | 1.60 | | | | 0.92 | | | |
|  | Annotation | Exons | UTRs | Introns | Intergenic | Exons | UTRs | Introns | Intergenic |
|  | *u*_del_ / *μ* | 437% | 603% | 19% | - | 90% | 88% | 87% | 44% |
|  | *u*(*t*=10^-1.5^) / *μ* | 377% | 577% | 19% | - | 18% | 67% | 85% | 44% |
|  | *u*(*t*=10^-2.5^) / *μ* | 2% | 2% | - | - | 2% | 2% | 1% | - |
|  | *u*(*t*=10^-3.5^) / *μ* | 56% | - | - | - | 69% | - | - | - |
|  | *u*(*t*=10^-4.5^) / *μ* | 2% | 23% | - | - | 1% | 20% | - | - |
|  | *u*(*t*=10^-5.5^) / *μ* | - | 2% | - | - | - | - | - | - |
|  | $\sum_{s} \alpha(s)\cdot s$ | 3.2⨯10^-5^ | 3.0⨯10^-6^ | - | - | 3.0⨯10^-5^ | 3.0⨯10^-6^ | - | - |
|  | *α* | 40% | 47% | - | - | 42% | 51% | - | - |
|  | *α*(*s*=10^-1.5^) | - | - | - | - | - | - | - | - |
|  | *α*(*s*=10^-2.5^) | 0.6% | - | - | - | 0.6% | - | - | - |
|  | *α*(*s*=10^-3.5^) | 3.5% | - | - | - | 3.5% | - | - | - |
|  | *α*(*s*=10^-4.5^) | - | 5.1% | - | - | - | 4.7% | - | - |
|  | *α*(*s*=10^-5.5^) | 36.3% | 42.1% | - | - | 38.1% | 45.9% | - | - |
|  | *ΔCL* | 3.9⨯10^-4^ | | | | 3.6⨯10^-4^ | | | |
| Diversity binned in local windows | *R*^2^ 1 Mb | 0.71 | | | | 0.69 | | | |
|  | 100 kb | 0.44 | | | | 0.43 | | | |
|  | 10 kb | 0.26 | | | | 0.24 | | | |
|  | 1 kb | 0.20 | | | | 0.19 | | | |
| Diversity binned by distance from substitution | *R*^2^ NS  substitutions | 0.62 | | | | 0.61 | | | |
|  | SYN  substitutions | 0.66 | | | | 0.69 | | | |
| Diversity binned by predicted effect of linked selection | Spearman's *ρ* | 0.913 | | | | 0.905 | | | |
|  | Upper-to-lower tails   observed diversity   ratio | 5.3 | | | | 5.4 | | | |
| Diversity reduction measures |  | 73% | | | | 59% | | | |
|  | *k_B_* | 67% | | | | 49% | | | |
|  | *k_S_* | 41% | | | | 32% | | | |
|  | *k_B_*/(*k_B_*+*k_S_*) | 62% | | | | 61% | | | |
|  | *k_S_*/(*k_B_*+*k_S_*) | 38% | | | | 39% | | | |
| Coalescent rate measures | *r_B_*+*r_S_* | 3.18 | | | | 1.66 | | | |
|  | *r_B_* | 2.26 | | | | 1.08 | | | |
|  | *r_S_* | 0.92 | | | | 0.58 | | | |
|  | *r_B_*/(*r_B_*+*r_S_*) | 71% | | | | 65% | | | |
|  | *r_S_*/(*r_B_*+*r_S_*) | 29% | | | | 35% | | | |

**Table S4. Parameter estimates, goodness-of-fit and other summaries for models constraining *u_del_* or not.**

# ***F. Comparison with maps based on the Charlesworth approach***

Pioneering work by Charlesworth [44,46] used estimates of the rates and distributions of selection coefficients of deleterious mutations and genetic maps, to demonstrate that background selection could account for the large-scale changes in diversity levels along chromosomes of *D.* *melanogaster*. More recently, this approach was extended by Comeron [47] to incorporate the spatial distributions of constrained genomic regions. This approach differs from ours in several ways, most notably in that estimates of selection parameters are not based on the effects of linked selection. Instead, estimates of the total rate of deleterious mutations come from mutation accumulation lines and estimates of the rate and distribution of selection coefficients at coding and non-coding annotations stem primarily from the direct effects of purifying selection on divergence and on the site frequency spectrum. Here we compare the predictions of this method with ours.

To this end, we introduce several modifications. At exonic regions, Charlesworth used a gamma distribution of selection coefficients truncated at *t*=5×10^-6^. We use a discretized approximation of this distribution on the grid *t*=10^-1.5^, 10^-2.5^, 10^-3.5^, 10^-4.5^, 10^-5.5^ (integrating between mid points on the log-linear scale). For non-exonic regions, Charlesworth assumed that short segments switch between being under strong and weak selection, where the distribution of selection coefficients in each type of segment is also described by a gamma distribution truncated at *t*=5×10^-6^. We use a discretized approximation of the mixture of the two distributions, weighted by the relative length of segments; because the alternating segments are short, assuming a homogeneous mixture has a negligible effect on predicted diversity levels. The resulting selection parameters are shown in Table S5, alongside our estimates. Similar to Comeron, we use the spatial distribution of exonic and non-exonic regions along the genome. Finally, to determine the expected diversity level in the absence of background selection, *π*_0_, we require that the mean diversity level predicted by the model matches the observed one.

Fig S4 shows a comparison between the predictions of the two methods and observed diversity levels. While the predictions along the chromosome appear similar, a quantitative comparison indicates that our method does better at all spatial scales (Fig S4A and Table S5). Both visual and quantitative comparison based on diversity patterns around substitutions (Fig S4B and Table S5) and on the stratification of diversity levels (Fig S4C and Table S5) confirms that our method performs better.

The fact that our method provides a better prediction of diversity levels is not surprising, given that our inference relies on observed diversity levels. Our leave-one-out cross validation analysis of *R*^2^ values on different spatial scales, however, suggests that over-fitting explains a negligible part of the difference (Section C). Instead, we believe that two other factors are more important. First, as we noted in the manuscript, our inferences for background selection likely absorb the effects of other modes of linked selection and accounting for (some) of these effects lends better predictive abilities. Second, Charlesworth’s and Comeron's estimates for the distribution of selection coefficients rely primarily on signatures of direct (in contrast to linked) purifying selection, which, provided the population sample sizes currently available for *D. melanogaster*, only allows one to probe a relatively narrow range of selection coefficients (cf. [48]). Using spatial diversity patterns likely provides insight into a wider range of selection coefficients.

|  | Model | Background selection and classic sweeps | | | | Background selection alone | | | | Background selection based on Charlesworth | | | |
| --- | --- | --- | --- | --- | --- | --- | --- | --- | --- | --- | --- | --- | --- |
|  |  |  | | | |  | | | |  | | | |
| Parameters | *U*_del_ (per diploid) | 1.60 | | |  | 1.46 | | | | 0.56 | | | |
|  | Annotation | Exons | UTRs | Introns | Intergenic | Exons | UTRs | Introns | Intergenic | Exons | UTRs | Introns | Intergenic |
|  | *u*_del_ / *μ* | 437% | 603% | 19% | - | 448% | 456% | 17% | - | 72% | 38% | 38% | 38% |
|  | *u*(*t*=10^-1.5^) / *μ* | 377% | 577% | 19% | - | 369% | 453% | 17% | - | - | - | - | - |
|  | *u*(*t*=10^-2.5^) / *μ* | 2% | 2% | - | - | - | - | - | - | 25% | 7% | 7% | 7% |
|  | *u*(*t*=10^-3.5^) / *μ* | 56% | - | - | - | 77% | - | - | - | 27% | 8% | 8% | 8% |
|  | *u*(*t*=10^-4.5^) / *μ* | 2% | 23% | - | - | 2% | 3% | - | - | 15% | 10% | 10% | 10% |
|  | *u*(*t*=10^-5.5^) / *μ* | - | 2% | - | - | - | - | - | - | 5% | 12% | 12% | 12% |
|  | *ΔCL* | 3.9⨯10^-4^ | | | | 2.8⨯10^-4^ | | | | -6.7⨯10^-5^ | | | |
| Diversity binned in local windows | *R*^2^ 1 Mb | 0.71 | | | | 0.76 | | | | 0.58 | | | |
|  | 100 kb | 0.44 | | | | 0.42 | | | | 0.19 | | | |
|  | 10 kb | 0.26 | | | | 0.23 | | | | 0.09 | | | |
|  | 1 kb | 0.20 | | | | 0.18 | | | | 0.08 | | | |
| Diversity binned by distance from substitution | *R*^2^ NS  substitutions | 0.62 | | | | 0.27 | | | | - | | | |
|  | SYN  substitutions | 0.66 | | | | 0.53 | | | | 0.05 | | | |
| Diversity binned by predicted effect of linked selection | Spearman's *ρ* | 0.913 | | | | 0.745 | | | | 0.773 | | | |
|  | Upper-to-lower   tails observed  diversity ratio | 5.3 | | | | 4.4 | | | | 3.5 | | | |
| Diversity reduction measures |  | 73% | | | | 66% | | | | 38% | | | |
|  | *k_B_* | 67% | | | | 66% | | | | 38% | | | |
|  | *k_S_* | 41% | | | | - | | | | - | | | |
|  | *k_B_*/(*k_B_*+*k_S_*) | 62% | | | | 100% | | | | 100% | | | |
|  | *k_S_*/(*k_B_*+*k_S_*) | 38% | | | | - | | | | - | | | |
| Coalescent rate measures | *r_B_*+*r_S_* | 3.18 | | | | 2.13 | | | | 5.23 | | | |
|  | *r_B_* | 2.26 | | | | 2.13 | | | | 5.23 | | | |
|  | *r_S_* | 0.92 | | | | - | | | | - | | | |
|  | *r_B_*/(*r_B_*+*r_S_*) | 71% | | | | 100% | | | | 100% | | | |
|  | *r_S_*/(*r_B_*+*r_S_*) | 29% | | | | - | | | | - | | | |

**Table S5. Comparison of the method of Charlesworth and ours.**

**
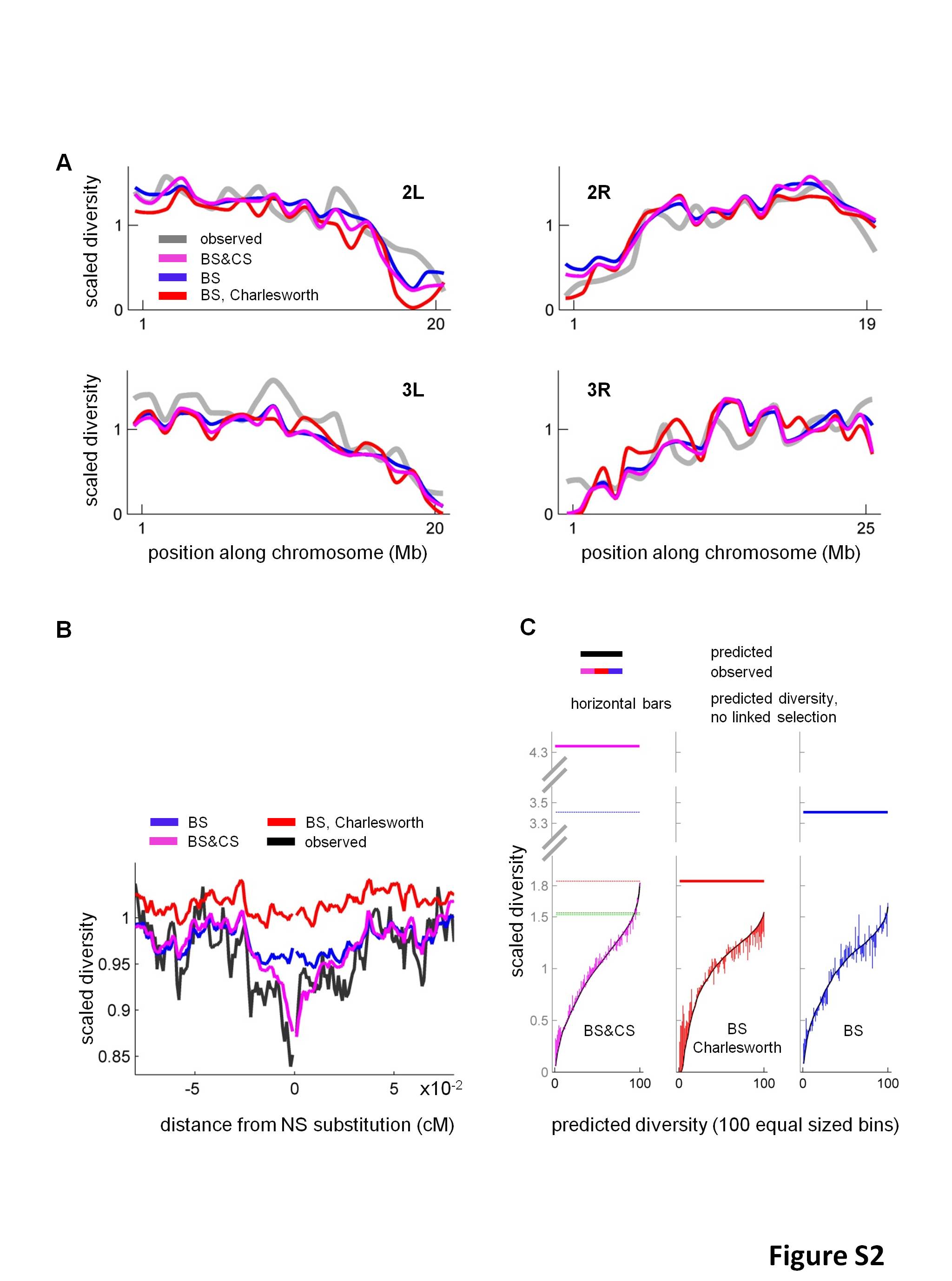
**

**Fig S4**. **Predicted scaled diversity levels based on the method of Charlesworth and ours.** (**A**) Diversity levels along the major autosomes. Other details are as in Fig 2A in the main text. (**B**) Diversity levels around non-synonymous substitutions. Other details are as in Fig 3B in the main text. (**C**) The impact of linked selection on diversity levels. Other details are as in Fig 6A in the main text. See Table S5 for the corresponding summaries.

# G. Comparison to other inference methods

To compare our results with those of other methods used to infer selection parameters based on signatures of linked selection (e.g., Fig 3 and 6 in the main text), we proceed as follows.

Inferring sweep parameters based on the Sattath et al. method. We apply the Sattath et al. [21] method to estimate classic sweep parameters associated with amino acid substitutions. The method relies on finding the parameters that provide the best fit to the observed average scaled synonymous diversity as a function of distance from amino acid substitutions. We rely on the same synonymous diversity and divergence data used for the current method, including a recombination threshold of 0.75 cM/Mb or greater. We collate synonymous diversity levels and numbers of substitutions per codon as detailed in Section C, up to a distance of 0.11 cM from substitutions, using bins of 10^-6^ cM. We then apply the composite likelihood maximization described in Sattath et al. (2011) using a parametric distribution of selection coefficients consisting of three point masses (in addition to *s*=0), where in this case the values of the point masses are also parameters that are free to vary (i.e., there are six free parameters overall). In practice, only two of the point masses appear in the MLE (see "CS, Sattath et al." in Table S6).

|  | Model | Background selection  and classic sweeps | | | Classic sweeps | | | Classic sweeps based on Sattath et al. | |
| --- | --- | --- | --- | --- | --- | --- | --- | --- | --- |
| Parameters | Annotation | Exons | UTRs | Introns | Exons | UTRs | Introns | Exons |  |
|  | $\sum_{s} \alpha(s)\cdot s$ | 3.2⨯10^-5^ | 3.0⨯10^-6^ | . | 1.1⨯10^-4^ | 3.6⨯10^-5^ | . | 6.1⨯10^-5^ |  |
|  | *α* | 40% | 47% | . | 40% | 51% | . | 20% |  |
|  | *α*(*s*=10^-1.5^) | . | . | . | 0.3% | 0.1% | . |  |  |
|  | *α*(*s*=10^-2.5^) | 0.6% | . | . | 0.4% | . | . | 1.5% | *s*=10^-2.4^ |
|  | *α*(*s*=10^-3.5^) | 3.5% | . | . | 3.7% | . | . |  |  |
|  | *α*(*s*=10^-4.5^) | . | 5.1% | . | . | 4.4% | . |  |  |
|  | *α*(*s*=10^-5.5^) | 36.3% | 42.1% | . | 35.7% | 46.1% | . | 18.8% | *s*=10^-5.2^ |
|  | *ΔCL* | 3.9⨯10^-4^ | | | 2.4⨯10^-4^ | | |  | |
| Diversity binned in local windows | *R*^2^ 1 Mb | 0.71 | | | 0.67 | | |  | |
|  | 100 kb | 0.44 | | | 0.39 | | |  | |
|  | 10 kb | 0.26 | | | 0.21 | | |  | |
|  | 1 kb | 0.20 | | | 0.16 | | |  | |
| Diversity binned by distance from substitution | *R*^2^ NS  substitutions | 0.62 | | | 0.51 | | | 0.56 | |
|  | SYN  substitutions | 0.66 | | | 0.49 | | | 0.65 | |
| Diversity binned by predicted effect of linked selection | Spearman's *ρ* | 0.913 | | | 0.890 | | |  | |
|  | Upper-to-lower   tails observed  diversity ratio | 5.3 | | | 5.0 | | |  | |
| Diversity reduction measures |  | 73% | | | 43% | | | 21% | |
|  | *k_B_* | 67% | | | . | | | . | |
|  | *k_S_* | 41% | | | 43% | | | 21% | |
|  | *k_B_*/(*k_B_*+*k_S_*) | 62% | | | . | | | . | |
|  | *k_S_*/(*k_B_*+*k_S_*) | 38% | | | 100% | | | 100% | |
| Coalescent rate measures | *r_B_*+*r_S_* | 3.18 | | | 0.87 | | | 0.29 | |
|  | *r_B_* | 2.26 | | | . | | | . | |
|  | *r_S_* | 0.92 | | | 0.87 | | | 0.29 | |
|  | *r_B_*/(*r_B_*+*r_S_*) | 71% | | | . | | | . | |
|  | *r_S_*/(*r_B_*+*r_S_*) | 29% | | | 100% | | | 100% | |

**Table S6. Comparison with the Sattath et al. (2011) inference method.**

An error in Sattath et al. (2011)**.** In the course of this analysis, we found an error in Sattath et al. (2011) that caused a three-fold increase in estimates of the fraction of substitutions associated with non-zero selection coefficients. While this does not change the qualitative conclusions in Sattath et al. (2011), it does imply that all estimated fractions in their Table S5 should be divided by 3. For example, for a distribution of fitness effects consisting of two point masses, the correct estimates are that 4.8% of the amino acid substitutions were driven by classic selective sweeps, 1.1% of them with a selection coefficient of *s*=5.1×10^-3^ and the remaining 3.7% with *s*=1.1×10^-4^.

Inferring selection parameters using the Wiehe, Kim and Stephan method. Wiehe and Stephan (1993) [49] introduced a method to infer compound classic sweep parameters from the observed relationship between levels of heterozygosity and recombination rates. Kim and Stephan (2000) [50] extended the underlying model to include both classic sweeps and background selection. Macpherson et al. [51] and Andolfatto [52] also extended the method, this time to infer compound classic sweep parameters from the relationship between levels of heterozygosity and rates of non-synonymous substitutions.

The model for expected heterozygosity underlying these studies can be summarized by

$\pi(c,d_{n})=\pi_{0}\frac{c\cdot Exp(-u_{del}/c)}{c+I\cdot d_{n}\cdot(\sum_{s} \alpha(s)\cdot s)\cdot Exp(-u_{del}/c)}$,

where *c* and *d_n_* are the rates of recombination and non-synonymous substitutions per bp per generation measured in a specified window, *π*_0_ is the expected heterozygosity in the absence of linked selection, *I*~0.075 is a constant, and the compound selection parameters are: *u_del_*, the total rate of deleterious mutations per bp per generation and $\sum_{s} \alpha(s)\cdot s$, where *α*(*s*) is the fraction of beneficial non-synonymous substitutions with selection coefficient *s*. To infer the compound parameter for background selection alone, the second term in the denominator is set to 0 (corresponding to no sweeps) and *u_del_* is inferred using least squares to fit the remaining functional form with the observed relationship between *π* and *c*. To infer the compound parameter for classic sweeps alone, the exponential is set to 1 (corresponding to *u_del_*=0); for the inference based on the relationship between *π* and *c*, *d_n_* is set to its genomic average, and for the inference based on the relationship between *π* and *d_n_*, *c* is set to its genomic average. In applying these methods to our data, we partition codons into 100 bins with equal amounts of data based on *d_n_*, measured in a window of 0.03 cM around each codon, or based on the local estimate of the recombination rate (corresponding to a spatial resolution of ~100 kb [13]).

Estimates of the compound selection parameters based on these inferences are shown in Table S7, alongside our own. As we note in the Results, the Wiehe, Kim and Stephan methods underestimate the compound parameters and thus the effects of linked selection because, by ignoring some genomic features affecting the strength of linked selection, they suffer from the equivalent of regression toward the mean (also see Fig 6 in the main text). This effect is best seen by comparing estimates for a single mode of linked selection, where all methods face the problem of absorbing other modes of linked selection. This underestimation effect is also apparent in the comparison of the Sattath et al. estimates and ours under a model of classic sweeps alone, as well as in the comparison of our inferences based on the joint model for background selection and classic sweeps with models of one or the other (Fig 6A in the main text).

| Model | $\sum_{s} \alpha(s)\cdot s$ | *U_del_* per diploid |
| --- | --- | --- |
| Background selection and classic sweeps | 3.5⨯10^-5^ | 1.60 |
| Background selection alone | . | 1.46 |
| Classic sweeps alone | 1.5⨯10^-4^ | . |
| Classic sweeps  based on Sattath et al. | 6.1⨯10^-5^ | . |
| Background selection  based on Charlesworth | . | 0.56 |
| Background selection  based on Kim & Stephan by recombination rate | . | 0.74 |
| Classic sweeps  based on Wiehe & Stephan by recombination rate | 4.2⨯10^-5^ | . |
| Classic sweeps  based on Macpherson et al. by NS divergence | 3.8⨯10^-5^ | . |

**Table S7. Estimates of compound selection parameters based on different inference methods.** The compound selection parameters estimated are: $\sum_{s} \alpha(s)\cdot s$ for non-synonymous substitutions and *U_del_*, the total number of deleterious mutations on autosomes per genome per generation.

# H. Sensitivity to the recombination rate threshold and to codon usage bias

The recombination rate threshold*.* To examine the sensitivity of our estimates to the choice of recombination rate threshold, we inferred the parameters of the combined model excluding sites with sex-averaged recombination rates <0.375 cM/Mb and <1.125 cM/Mb (instead of <0.75 cM/Mb), again excluding the distal 5% of chromosome arms (Table S8). We find that the goodness-of-fit statistics (evaluated across data from all sites with recombination rate >0.75 cM/Mb) and most parameter estimates are robust to the change of threshold. The one notable exception is that with the lower threshold, we no longer infer ~40% of substitutions at UTRs to have been driven by sweeps associated with very weak selection (with *s*=10^-5.5^). As a result, our total estimate of the fraction of beneficial substitutions at UTRs drops dramatically from ~47% to ~4%.

Further analysis suggests that our inference about a large proportion of weak sweeps at UTRs is reliable. Direct evidence comes from collated plots of the average diversity levels around substitutions in UTRs (Fig S5A). Our predictions based on a threshold of 0.375 cM/Mb or on a threshold of 0.75 but excluding the weakly selected sweeps overestimate the observed diversity levels near substitutions, as expected if weaker sweeps are indeed present. In contrast, when we use the predictions based on the higher thresholds of 0.75 cM/Mb or 1.25 cM/Mb, both of which include a large proportion of weakly selected sweeps, we explain the trough close to substitutions much better.

|  | Model | Background selection and classic sweeps | | | Background selection and classic sweeps | | | Background selection and classic sweeps | | |
| --- | --- | --- | --- | --- | --- | --- | --- | --- | --- | --- |
|  | Recombination   threshold | 1.125 cM/Mb | | | 0.75 cM/Mb | | | 0.375 cM/Mb | | |
| Parameters | Annotation | Exons | UTRs | Introns | Exons | UTRs | Introns | Exons | UTRs | Introns |
|  | BS parameters |  |  |  |  |  |  |  |  |  |
|  | *u*(*t*=10^-1.5^) / *μ* | 321% | 710% | 24% | 377% | 577% | 19% | 403% | 671% | 3% |
|  | *u*(*t*=10^-2.5^) / *μ* | . | . | . | 2% | 2% | . | 3% | 18% | . |
|  | *u*(*t*=10^-3.5^) / *μ* | 63% | . | . | 56% | . | . | 56% | . | . |
|  | *u*(*t*=10^-4.5^) / *μ* | . | 12% | . | 2% | 23% | . | . | 11% | . |
|  | *u*(*t*=10^-5.5^) / *μ* | . | . | . | . | 2% | . | . | . | . |
|  | CS parameters |  |  |  |  |  |  |  |  |  |
|  | *α*(*s*=10^-1.5^) | . | . | . | . | . | . | . | . | . |
|  | *α*(*s*=10^-2.5^) | 0.7% | . | . | 0.6% | . | . | 0.4% | . | . |
|  | *α*(*s*=10^-3.5^) | 4.0% | . | . | 3.5% | . | . | 4.1% | . | . |
|  | *α*(*s*=10^-4.5^) | . | 6.4% | . | . | 5.1% | . | . | 3.7% | . |
|  | *α*(*s*=10^-5.5^) | 44.2% | 34.2% | 0.2% | 36.3% | 42.1% | . | 31.9% | 0.1% | . |
|  | *ΔCL* | 4.08⨯10^-4^ | | | 4.11⨯10^-4^ | | | 4.06⨯10^-4^ | | |
| Diversity binned in local windows | *R*^2^ 1 Mb | 0.63 | | | 0.62 | | | 0.63 | | |
|  | 100 kb | 0.30 | | | 0.30 | | | 0.30 | | |
|  | 10 kb | 0.18 | | | 0.18 | | | 0.18 | | |
|  | 1 kb | 0.14 | | | 0.14 | | | 0.14 | | |
| Diversity binned by distance from substitution | *R*^2^ NS  substitutions | 0.61 | | | 0.58 | | | 0.56 | | |
|  | SYN  substitutions | 0.65 | | | 0.57 | | | 0.56 | | |
| Diversity binned by predicted effect of linked selection | Spearman's *ρ* | 0.796 | | | 0.795 | | | 0.794 | | |
|  | Upper-to-lower   tails observed  diversity ratio | 3.9 | | | 4.0 | | | 4.3 | | |
| Diversity reduction measures |  | 73% | | | 73% | | | 72% | | |
|  | *k_B_* | 66% | | | 67% | | | 68% | | |
|  | *k_S_* | 43% | | | 41% | | | 34% | | |
|  | *k_B_*/(*k_B_*+*k_S_*) | 61% | | | 62% | | | 67% | | |
|  | *k_S_*/(*k_B_*+*k_S_*) | 39% | | | 38% | | | 33% | | |
| Coalescent rate measures | *r_B_*+*r_S_* | 3.2 | | | 3.2 | | | 3.0 | | |
|  | *r_B_* | 2.2 | | | 2.3 | | | 2.3 | | |
|  | *r_S_* | 1.0 | | | 0.9 | | | 0.6 | | |
|  | *r_B_*/(*r_B_*+*r_S_*) | 69% | | | 71% | | | 78% | | |
|  | *r_S_*/(*r_B_*+*r_S_*) | 31% | | | 29% | | | 22% | | |

**Table S8. Sensitivity of our inference to the choice of recombination rate threshold.** Goodness-of-fit measures are evaluated for data from regions with recombination rate > 0.75 cM/Mb.


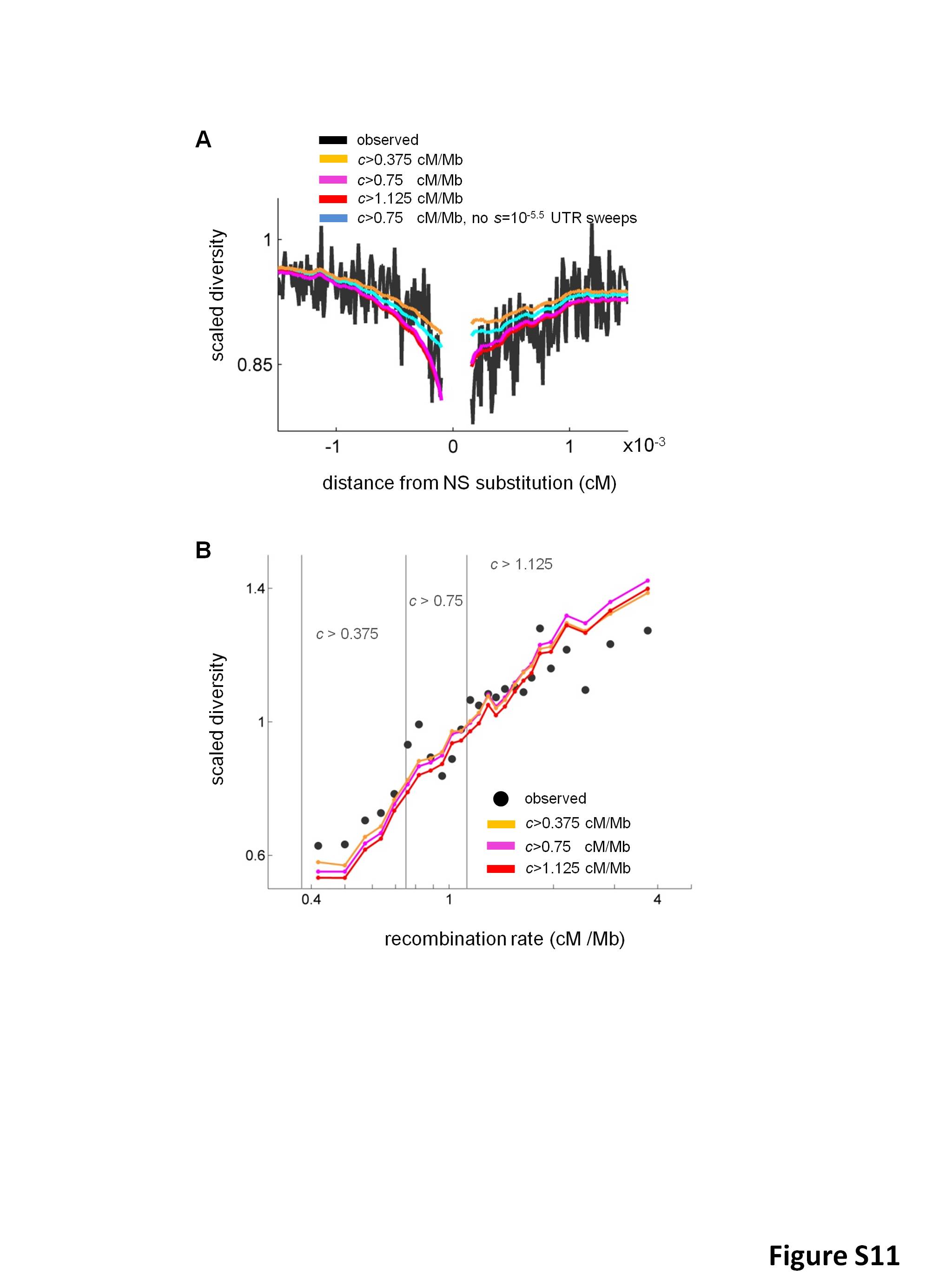


**Fig S5. Estimates associated with weak sweeps are more reliable with the higher recombination threshold.**

Moreover, we expect that including regions with lower recombination rates will lead to underestimates of the fraction of substitutions caused by sweeps, particularly for weak sweeps at UTRs, for the following reasons:

(1) Interference among beneficial mutations, which we do not model, is greater in regions with lower recombination [53-63], and will likely have a greater effect on the number of fixations caused by weakly beneficial mutations (because when strongly and weakly beneficial alleles interfere, the stronger one is more likely to prevail; also see [64]). Also, selection is less effective in regions with lower recombination, so both weakly selected beneficial and deleterious mutations will be less effectively selected and may even be rendered effectively neutral. If these speculations are true, then we would expect there to be fewer weakly selected sweeps in regions with lower recombination. Indirect evidence for fewer selected mutations causing the reduction in diversity in regions with lower recombination rates can be seen by comparing observed and predicted levels as a function of recombination rate (Fig S5B). Notably, we see that our predictions tend to overestimate the reduction in diversity levels in regions with lower recombination rates and to overestimate them in regions with higher rates.

(2) Rather than reflecting an average over the range of recombination rates used, our estimates are disproportionally affected by data from regions with lower recombination rates—simply because each sweep in these regions affects many more codons than in higher recombination regions. Thus, by including lower recombination regions, we downwardly bias our estimates of the fraction of beneficial substitutions, and most strongly those under weak selection.

(3) This bias should be the stronger for weakly selected substitutions in UTRs than in exons, given that we measure diversity levels in exons, and therefore have proportionately fewer synonymous sites at short genetic distances from substitutions in UTRs in regions of high recombination than we do for exons. Consistent with this interpretation, when we decrease the recombination rate threshold below 0.1 cM/Mb, thus increasing the influence of low recombination regions on our estimates even further, estimates of the fraction of weakly beneficial amino acid substitutions begin to decrease.

Given these considerations, we conclude that our inference about a substantial number of weakly selected substitutions at UTRs to be correct. We choose to work with a threshold of 0.75 cM/Mb, because our results appear to be robust to increasing it to 1.25cM/Mb. Thus, compromising between minimizing the biases associated with regions with low recombination and maximizing our data lead us to this threshold. We note finally that even if weakly selected sweep are less common in low recombination regions, these regions still comprise a fairly small portion of the genome, such that our estimates based on our higher threshold are more reflective of the genomic rates.

Codon usage bias*.* While we use all synonymous changes as our proxy for neutral diversity, weak selection for preferred codon usage is known to exist in *D. melanogaster* ([5,65,66] (but see [67,68]). To examine whether such selection affects our results, we apply our inference to subsets of synonymous changes that are thought to be subject to considerably less selection. Specifically, we restrict the analysis to (i) preferred codons and preferred to preferred changes (P2P|P), as defined by Vicario et al. (method A) [69]; or to (ii) unpreferred codons and unpreferred to unpreferred changes (U2U|U). This restriction comes at the cost of a considerable reduction in the amount of data: for (i), this restricts us to 61% of codons and 6% of synonymous changes, and for (ii), to 39% of codons and 10% of synonymous changes. To control for variation in mutation rates, we use estimates of synonymous divergence between *D. simulans* and *D. yakuba* in windows of 1780 kb (not correcting for multiple hits in this case) using the same class of codons and changes as we do for polymorphism.

Table S9 shows the parameter estimates and summaries for the two subsets of codons alongside those for the entire dataset. To gauge the uncertainty in our estimates due to the dramatic reduction in sample size (~10-20-fold), we divide each dataset into two subsets with half the data (set A and set B in Table S9, see Section C for details, but without controlling for the correlation between diversity and recombination) and infer the parameters for each half separately. This analysis suggests that our estimates of individual selection parameters based on restricted (but not the full) datasets are associated with considerable uncertainty, so that drawing conclusions about the effects of weak selection based on these estimates would be misleading. Instead, we limit ourselves to qualitative patterns and summaries that appear to be fairly robust despite the smaller amount of data.

These patterns and summaries suggest that our inference is fairly insensitive to weak selection on synonymous codon usage. Notably, both background selection and classic sweeps substantially reduce average diversity levels (together between 49-76%), with a larger (2~3-fold) reduction due to background selection compared to that of classic sweeps. The effects of classic sweeps are mainly due to three classes of substitutions and selection effects, whose presence appear to be robust but whose exact size varies: a minority of non-synonymous substitutions (0.6-3.7%) with large effects (*s*=10^-2.5^-10^-3.5^) and a majority (~30-50%) with small effect sizes (*s*=10^-5.5^), as well as many substitutions at UTRs (~17-90%) with small effects (*s*=10^-4.5^-10^-5.5^). In turn, the effects of background selection are mainly due to strongly selected mutations (*t*=10^-1.5^), with a smaller contribution from intermediate-effect mutations (*t*=10^-2.5^-10^-4.5^) in exons. In conclusion, we see no clear indication that weak selection biases our inferences, though we lack power to detect a small effect.

|  |  | **All** | | | **P2P|P** | | | **U2U|U** | | |
| --- | --- | --- | --- | --- | --- | --- | --- | --- | --- | --- |
| **Full** |  |  | 76% |  |  | 49% |  |  | 72% |  |
|  | *k_B_*/(*k_B_+k_S_*) |  | 63% |  |  | 66% |  |  | 53% |  |
|  | *k_S_*/(*k_B_+k_S_*) |  | 37% |  |  | 34% |  |  | 47% |  |
|  | Annotation | Exons | UTRs | Introns | Exons | UTRs | Introns | Exons | UTRs | Introns |
|  | *u*(*t*=10^-1.5^) / *μ* | 451.0% | 612.0% | 11.0% | 2.0% | 152.0% | 50.0% | 390.0% | 540.0% | . |
|  | *u*(*t*=10^-2.5^) / *μ* | . | . | . | 6.0% | 81.0% | . | . | 2.0% | . |
|  | *u*(*t*=10^-3.5^) / *μ* | 61.0% | . | . | 14.0% | . | . | 65.0% | . | . |
|  | *u*(*t*=10^-4.5^) / *μ* | 8.0% | 10.0% | . | 9.0% | 1.0% | . | . | 36.0% | . |
|  | *u*(*t*=10^-5.5^) / *μ* | . | . | . | . | 17.0% | 1.0% | . | . | . |
|  | *α*(*s*=10^-1.5^) | . | . | . | . | . | . | . | . | . |
|  | *α*(*s*=10^-2.5^) | 0.8% | . | . | 0.5% | . | . | 0.6% | 0.3% | . |
|  | *α*(*s*=10^-3.5^) | 2.9% | . | . | 1.4% | . | . | . | . | . |
|  | *α*(*s*=10^-4.5^) | . | 0.9% | . | . | 16.6% | . | . | 5.1% | 2.6% |
|  | *α*(*s*=10^-5.5^) | 32.9% | 46.4% | . | 31.8% | . | 58.1% | 50.7% | 87.5% | . |
| **Set A** |  |  | 77% |  |  | 54% |  |  | 75% |  |
|  | *k_B_*/(*k_B_+k_S_*) |  | 66% |  |  | 75% |  |  | 70% |  |
|  | *k_S_*/(*k_B_+k_S_*) |  | 34% |  |  | 25% |  |  | 30% |  |
|  | Annotation | Exons | UTRs | Introns | Exons | UTRs | Introns | Exons | UTRs | Introns |
|  | *u*(*t*=10^-1.5^) / *μ* | 482.0% | 583.0% | . | . | 3.0% | 4.0% | 320.0% | 795.0% | 8.0% |
|  | *u*(*t*=10^-2.5^) / *μ* | . | . | . | 144.0% | 2.0% | . | 4.0% | 3.0% | . |
|  | *u*(*t*=10^-3.5^) / *μ* | 86.0% | . | . | 8.0% | . | . | 120.0% | 3.0% | . |
|  | *u*(*t*=10^-4.5^) / *μ* | 3.0% | 15.0% | . | . | 58.0% | . | 2.0% | 2.0% | . |
|  | *u*(*t*=10^-5.5^) / *μ* | . | . | . | . | 2.0% | . | . | 2.0% | . |
|  | *α*(*s*=10^-1.5^) | . | . | . | . | . | . | . | . | . |
|  | *α*(*s*=10^-2.5^) | 0.5% | . | . | 0.2% | . | . | . | 0.4% | . |
|  | *α*(*s*=10^-3.5^) | 2.9% | . | . | 3.0% | . | . | . | . | . |
|  | *α*(*s*=10^-4.5^) | . | . | . | . | 3.1% | . | . | . | 0.9% |
|  | *α*(*s*=10^-5.5^) | 27.6% | 30.4% | . | 26.6% | . | 33.8% | 47.6% | 62.4% | . |
| **Set B** |  |  | 74% |  |  | 46% |  |  | 71% |  |
|  | *k_B_*/(*k_B_+k_S_*) |  | 60% |  |  | 59% |  |  | 58% |  |
|  | *k_S_*/(*k_B_+k_S_*) |  | 40% |  |  | 41% |  |  | 42% |  |
|  | Annotation | Exons | UTRs | Introns | Exons | UTRs | Introns | Exons | UTRs | Introns |
|  | *u*(*t*=10^-1.5^) / *μ* | 253.0% | 540.0% | 101.0% | 2.0% | 299.0% | 56.0% | 351.0% | 655.0% | . |
|  | *u*(*t*=10^-2.5^) / *μ* | . | . | . | 1.0% | 3.0% | . | . | 2.0% | . |
|  | *u*(*t*=10^-3.5^) / *μ* | 43.0% | . | . | . | . | . | 17.0% | 18.0% | . |
|  | *u*(*t*=10^-4.5^) / *μ* | 13.0% | . | . | 14.0% | . | . | 1.0% | 45.0% | . |
|  | *u*(*t*=10^-5.5^) / *μ* | . | . | . | . | 17.0% | 3.0% | . | 6.0% | . |
|  | *α*(*s*=10^-1.5^) | . | . | . | . | . | . | . | . | . |
|  | *α*(*s*=10^-2.5^) | 1.0% | . | . | 0.5% | . | . | 1.2% | 0.1% | . |
|  | *α*(*s*=10^-3.5^) | 3.0% | . | . | 0.2% | . | 0.1% | . | . | . |
|  | *α*(*s*=10^-4.5^) | . | 6.5% | . | 5.2% | 30.9% | . | . | 13.2% | 1.6% |
|  | *α*(*s*=10^-5.5^) | 39.5% | 37.5% | . | 27.6% | 15.7% | 99.5% | 61.7% | 113.6% | 17.8% |

**Table S9. Sensitivity of our inference to selection for synonymous codon usage.** All summaries and estimates correspond to inferences based on the combined model with background selection and classic sweeps. Estimates associated with intergenic regions are negligible and are therefore omitted from the table.

# I. Inference based on additional models

In the Results, we focus on three models. The main one considers the combined effects of background selection and classic selective sweeps at each of four functional annotations, with a grid of selection coefficients for each annotation and mode of selection consisting of 5 point masses, i.e., *t* and *s* = 10^-5.5^, 10^-4.5^, 10^-3.5^, 10^-2.5^ and 10^-1.5^. To investigate the support for including both modes of selection in our inferences, we also report the results for models with background selection alone and classic sweeps alone. To complete our analysis of the main model, here, we consider two additional variants of it. First, we consider how our inference is affected by using a finer grid of selection coefficients, where instead of 5 point masses, we use 11 with *t* and *s* = 10^-6^, 10^-5.5^, 10^-5^, 10^‑4.5^, 10^-4^, 10^-3.5^, 10^-3^, 10^-2.5^, 10^-2^, 10^-1.5^ and 10^-1^. Second, we consider how our inference is affected by focusing only on the annotations whose effects appear to dominate, namely on exons and UTRs alone. The parameter estimates and goodness-of-fit statistics for these models are shown in Table S10.

We find that both variants of the model have little effect on our inferences. Using a finer grid of selection coefficients offers little improvement to the quality of our predictions and has only minor effects on our parameter estimates, leading to our choice to work primarily with the simpler model with only 5 grid points. We also find that including long introns and intergenic regions has little effect. This finding does not imply little selection in these annotations (see [9,11,70]) as we use synonymous changes to measure diversity levels and thus have less power to make reliable inferences about selection in more distant annotations.

|  | Model | Background selection and classic sweeps | | | Background selection and classic sweeps  11 point masses grids | | | BS and CS  Selection at exons and UTRs only | |
| --- | --- | --- | --- | --- | --- | --- | --- | --- | --- |
| Parameters |  | 73% | | | 86% | | | 75% | |
|  | Annotation | Exons | UTRs | Introns | Exons | UTRs | Introns | Exons | UTRs |
|  | BS parameters |  |  |  |  |  |  |  |  |
|  | *u*(*t*=10^-1^) / *μ* |  |  |  | 679% | 298% | 10% |  |  |
|  | *u*(*t*=10^-1.5^) / *μ* | 377% | 577% | 19% | 193% | 536% | . | 441% | 665% |
|  | *u*(*t*=10^-2^) / *μ* |  |  |  | 1% | 3% | . |  |  |
|  | *u*(*t*=10^-2.5^) / *μ* | 2% | 2% | . | . | 2% | . | . | . |
|  | *u*(*t*=10^-3^) / *μ* |  |  |  | . | 1% | . |  |  |
|  | *u*(*t*=10^-3.5^) / *μ* | 56% | . | . | 11% | . | . | 58% | . |
|  | *u*(*t*=10^-4^) / *μ* |  |  |  | 41% | 1% | . |  |  |
|  | *u*(*t*=10^-4.5^) / *μ* | 2% | 23% | . | 1% | . | . | . | 28% |
|  | *u*(*t*=10^-5^) / *μ* |  |  |  | . | . | . |  |  |
|  | *u*(*t*=10^-5.5^) / *μ* | . | 2% | . | . | . | . | . | . |
|  | *u*(*t*=10^-6^) / *μ* |  |  |  | . | . | . |  |  |
|  | CS parameters |  |  |  |  |  |  |  |  |
|  | *α*(*s*=10^-1^) |  |  |  | 0.1% | . | . |  |  |
|  | *α*(*s*=10^-1.5^) | . | . | . | . | . | . | . | . |
|  | *α*(*s*=10^-2^) |  |  |  | . | . | . |  |  |
|  | *α*(*s*=10^-2.5^) | 0.6% | . | . | 0.6% | . | . | 0.6% | . |
|  | *α*(*s*=10^-3^) |  |  |  | . | 0.4% | . |  |  |
|  | *α*(*s*=10^-3.5^) | 3.5% | 0.0% | . | 1.5% | . | . | 3.6% | . |
|  | *α*(*s*=10^-4^) |  |  |  | 3.9% | . | . |  |  |
|  | *α*(*s*=10^-4.5^) | 0.0% | 5.1% | . | . | 0.2% | . | . | 4.6% |
|  | *α*(*s*=10^-5^) |  |  |  | 0.4% | 24.0% | . |  |  |
|  | *α*(*s*=10^-5.5^) | 36.3% | 42.1% | . | 4.8% | 1.3% | . | 37.1% | 44.0% |
|  | *α*(*s*=10^-6^) |  |  |  | 40.4% | . | . |  |  |
|  | *ΔCL* | 3.9⨯10^-4^ | | | 4.0⨯10^-4^ | | | 3.9⨯10^-4^ | |
| Diversity binned in local windows | *R*^2^ 1 Mb | 0.71 | | | 0.69 | | | 0.71 | |
|  | 100 kb | 0.44 | | | 0.43 | | | 0.44 | |
|  | 10 kb | 0.26 | | | 0.26 | | | 0.26 | |
|  | 1 kb | 0.20 | | | 0.20 | | | 0.20 | |
| Diversity binned by distance from substitution | *R*^2^ NS substitutions | 0.62 | | | 0.64 | | | 0. 63 | |
|  | SYN substitutions | 0.66 | | | 0.65 | | | 0.68 | |
| Diversity binned by predicted effect of linked selection | Spearman's *ρ* | 0.913 | | | 0.913 | | | 0.910 | |
|  | Upper-to-lower tails observed diversity ratio | 5.3 | | | 5.1 | | | 5.3 | |
| Diversity reduction measures |  | 73% | | | 86% | | | 75% | |
|  | *k_B_* | 67% | | | 81% | | | 69% | |
|  | *k_S_* | 41% | | | 69% | | | 43% | |
|  | *k_B_*/(*k_B_*+*k_S_*) | 62% | | | 54% | | | 62% | |
|  | *k_S_*/(*k_B_*+*k_S_*) | 38% | | | 46% | | | 38% | |
| Coalescent rate measures | *r_B_*+*r_S_* | 3.18 | | | 7.59 | | | 3.51 | |
|  | *r_B_* | 2.26 | | | 4.72 | | | 2.52 | |
|  | *r_S_* | 0.92 | | | 2.87 | | | 0.98 | |
|  | *r_B_*/(*r_B_*+*r_S_*) | 71% | | | 62% | | | 72% | |
|  | *r_S_*/(*r_B_*+*r_S_*) | 29% | | | 38% | | | 28% | |

**Table S10. Comparison to models with a finer grid of selection coefficients and fewer functional annotations.**

# J. Additional figures and tables

**
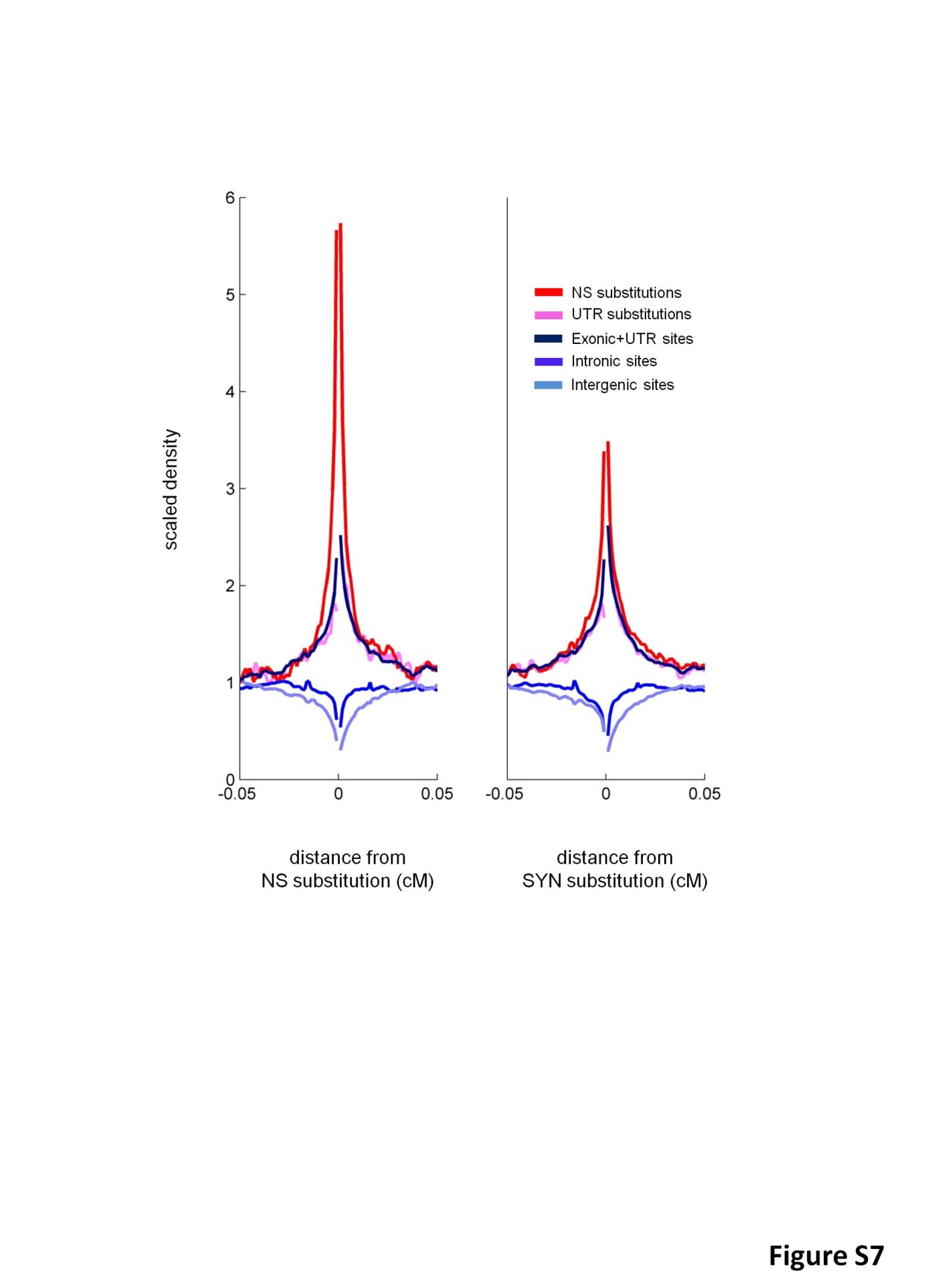
**

**Fig S6**. **Densities of functional annotations around synonymous and non-synonymous substitutions (complementary to Fig 4 in the main text).** Shown are the densities of all annotations for which we inferred non-negligible selection parameters: non-synonymous (NS) substitutions (red), UTR substitutions (pink), exonic and UTR sites (black), intronic sites (dark blue), and intergenic sites (light blue). Densities are plotted relative to their level at distance 0.1 cM away from the focal substitutions**.**


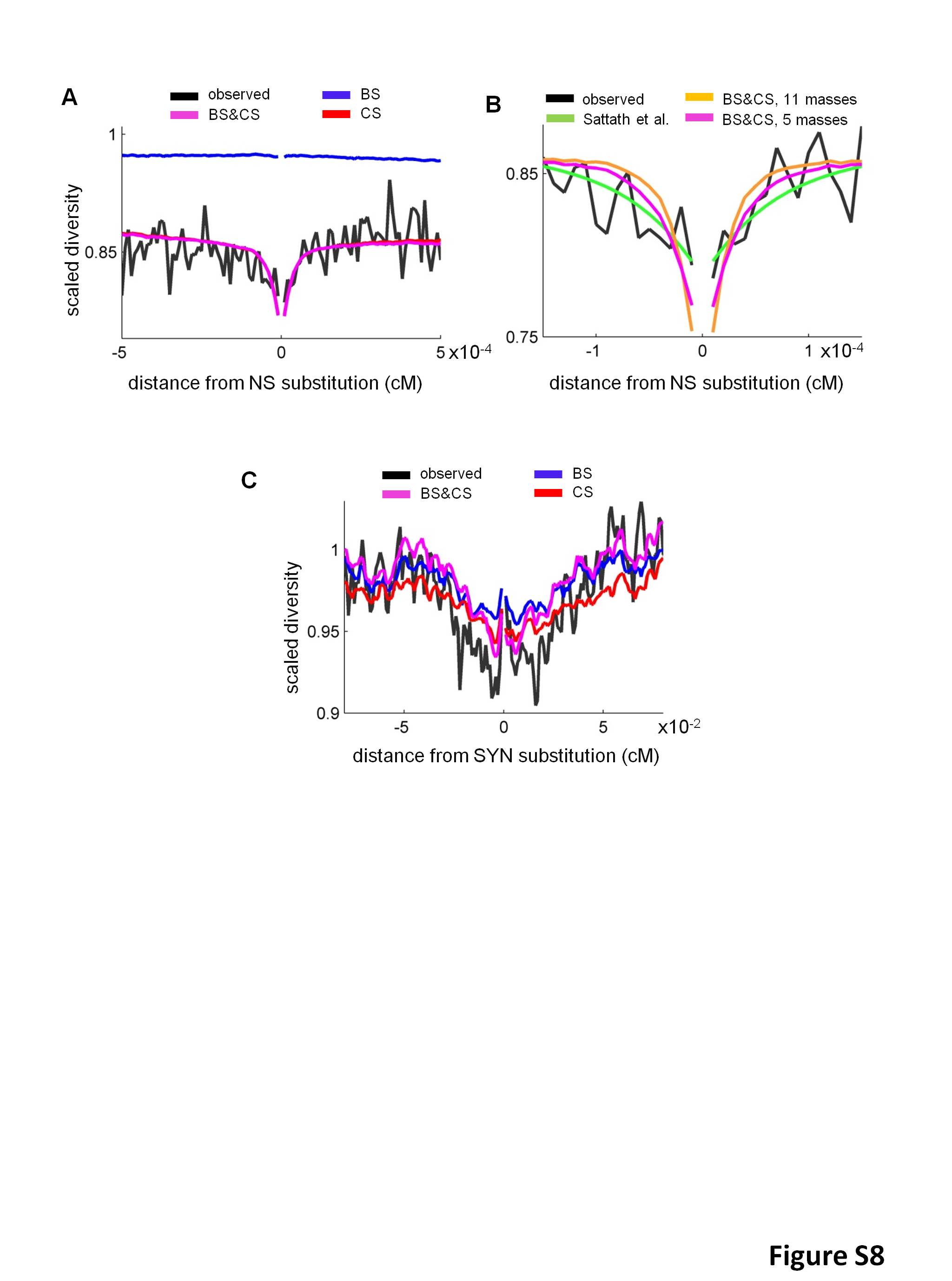


**Fig S7**. **Observed and predicted scaled diversity levels around exonic substitutions (complementary to Fig 3B and 5A in the main text).** (**A**) A close up on Fig 5A in the main text near non-synonymous substitutions (<5×10^-4^ cM). (**B**) A close up on Fig 3B in the main text near non-synonymous substitutions (<1.5×10^-4^ cM). Also shown are the predictions based on the model with a finer grid of selection coefficients (11 points). (**C**) The equivalent of Fig 5A in the main text around synonymous (rather than non-synonymous) substitutions.

**
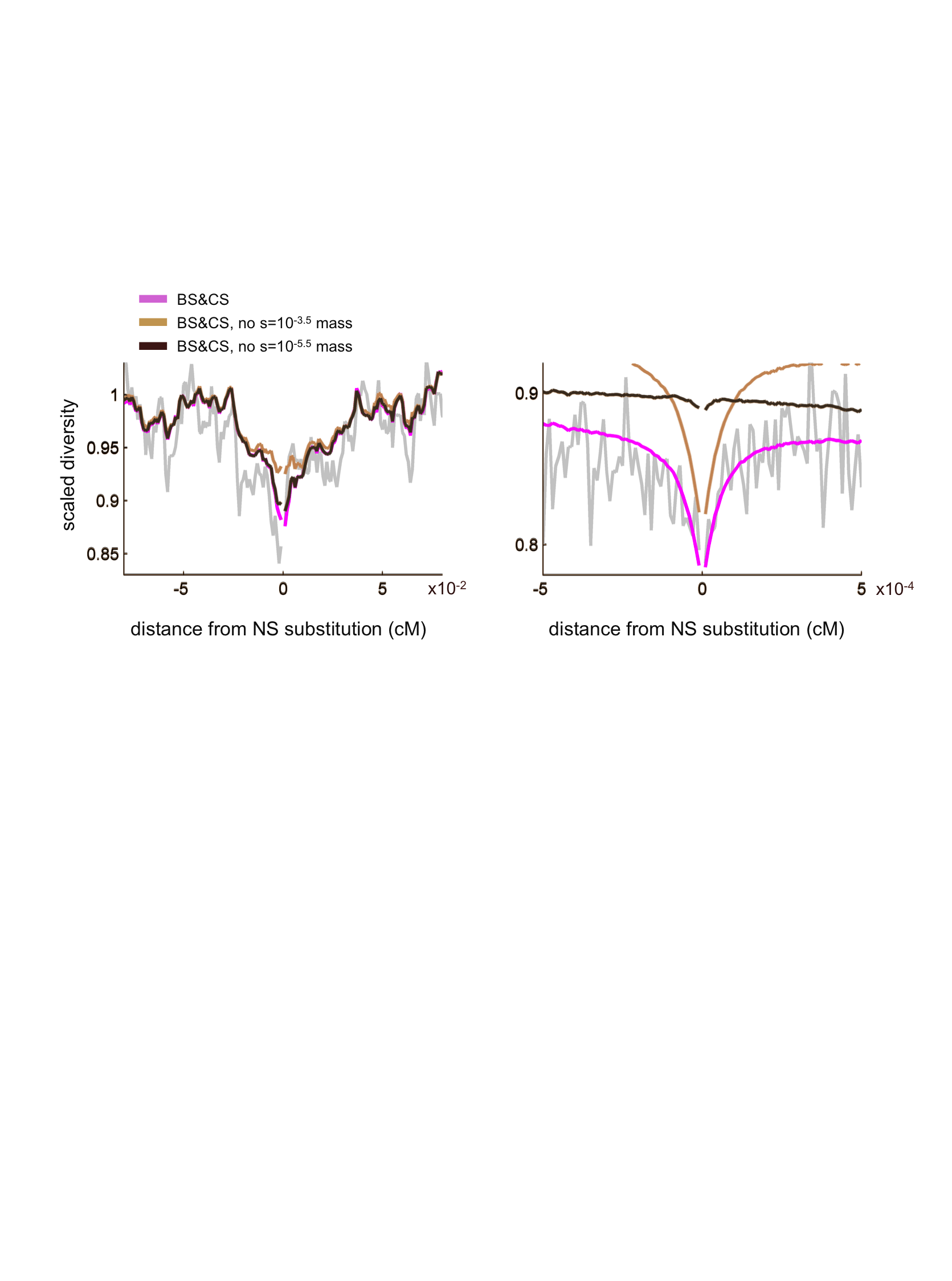
**

**Fig S8**. **The contribution of the two inferred modes of sweeps to diversity levels around non-synonymous substitutions.** To isolate the contribution of each mode, we remove it from our predictions. Removing sweeps with *s*=10^-3.5^ (brown, left) removes a trough in diversity levels on the scale of 10^-2^ cM that is apparent in the full model (pink, left) and in the data (gray, left). In turn, removing sweeps with *s*=10^-5.5^ (black, right) removes a trough on the scale of 10^-4^ cM apparent in both the full model (pink, right) and data (gray, right). In the models where a mode of sweep was excluded, we scaled the predicted diversity levels in the absence of linked selection such that the predicted genome-wide average will coincide with the full model and data.

**Table S11. Detailed parameter estimates for all models.**

**A. Classic sweeps parameters**. For introns we always find *α*=0 and have therefore omitted these estimates from the table.

| Model | Background selection and classic sweeps | | Background selection and classic sweeps | | Background selection and classic sweeps | | Classic sweeps | | Classic sweeps |
| --- | --- | --- | --- | --- | --- | --- | --- | --- | --- |
|  |  | | 11 masses | | constrained | |  | | Sattath et al. |
|  | 73% | | 86% | | 59% | | 43% | | 35% |
| $\sum_{s} \alpha(s)\cdot s$ | 3.5⨯10^-5^ | | 1.2⨯10^-4^ | | 3.3⨯10^-5^ | | 1.5⨯10^-4^ | | 6.1⨯10^-5^ |
| Annotation | Exons | UTRs | Exons | UTRs | Exons | UTRs | Exons | UTRs | Exons |
| $\sum_{s} \alpha(s)\cdot s$ | 3.2⨯10^-5^ | 3.0⨯10^-6^ | 1.1⨯10^-4^ | 6.3⨯10^-6^ | 3.0⨯10^-5^ | 3.0⨯10^-6^ | 1.1⨯10^-4^ | 3.6⨯10^-5^ | 6.1⨯10^-5^ |
| *α* | 40% | 47% | 52% | 26% | 42% | 51% | 40% | 51% | 20% |
| *α*(*s*=10^-1.0^) |  |  | 0.1% | . |  |  |  |  |  |
| *α*(*s*=10^-1.5^) | . | . | . | . | . | . | 0.3% | 0.1% |  |
| *α*(*s*=10^-2.0^) |  |  | . | . |  |  |  |  | 1.5% (s=4.1E-03) |
| *α*(*s*=10^-2.5^) | 0.6% | . | 0.6% | . | 0.6% | . | 0.4% | . |  |
| *α*(*s*=10^-3.0^) |  |  | . | 0.4% |  |  |  |  |  |
| *α*(*s*=10^-3.5^) | 3.5% | . | 1.5% | . | 3.5% | . | 3.7% | . |  |
| *α*(*s*=10^-4.0^) |  |  | 3.9% | . |  |  |  |  |  |
| *α*(*s*=10^-4.5^) | . | 5.1% | . | 0.2% | . | 4.7% | . | 4.4% |  |
| *α*(*s*=10^-5.0^) |  |  | 0.4% | 24.0% |  |  |  |  | 18.8% (s=5.8E-06) |
| *α*(*s*=10^-5.5^) | 36.3% | 42.1% | 4.8% | 1.3% | 38.1% | 45.9% | 35.7% | 46.1% |  |
| *α*(*s*=10^-6.0^) |  |  | 40.4% | . |  |  |  |  |  |

**B. Background selection parameters.**

| Model | Background selection  and classic sweeps | | | | Background selection | | | | | | | Background selection  and classic sweeps | | | |
| --- | --- | --- | --- | --- | --- | --- | --- | --- | --- | --- | --- | --- | --- | --- | --- |
|  |  | | | |  | | | | | | | 11 masses | | | |
|  | 73% | | | | 65% | | | | | | | 86% | | | |
| *U*_del_ (per diploid) | 1.60 | | | | 1.46 | | | | | | | 2.90 | | | |
| Annotation | Exons (20%) | UTRs (5%) | Introns (40%) | Intergenic (35%) | Exons | | UTRs | | Introns | | Intergenic | Exons | UTRs | Introns | Intergenic |
| *u*_del_ / *μ* | 437% | 603% | 19% | . | 448% | | 456% | | 17% | | . | 926% | 842% | 10% | 1% |
| *u*(*t*=10^-1.0^) / *μ* |  |  |  |  |  | |  | |  | |  | 679% | 298% | 10% | 1% |
| *u*(*t*=10^-1.5^) / *μ* | 377% | 577% | 19% | . | 369% | | 453% | | 17% | | . | 193% | 536% | . | . |
| *u*(*t*=10^-2.0^) / *μ* |  |  |  |  |  | |  | |  | |  | 1% | 3% | . | . |
| *u*(*t*=10^-2.5^) / *μ* | 2% | 2% | . | . | . | | . | | . | | . | . | 2% | . | . |
| *u*(*t*=10^-3.0^) / *μ* |  |  |  |  |  | |  | |  | |  | . | 1% | . | . |
| *u*(*t*=10^-3.5^) / *μ* | 56% | . | . | . | 77% | | . | | . | | . | 11% | . | . | . |
| *u*(*t*=10^-4.0^) / *μ* |  |  |  |  |  | |  | |  | |  | 41% | 1% | . | . |
| *u*(*t*=10^-4.5^) / *μ* | 2% | 23% | . | . | 2% | | 3% | | . | | . | 1% | . | . | . |
| *u*(*t*=10^-5.0^) / *μ* |  |  |  |  |  | |  | |  | |  | . | . | . | . |
| *u*(*t*=10^-5.5^) / *μ* | . | 2% | . | . | . | | . | | . | | . | . | . | . | . |
| *u*(*t*=10^-6.0^) / *μ* |  |  |  |  |  | |  | |  | |  | . | . | . | . |
| Model | Background selection  and classic sweeps | | | | Background selection | | | | | | | Background selection | | | |
|  | constrained | | | | constrained | | | | | | | Charlesworth | | | |
|  | 59% | | | | 49% | | | | | | | 37% | | | |
| *U*_del_ (per diploid) | 0.92 | | | | 0.90 | | | | | | | 0.56 | | | |
| Annotation | Exons | UTRs | Introns | Intergenic | Exons | UTRs | | Introns | | Intergenic | | Exons | UTRs | Introns | Intergenic |
| *u*_del_ / *μ* | 90% | 88% | 87% | 44% | 90% | 88% | | 85% | | 38% | | 72% | 38% | 38% | 38% |
| *u*(*t*=10^-1.0^) / *μ* |  |  |  |  |  |  | |  | |  | |  |  |  |  |
| *u*(*t*=10^-1.5^) / *μ* | 18% | 67% | 85% | 44% | 1% | 84% | | 85% | | 38% | | . | . | . | . |
| *u*(*t*=10^-2.0^) / *μ* |  |  |  |  |  |  | |  | |  | |  |  |  |  |
| *u*(*t*=10^-2.5^) / *μ* | 2% | 2% | 1% | . | 2% | 3% | | . | | . | | 25% | 7% | 7% | 7% |
| *u*(*t*=10^-3.0^) / *μ* |  |  |  |  |  |  | |  | |  | |  |  |  |  |
| *u*(*t*=10^-3.5^) / *μ* | 69% | . | . | . | 85% | . | | . | | . | | 27% | 8% | 8% | 8% |
| *u*(*t*=10^-4.0^) / *μ* |  |  |  |  |  |  | |  | |  | |  |  |  |  |
| *u*(*t*=10^-4.5^) / *μ* | 1% | 20% | . | . | 2% | 2% | | . | | . | | 15% | 10% | 10% | 10% |
| *u*(*t*=10^-5.0^) / *μ* |  |  |  |  |  |  | |  | |  | |  |  |  |  |
| *u*(*t*=10^-5.5^) / *μ* | . | . | . | . | . | . | | . | | . | | 5% | 12% | 12% | 12% |
| *u*(*t*=10^-6.0^) / *μ* |  |  |  |  |  |  | |  | |  | |  |  |  |  |

**Table S12. Goodness-of-fit and other summaries for all models.**

| Model | BS & CS | BS & CS | BS & CS | BS & CS | BS | BS | CS | BS | CS | BS | CS | CS |  |
| --- | --- | --- | --- | --- | --- | --- | --- | --- | --- | --- | --- | --- | --- |
|  |  | 11 masses | constrained | excluding UTR sweeps |  | constrained |  | Charlesworth | Sattath et al. | Kim & Stephan by *c* | Wiehe & Stephan by *c* | WIehe & Stephan by *D_n_* |  |
| *ΔCL* | 3.9⨯10^-4^ | 4.0⨯10^-4^ | 3.6⨯10^-4^ | 3.8⨯10^-4^ | 2.8⨯10^-4^ | 2.5⨯10^-4^ | 2.4⨯10^-4^ | -6.7 ⨯10^-5^ |  |  |  |  |  |
| Diversity binned in local windows | *R*^2^ 1 Mb | 0.71 | 0.69 | 0.69 | 0.72 | 0.76 | 0.72 | 0.67 | 0.58 |  |  |  |  |
|  | 100 kb | 0.44 | 0.43 | 0.43 | 0.45 | 0.42 | 0.41 | 0.39 | 0.19 |  |  |  |  |
|  | 10 kb | 0.26 | 0.26 | 0.24 | 0.27 | 0.23 | 0.22 | 0.21 | 0.09 |  |  |  |  |
|  | 1 kb | 0.20 | 0.20 | 0.19 | 0.20 | 0.18 | 0.16 | 0.16 | 0.08 |  |  |  |  |
| Diversity binned by distance from substitution | *R*^2^  NS substitutions | 0.62 | 0.64 | 0.61 | 0.64 | 0.27 | 0.24 | 0.51 | 0 | 0.56 |  |  |  |
|  | *R*^2^  SYN   substitutions | 0.66 | 0.65 | 0.69 | 0.68 | 0.53 | 0.53 | 0.49 | 0.05 | 0.65 |  |  |  |
| Diversity binned by predicted linked selection effect | Spearman's *ρ* | 0.913 | 0.913 | 0.905 | 0.906 | 0.745 | 0.737 | 0.89 | 0.773 | 0.869 | 0.732 | 0.732 | 0.807 |
|  | Upper tail diversity reduction | 60% | 81% | 36% | 62% | 57% | 32% | 18% | 28% | -13%^(*)^ | 9% | 11% | -5%^(*)^ |
|  | Upper-to-lower tails observed diversity ratio | 5.3 | 5.1 | 5.4 | 5.4 | 4.4 | 4.3 | 5.0 | 3.5 | 4.5 | 3.2 | 3.2 | 4.1 |
| Diversity reduction measures | $\pi_{0}/\bar{\pi}$ | 4.4 | 9.0 | 2.8 | 4.7 | 3.4 | 2.3 | 2.0 | 1.8 | 1.5 | 1.5 | 1.5 | 1.6 |
|  |  | 73% | 86% | 59% | 74% | 65% | 49% | 43% | 37% |  |  |  |  |
|  | *k_B_* | 67% | 81% | 49% | 69% | 66% | 49% | 0% | 38% |  |  |  |  |
|  | *k_S_* | 41% | 69% | 32% | 37% | 0% | 0% | 43% | 0% |  |  |  |  |
|  | *k_B_*/(*k_B_*+*k_S_*) | 62% | 54% | 61% | 65% | 100% | 100% | 0% | 100% |  |  |  |  |
|  | *k_S_*/(*k_B_*+*k_S_*) | 38% | 46% | 39% | 35% | 0% | 0% | 100% | 0% |  |  |  |  |
| Coalescent rate measures | *r_B_*+*r_S_* | 3.18 | 7.59 | 1.66 | 3.28 | 2.13 | 1.07 | 0.87 | 5.23 |  |  |  |  |
|  | *r_B_* | 2.26 | 4.72 | 1.08 | 2.51 | 2.13 | 1.07 | 0 | 5.23 |  |  |  |  |
|  | *r_S_* | 0.92 | 2.87 | 0.58 | 0.77 | 0 | 0 | 0.87 | 0 |  |  |  |  |
|  | *r_B_*/(*r_B_*+*r_S_*) | 71% | 62% | 65% | 77% | 100% | 100% | 0% | 100% |  |  |  |  |
|  | *r_S_*/(*r_B_*+*r_S_*) | 29% | 38% | 35% | 23% | 0% | 0% | 100% | 0% |  |  |  |  |

(*) The negative value reflects the fact that the observed diversity level is higher than the level predicted in the absence of linked selection.

# References

1. Mackay TF, Richards S, Stone EA, Barbadilla A, Ayroles JF, Zhu D, Casillas S, Han Y, Magwire MM, Cridland JM, Richardson MF, Anholt RR, Barron M, Bess C, Blankenburg KP, Carbone MA, Castellano D, Chaboub L, Duncan L, Harris Z, Javaid M, Jayaseelan JC, Jhangiani SN, Jordan KW, Lara F, Lawrence F, Lee SL, Librado P, Linheiro RS, Lyman RF, Mackey AJ, Munidasa M, Muzny DM, Nazareth L, Newsham I, Perales L, Pu LL, Qu C, Ramia M, Reid JG, Rollmann SM, Rozas J, Saada N, Turlapati L, Worley KC, Wu YQ, Yamamoto A, Zhu Y, Bergman CM, Thornton KR, Mittelman D, Gibbs RA (2012) The Drosophila melanogaster Genetic Reference Panel. Nature 482: 173-178.

2. Hu TT, Eisen MB, Thornton KR, Andolfatto P (2013) A second-generation assembly of the Drosophila simulans genome provides new insights into patterns of lineage-specific divergence. Genome Res 23: 89-98.

3. Adams MD, Celniker SE, Holt RA, Evans CA, Gocayne JD, Amanatides PG, Scherer SE, Li PW, Hoskins RA, Galle RF, George RA, Lewis SE, Richards S, Ashburner M, Henderson SN, Sutton GG, Wortman JR, Yandell MD, Zhang Q, Chen LX, Brandon RC, Rogers YH, Blazej RG, Champe M, Pfeiffer BD, Wan KH, Doyle C, Baxter EG, Helt G, Nelson CR, Gabor GL, Abril JF, Agbayani A, An HJ, Andrews-Pfannkoch C, Baldwin D, Ballew RM, Basu A, Baxendale J, Bayraktaroglu L, Beasley EM, Beeson KY, Benos PV, Berman BP, Bhandari D, Bolshakov S, Borkova D, Botchan MR, Bouck J, Brokstein P, Brottier P, Burtis KC, Busam DA, Butler H, Cadieu E, Center A, Chandra I, Cherry JM, Cawley S, Dahlke C, Davenport LB, Davies P, de Pablos B, Delcher A, Deng Z, Mays AD, Dew I, Dietz SM, Dodson K, Doup LE, Downes M, Dugan-Rocha S, Dunkov BC, Dunn P, Durbin KJ, Evangelista CC, Ferraz C, Ferriera S, Fleischmann W, Fosler C, Gabrielian AE, Garg NS, Gelbart WM, Glasser K, Glodek A, Gong F, Gorrell JH, Gu Z, Guan P, Harris M, Harris NL, Harvey D, Heiman TJ, Hernandez JR, Houck J, Hostin D, Houston KA, Howland TJ, Wei MH, Ibegwam C, Jalali M, Kalush F, Karpen GH, Ke Z, Kennison JA, Ketchum KA, Kimmel BE, Kodira CD, Kraft C, Kravitz S, Kulp D, Lai Z, Lasko P, Lei Y, Levitsky AA, Li J, Li Z, Liang Y, Lin X, Liu X, Mattei B, McIntosh TC, McLeod MP, McPherson D, Merkulov G, Milshina NV, Mobarry C, Morris J, Moshrefi A, Mount SM, Moy M, Murphy B, Murphy L, Muzny DM, Nelson DL, Nelson DR, Nelson KA, Nixon K, Nusskern DR, Pacleb JM, Palazzolo M, Pittman GS, Pan S, Pollard J, Puri V, Reese MG, Reinert K, Remington K, Saunders RD, Scheeler F, Shen H, Shue BC, Siden-Kiamos I, Simpson M, Skupski MP, Smith T, Spier E, Spradling AC, Stapleton M, Strong R, Sun E, Svirskas R, Tector C, Turner R, Venter E, Wang AH, Wang X, Wang ZY, Wassarman DA, Weinstock GM, Weissenbach J, Williams SM, WoodageT, Worley KC, Wu D, Yang S, Yao QA, Ye J, Yeh RF, Zaveri JS, Zhan M, Zhang G, Zhao Q, Zheng L, Zheng XH, Zhong FN, Zhong W, Zhou X, Zhu S, Zhu X, Smith HO, Gibbs RA, Myers EW, Rubin GM, Venter JC (2000) The genome sequence of Drosophila melanogaster. Science 287: 2185-2195.

4. McDonald JH, Kreitman M (1991) Adaptive protein evolution at the Adh locus in Drosophila. Nature 351: 652-654.

5. Akashi H (1995) Inferring Weak Selection from Patterns of Polymorphism and Divergence at Silent Sites in Drosophila DNA. Genetics 139: 1067-1076.

6. Andolfatto P, Przeworski M (2000) A genome-wide departure from the standard neutral model in natural populations of Drosophila. Genetics 156: 257-268.

7. Smith NG, Eyre-Walker A (2002) Adaptive protein evolution in Drosophila. Nature 415: 1022-1024.

8. Zeng K, Charlesworth B (2010) Studying Patterns of Recent Evolution at Synonymous Sites and Intronic Sites in Drosophila melanogaster. J Mol Evol 70: 116-128.

9. Andolfatto P (2005) Adaptive evolution of non-coding DNA in Drosophila. Nature 437: 1149-1152.

10. Halligan DL, Keightley PD (2006) Ubiquitous selective constraints in the Drosophila genome revealed by a genome-wide interspecies comparison. Genome Res 16: 875-884.

11. Casillas S, Barbadilla A, Bergman CM (2007) Purifying selection maintains highly conserved noncoding sequences in Drosophila. Mol Biol Evol 24: 2222-2234.

12. St Pierre SE, Ponting L, Stefancsik R, McQuilton P, FlyBase C (2014) FlyBase 102--advanced approaches to interrogating FlyBase. Nucleic acids research 42: D780-788.

13. Comeron JM, Ratnappan R, Bailin S (2012) The Many Landscapes of Recombination in Drosophila melanogaster. PLoS Genet 8: e1002905.

14. Chan AH, Jenkins PA, Song YS (2012) Genome-Wide Fine-Scale Recombination Rate Variation in Drosophila melanogaster. PLoS Genet 8: e1003090.

15. Parsch J, Novozhilov S, Saminadin-Peter SS, Wong KM, Andolfatto P (2010) On the utility of short intron sequences as a reference for the detection of positive and negative selection in Drosophila. Mol Biol Evol 27: 1226-1234.

16. Clemente F, Vogl C (2012) Unconstrained evolution in short introns? - an analysis of genome-wide polymorphism and divergence data from Drosophila. Journal of evolutionary biology 25: 1975-1990.

17. Stapleton M, Carlson J, Brokstein P, Yu C, Champe M, George R, Guarin H, Kronmiller B, Pacleb J, Park S, Wan K, Rubin GM, Celniker SE (2002) A Drosophila full-length cDNA resource. Genome biology 3: RESEARCH0080.

18. Stone EA (2012) Joint genotyping on the fly: identifying variation among a sequenced panel of inbred lines. Genome Res 22: 966-974.

19. Cridland JM, Macdonald SJ, Long AD, Thornton KR (2013) Abundance and distribution of transposable elements in two Drosophila QTL mapping resources. Mol Biol Evol 30: 2311-2327.

20. Yang Z (1997) PAML: a program package for phylogenetic analysis by maximum likelihood. Comput Appl Biosci 13: 555-556.

21. Sattath S, Elyashiv E, Kolodny O, Rinott Y, Sella G (2011) Pervasive Adaptive Protein Evolution Apparent in Diversity Patterns around Amino Acid Substitutions in Drosophila simulans. PLoS Genet 7: e1001302.

22. McVicker G, Gordon D, Davis C, Green P (2009) Widespread Genomic Signatures of Natural Selection in Hominid Evolution. PLoS Genet 5: e1000471.

23. Nordborg M, Charlesworth B, Charlesworth D (1996) The effect of recombination on background selection. Genet Res 67: 159-174.

24. Kaplan NL, Hudson RR, Langley CH (1989) The "hitchhiking effect" revisited. Genetics 123: 887-899.

25. The MathWorks I, Natick, Massachusetts, United States MATLAB and Optimization Toolbox Release 2013b.

26. Arlot S, Celisse A (2010) A survey of cross-validation procedures for model selection. Stat Surv 4: 40-79.

27. Coop G, Ralph P (2012) Patterns of Neutral Diversity Under General Models of Selective Sweeps. Genetics 192: 205-U438.

28. Chevin LM, Hospital F (2008) Selective sweep at a quantitative trait locus in the presence of background genetic variation. Genetics 180: 1645-1660.

29. Ralph P, Coop G (2010) Parallel adaptation: one or many waves of advance of an advantageous allele? Genetics 186: 647-668.

30. Pennings PS, Hermisson J (2006) Soft sweeps II--molecular population genetics of adaptation from recurrent mutation or migration. Mol Biol Evol 23: 1076-1084.

31. Pennings PS, Hermisson J (2006) Soft sweeps III: the signature of positive selection from recurrent mutation. PLoS Genet 2: e186.

32. Innan H, Kim Y (2004) Pattern of polymorphism after strong artificial selection in a domestication event. Proc Natl Acad Sci U S A 101: 10667-10672.

33. Przeworski M, Coop G, Wall JD (2005) The signature of positive selection on standing genetic variation. Evolution Int J Org Evolution 59: 2312-2323.

34. Hermisson J, Pennings PS (2005) Soft sweeps: molecular population genetics of adaptation from standing genetic variation. Genetics 169: 2335-2352.

35. Peter BM, Huerta-Sanchez E, Nielsen R (2012) Distinguishing between Selective Sweeps from Standing Variation and from a De Novo Mutation. PLoS Genet 8: e1003011.

36. Berg JJ, Coop G (2015) A Coalescent Model for a Sweep of a Unique Standing Variant. Genetics 201: 707-725.

37. Teshima KM, Przeworski M (2006) Directional positive selection on an allele of arbitrary dominance. Genetics 172: 713-718.

38. Ewing G, Hermisson J, Pfaffelhuber P, Rudolf J (2011) Selective sweeps for recessive alleles and for other modes of dominance. Journal of mathematical biology 63: 399-431.

39. Ewens WJ (2004) Mathematical population genetics. New York: Springer. v. 1.

40. de Vladar HP, Barton N (2014) Stability and Response of Polygenic Traits to Stabilizing Selection and Mutation. Genetics 197: 749-767.

41. Keightley PD, Trivedi U, Thomson M, Oliver F, Kumar S, Blaxter ML (2009) Analysis of the genome sequences of three Drosophila melanogaster spontaneous mutation accumulation lines. Genome Res 19: 1195-1201.

42. Haag-Liautard C, Dorris M, Maside X, Macaskill S, Halligan DL, Houle D, Charlesworth B, Keightley PD (2007) Direct estimation of per nucleotide and genomic deleterious mutation rates in Drosophila. Nature 445: 82-85.

43. Nuzhdin SV, Mackay TF (1995) The genomic rate of transposable element movement in Drosophila melanogaster. Mol Biol Evol 12: 180-181.

44. Charlesworth B (1996) Background selection and patterns of genetic diversity in Drosophila melanogaster. Genet Res 68: 131-149.

45. Houle D, Nuzhdin SV (2004) Mutation accumulation and the effect of copia insertions in Drosophila melanogaster. Genet Res 83: 7-18.

46. Charlesworth B (2012) The Role of Background Selection in Shaping Patterns of Molecular Evolution and Variation: Evidence from Variability on the Drosophila X Chromosome. Genetics 191: 233-246.

47. Comeron JM (2014) Background Selection as Baseline for Nucleotide Variation across the Drosophila Genome. PLoS Genet 10: e1004434.

48. Keightley PD, Eyre-Walker A (2007) Joint inference of the distribution of fitness effects of deleterious mutations and population demography based on nucleotide polymorphism frequencies. Genetics 177: 2251-2261.

49. Wiehe TH, Stephan W (1993) Analysis of a genetic hitchhiking model, and its application to DNA polymorphism data from Drosophila melanogaster. Mol Biol Evol 10: 842-854.

50. Kim Y, Stephan W (2000) Joint effects of genetic hitchhiking and background selection on neutral variation. Genetics 155: 1415-1427.

51. Macpherson JM, Sella G, Davis JC, Petrov DA (2007) Genomewide spatial correspondence between nonsynonymous divergence and neutral polymorphism reveals extensive adaptation in Drosophila. Genetics 177: 2083-2099.

52. Andolfatto P (2007) Hitchhiking effects of recurrent beneficial amino acid substitutions in the Drosophila melanogaster genome. Genome Res 17: 1755-1762.

53. Kliman RM, Hey J (1993) Reduced natural selection associated with low recombination in Drosophila melanogaster. Mol Biol Evol 10: 1239-1258.

54. Comeron JM, Kreitman M, Aguade M (1999) Natural selection on synonymous sites is correlated with gene length and recombination in Drosophila. Genetics 151: 239-249.

55. Betancourt AJ, Presgraves DC (2002) Linkage limits the power of natural selection in Drosophila. Proc Natl Acad Sci U S A 99: 13616-13620.

56. Hey J, Kliman RM (2002) Interactions between natural selection, recombination and gene density in the genes of Drosophila. Genetics 160: 595-608.

57. Marais G, Domazet-Loso T, Tautz D, Charlesworth B (2004) Correlated evolution of synonymous and nonsynonymous sites in Drosophila. J Mol Evol 59: 771-779.

58. Presgraves DC (2005) Recombination enhances protein adaptation in Drosophila melanogaster. Current biology : CB 15: 1651-1656.

59. Zhang Z, Parsch J (2005) Positive correlation between evolutionary rate and recombination rate in Drosophila genes with male-biased expression. Mol Biol Evol 22: 1945-1947.

60. Haddrill PR, Halligan DL, Tomaras D, Charlesworth B (2007) Reduced efficacy of selection in regions of the Drosophila genome that lack crossing over. Genome biology 8: R18.

61. Comeron JM, Williford A, Kliman RM (2008) The Hill-Robertson effect: evolutionary consequences of weak selection and linkage in finite populations. Heredity 100: 19-31.

62. Larracuente AM, Sackton TB, Greenberg AJ, Wong A, Singh ND, Sturgill D, Zhang Y, Oliver B, Clark AG (2008) Evolution of protein-coding genes in Drosophila. Trends in genetics : TIG 24: 114-123.

63. Betancourt AJ, Welch JJ, Charlesworth B (2009) Reduced effectiveness of selection caused by a lack of recombination. Current biology : CB 19: 655-660.

64. Weissman DB, Barton NH (2012) Limits to the rate of adaptive substitution in sexual populations. PLoS Genet 8: e1002740.

65. Shields DC, Sharp PM, Higgins DG, Wright F (1988) "Silent" sites in Drosophila genes are not neutral: evidence of selection among synonymous codons. Mol Biol Evol 5: 704-716.

66. Moriyama EN, Hartl DL (1993) Codon usage bias and base composition of nuclear genes in Drosophila. Genetics 134: 847-858.

67. Nielsen R, Bauer DuMont VL, Hubisz MJ, Aquadro CF (2007) Maximum likelihood estimation of ancestral codon usage bias parameters in Drosophila. Mol Biol Evol 24: 228-235.

68. Andolfatto P, Wong KM, Bachtrog D (2011) Effective population size and the efficacy of selection on the X chromosomes of two closely related Drosophila species. Genome biology and evolution 3: 114-128.

69. Vicario S, Moriyama EN, Powell JR (2007) Codon usage in twelve species of Drosophila. BMC evolutionary biology 7: 226.

70. Haddrill PR, Bachtrog D, Andolfatto P (2008) Positive and negative selection on noncoding DNA in Drosophila simulans. Mol Biol Evol 25: 1825-1834.
